# Survival Factors and Metabolic Pathogenesis in Elderly Patients (≥65) With COVID-19: A Multi-Center Study

**DOI:** 10.3389/fmed.2020.595503

**Published:** 2021-01-07

**Authors:** Qi Mei, Amanda Y. Wang, Amy Bryant, Yang Yang, Ming Li, Fei Wang, Shangming Du, Christian Kurts, Patrick Wu, Ke Ma, Liang Wu, Huawen Chen, Jinlong Luo, Yong Li, Guangyuan Hu, Xianglin Yuan, Jian Li

**Affiliations:** ^1^Tongji Medical College, Tongji Hospital, Huazhong University of Science and Technology, Wuhan, China; ^2^The Renal and Metabolic Division, The George Institute for Global Health, University of New South Wales, Newtown, NSW, Australia; ^3^Concord Clinical School, The University of Sydney, Newtown, NSW, Australia; ^4^Department of Renal Medicine, Concord Repatriation General Hospital, Newtown, NSW, Australia; ^5^Department of Biomedical and Pharmaceutical Sciences, College of Pharmacy, Idaho State University, Meridian, ID, United States; ^6^Renmin Hospital of Wuhan University, Wuhan, China; ^7^Department of Respiratory and Critical Care Medicine, Wuhan Pulmonary Hospital, Wuhan, China; ^8^Department of Chinese Medicine, Tongji Medical College, Wuhan No. 1 Hospital, Huazhong University of Science and Technology, Wuhan, China; ^9^Ludwig Maximilian University of Munich, Munich, Germany; ^10^Institute of Experimental Immunology, University Clinic of Rheinische Friedrich-Wilhelms-University, Bonn, Germany; ^11^Department of Anaesthesiology, Queen Mary Hospital, Hong Kong, China; ^12^Department and Institute of Infectious Disease, Tongji Medical College, Tongji Hospital, Huazhong University of Science and Technology, Wuhan, China; ^13^Department of Emergency Medicine, Tongji Medical College, Tongji Hospital, Huazhong University of Science and Technology, Wuhan, China; ^14^Department of Respiratory and Critical Care Medicine, National Clinical Research Center of Respiratory Disease, Tongji Medical College, Tongji Hospital, Huazhong University of Science and Technology, Wuhan, China

**Keywords:** clinical stratification, elderly patients, mortality, prognostic factors, COVID-19, metabolic pathogenesis, pathway flux, survival factors

## Abstract

**Background:** Elderly patients infected with COVID-19 are reported to be facing a substantially increased risk of mortality. Clinical characteristics, treatment options, and potential survival factors remain under investigation. This study aimed to fill this gap and provide clinically relevant factors associated with survival of elderly patients with COVID-19.

**Methods:** In this multi-center study, elderly patients (age ≥65 years old) with laboratory-confirmed COVID-19 from 4 Wuhan hospitals were included. The clinical end point was hospital discharge or deceased with last date of follow-up on Jul. 08, 2020. Clinical, demographic, and laboratory data were collected. Univariate and multivariate analysis were performed to analyze survival and risk factors. A metabolic flux analysis using a large-scale molecular model was applied to investigate the pathogenesis of SARS-CoV-2 with regard to metabolism pathways.

**Results:** A total of 223 elderly patients infected with COVID-19 were included, 91 (40.8%) were discharged and 132 (59.2%) deceased. Acute respiratory distress syndrome (ARDS) developed in 140 (62.8%) patients, 23 (25.3%) of these patients survived. Multivariate analysis showed that potential risk factors for mortality were elevated D-Dimer (odds ratio: 1.13 [95% CI 1.04 - 1.22], *p* = 0.005), high immune-related metabolic index (6.42 [95% CI 2.66–15.48], *p* < 0.001), and increased neutrophil-to-lymphocyte ratio (1.08 [95% 1.03–1.13], *p* < 0.001). Elderly patients receiving interferon atmotherapy showed an increased probability of survival (0.29 [95% CI 0.17–0.51], *p* < 0.001). Based on these factors, an algorithm (AlgSurv) was developed to predict survival for elderly patients. The metabolic flux analysis showed that 12 metabolic pathways including phenylalanine (odds ratio: 28.27 [95% CI 10.56–75.72], *p* < 0.001), fatty acid (15.61 [95% CI 6.66–36.6], *p* < 0.001), and pyruvate (12.86 [95% CI 5.85–28.28], *p* < 0.001) showed a consistently lower flux in the survivors vs. the deceased subgroup. This may reflect a key pathogenic mechanism of COVID-19 infection.

**Conclusion:** Several factors such as interferon atmotherapy and recreased activity of specific metabolic pathways were found to be associated with survival of elderly patients. Based on these findings, a survival algorithm (AlgSurv) was developed to assist the clinical stratification for elderly patients. Dysregulation of the metabolic pathways revealed in this study may aid in the drug and vaccine development against COVID-19.

## Introduction

Severe Acute Respiratory Syndrome Coronavirus-2 (SARS-CoV-2) is a non-segmented, single-stranded, and positive-sense RNA virus, recently discovered and categorized as the newest and seventh member of the Coronaviride family (1). Following the initial SARS-CoV-2 outbreak started in Dec. 2019 in Wuhan, China, the virus continues to cause a substantial number of casualties worldwide, with more than one million deaths and 35 million confirmed infections (2). The rapidly evolving situation and an increasing number of deaths has created a sense of urgency among scientists, leading to a large number of observational studies characterizing the clinical and epidemiological features of SARS-CoV-2 pneumonia and its spread (3–5). Several recent studies identified advanced age as a common potential risk factor associated with the course of the coronavirus disease 2019 (COVID-19) (6–9). More recently, changes of anatomical respiratory structure with aging (10), immunosenescene (11), and inflammaging (12) may represent three major determinants for a higher prevalence and mortality of COVID-19 infection in elderly populations. Further, age-related alterations in metabolism underlay changes in innate and adaptive immunity (13). However, the role of such metabolic alterations in the pathogenesis of SARS-CoV-2 remains under investigation.

This multi-center study investigated the clinical and epidemiological factors associated with survival in an age-specific (≥ 65 years at diagnosis) cohort and examine flux changes in metabolic pathways to better understand the role of viral metabolic host-dependency in the pathogenesis of SARS-CoV-2. Our findings provide evidence for key virus-host metabolic interactions involved in the pathogenesis in SARS-CoV-2 pneumonia in elderly patients. Such interactions could be targeted for newer vaccine and therapeutic development for these high-risk patients.

## Methods

### Study Design

**Figure 1:**
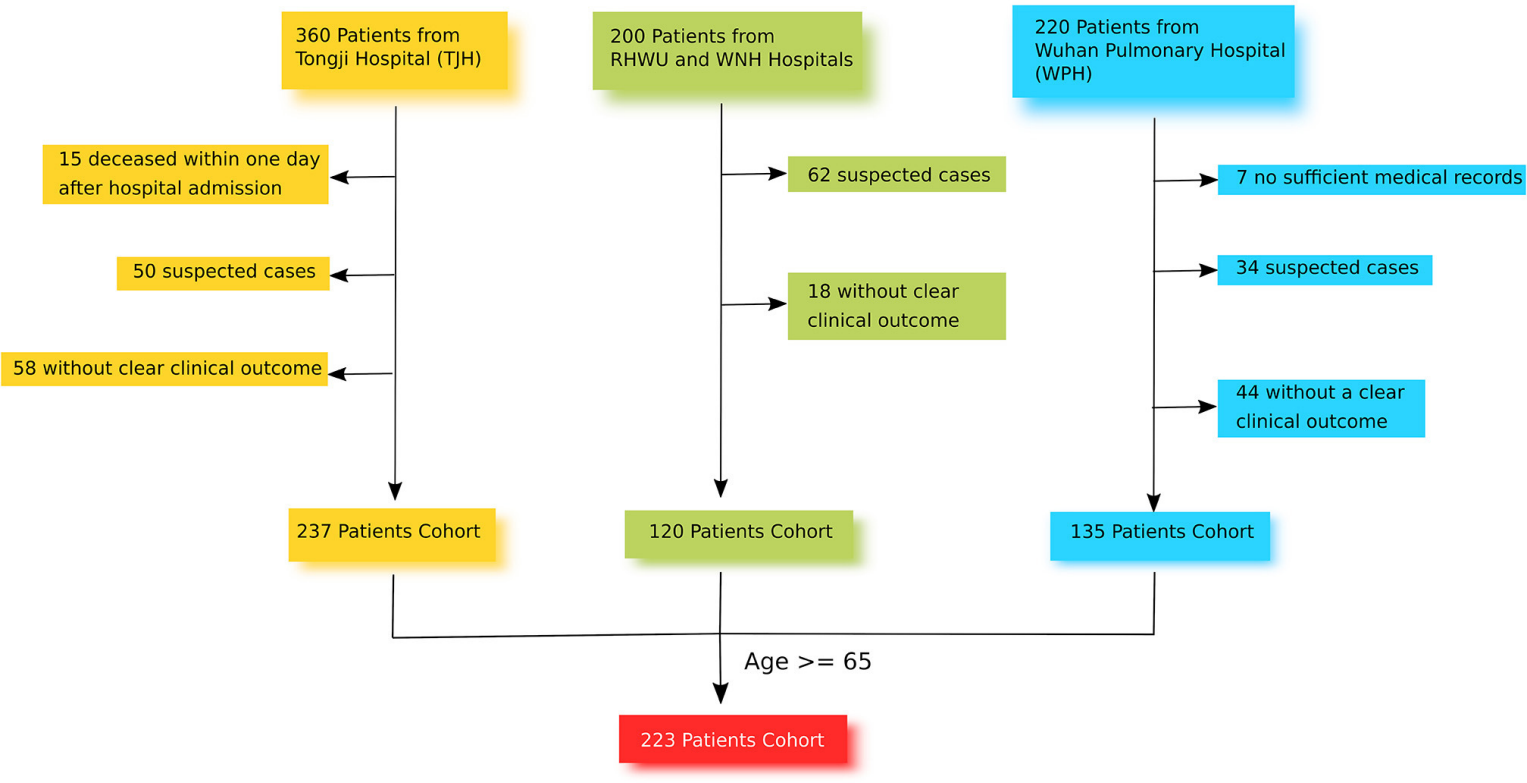
Study Design Chart.

This retrospective and multi-center cohort study was conducted in 4 different hospitals in Wuhan: TJH, RHWU, WPH, and WNH (Figure 1). These 4 hospitals are government designated hospitals assigned to treat patients with SARS-CoV-2 pneumonia. Between January and March 2020, a total of 780 patients with laboratory-confirmed SARS-CoV-2 pneumonia were admitted to these 4 hospitals. The clinical end point for this study was life status (discharged or deceased) at the end of the observation period.

### Patients

From the 780 SARS-CoV-2 pneumonia patients identified, 223 were recruited for this study. Of these, 101 were from TJH, 63 were from WPH, 38 were from WNH, and 21 were from RHWU. The inclusion criteria for study participants were [1] age ≥65 with confirmed diagnosis of SARS-CoV-2 pneumonia at one of the four study sites, [2] positive result of F137nCoV test for SARS-CoV-2 pneumonia, [3] CT-scan with clear evidence of viral pneumonia, [4] available data on the clinical outcome. The exclusion criteria were [1] age <65, [2] deceased within a day after hospital admission, [3] no related health records, [4] no data on clinical outcome, [5] suspected cases without positive result of F137nCoV test, or confirmed cases that were positive using a different test. All recruited participants provided informed consent. Data were recorded on the day of hospital admission in an electronic medical system, and were retrieved and sorted by professional medical staff. If missing values were found, a direct patient interview or telephone communication took place to attempt to obtain the missing information. None of this data has been published previously.

### Treatment Protocol and Discharged Criteria for SARS-CoV-2 Pneumonia

The treatment protocols include either: [1] symptomatic treatment as a basis, active prevention of complications, treatment of underlying diseases, prevention of secondary infections, and timely support of organ vital function, or [2] oxygen-supply therapy including nasal catheter oxygen inhalation, oxygen-supply high nasal flow, invasive and non-invasive mechanical ventilation, or ECMO.

For interferon atmotherapy, patients received 5 × 10^6^ units of interferon α-2b (INFα-2b) nebulized in 4 ml of 0.9% saline with the following parameter: an air-flow rate of 3.0–6.0 L/min, pressure of 90 k−120 kPa, an atomization rate of 350 ± 50 mg/min, and an equivalent volume particle size distribution of MMD: 4.2 ± 25%. Therapy was once daily over 20 min (14, 15) using a compression atomizer (model: BRM-075II).

The criteria for hospital discharge included: [1] stable and normal body temperature for > 3 days, [2] clear reduction in lung inflammation examined via CT-scan, [3] double negative results of the F137nCoV test with at least a 1 day interval, and [4] no comorbidity requiring transfer to another clinical department.

### Detection of SARS-CoV-2 With F137nCoV Test

Upper respiratory tract samples from each patient were collected using nasopharyngeal and oropharyngeal swabs. Synthetic fiber swabs with plastic shafts were used to reduce any contamination risk. Subsequently RNA extraction (2–3 ml) from each sample was performed with a viral nucleic acid isolation kit (Roche). The RNA samples were then purified using QIAquick Gel Extraction Kit (Qiagen). The products were used for a real-time reverse transcription PCR (RT-PCR) assay (F137nCoV), which detected and matched the COVID-19 RNA. A positive result of F137nCoV was defined with Ct (cycle-count threshold)-value <37 while a Ct-value ≥ 40 was deemed a negative result. A Ct-value between 37 and 40 required repeated testing (16).

### Estimation of Metabolic Flux and IM.Index

For the calculation of IM.Index (immune-related metabolic index) of each patient, a large-scale model, MCPM, was applied (17). Laboratory values from each patient were applied as input to initialize the flux of corresponding metabolic reactions (Supplementary Figure 1):

(1) $$\begin{array}{l} {\text{I}}_{\text{j}}\;\left(\text{reaction},\text{ role}\right)\\ =\underset{\text{c}\left(\text{object}\right)}{\prod }\text{ objects }\in \text{ reactant }\left(\text{reaction},\text{ role}\right).\text{objects} \end{array}$$

role ∈ {e, g, i, s, tr(a), tr(r)}, (e: enzyme; g: gene; i: inhibitor; s: substrate; tr(a): transcriptional activator, tr(r): transcriptional repressor).

Subsequently, the flux of a metabolic pathway, flux(P), was calculated as the sum of intensities in reactions within this pathway deducted by the intensities of reactions going out into crosstalk pathways.

(3) $$\begin{array}{l} \text{flux}\left(\text{P}\right)=\left[\sum {\text{I}}_{\text{i}}\left(\text{reactio}{\text{n}}_{\text{i}\in \text{P}},\text{role}\right)/\text{N}\left(\text{P}\right)\right]-\text{flux}\left[\text{Crosstalk}\left(\text{P}\right)\right] \end{array}$$

N(P): the number of reactions of the pathway P; Crosstalk(P): the crosstalk pathways related to the pathway P.

IM.Index[patient] = ∑ flux(p)/N(P) (Supplementary Information 1).

### Statistical Considerations

The time interval for overall survival was calculated starting from the date of hospital admission until death from SARS-CoV-2 pneumonia or the date of the last follow-up. An event was considered death due to SARS-CoV-2 pneumonia. Mortality was determined via the vital status of each patient at the end of the study observation period. For data analysis continuous variables were reported as median (IQR) and categorical variables were shown as frequency or percentage. The least absolute shrinkage and selection operator (LASSO) was used for multivariable selection (18). The area under curve (AUC) value was used to evaluate the accuracy of the vital status prediction. The Cox proportional hazards regression analysis was used to evaluate the prediction of a prognostic model for overall survival. Proportional hazards assumption for the Cox proportional hazards regression model was assessed via the Schoenfeld residuals test. The 95% confidence intervals (CIs) were estimated via 5,000 bootstraps replicates. Propensity score matching (PSM) was performed to adjust demographic factors (including age, sex, comorbidities), survival status and treatments. All statistical analyses were performed using R (version 3.6) and SAS (version 9.4). A *p* < 0.05 was considered as statistically significant.

### Role of Funding

The funders were not involved in any activities of this study, aside from providing financing.

## Results

### Demographic and Clinical Features in the Elderly Cohort

The elderly cohort (age ≥65 years old) consisted of 223 patients, which were admitted between Jan. and Mar. 2020 to four different Wuhan hospitals: 101 from TJH, 63 from WPH, 38 from WNH, and 21 from RHWU. As shown in Table 1, 132 (59.2%) patients died during hospitalization, 91 (40.8%) recovered and attained discharge criteria. The median age of this elderly cohort was 72.0 year (IQR 68.0–77.5). There was no significant age and body mass index (BMI) difference between both subgroups (survival vs. deceased). Gender was almost equally distributed (50.2% [M] vs. 49.8% [F]). Thirty-five (15.7%) patients were treated in an intensive care unit (ICU), and one of these patients was subsequently discharged. In 140 (62.8%) patients, symptoms of acute respiratory distress syndrome (ARDS) developed with 23 (25.3%) of these patients recovering. The majority of patients (74.4%) were affected by underlying diseases, hypertension (53.4%) being the most common (Table 1). The most common initial symptoms were fever (69.5%), cough (51.1%), and dyspnea (31.4%). The median body temperature on admission was 36.7°C (IQR 35.6–37.2), no difference of median temperature between both subgroups (survival vs. decreased) was seen. Similarly, both subgroups reported the same median interval (10 days) from symptom onset to hospital admission (Table 1). The hospitalization in the surviving subgroup was significantly longer than that of the decreased subgroup (20.0 days [IQR 15.0–25.0] vs. 8.0 days [IQR 5.0–13.0]). The majority of patients received antiviral treatments (lopinavir/ritonavir; 96%), antibiotics (86.1%), and corticosterioids (71.3%). Additionally, almost half of patients received interferon (47.1%) and immuneglobulin (48.0%) treatment. In this cohort, the most frequently applied oxygen therapies were UOC (84.8%) and NIMV (43.9%). All key laboratory findings of this cohort are listed in Table 1.

**Table 1:** Clinical characteristics, treatments and laboratory findings.

	**Overall**	**Survivor**	**Deceased**	***P-value***
**Characteristics**				
Number of patients	223	91 (40.8)	132 (59.2)	…
Median age, years	72.0 (68.0–77.5)	70.0 (67.0–74.5)	74 (69.0–80.0)	<0.001
Sex	…	…	…	0.077
Male	112 (50.2)	39 (42.9)	73 (55.3)	…
Female	111 (49.8)	52 (57.1)	59 (44.7)	…
Body mass index (BMI)	23.2 (20.0–24.9)	23.3 (20.1–25.2)	23.1 (20.0–24.8)	0.751
ICU care	…	…	…	<0.001
Yes	35 (15.7)	1 (1.1)	34 (25.8)	…
No	188 (84.3)	90 (98.9)	98 (74.2)	…
Smoking	…	…	…	0.682
Yes	27 (12.1)	12 (13.2)	15 (11.4)	…
No	196 (87.9)	79 (86.8)	117 (88.6)	…
ARDS	140 (62.8)	23 (25.3)	117 (88.6)	
IM.Index	9.38 (9.06–9.78)	9.14(8.86–9.34)	9.64 (9.30–10.00)	<0.001
Comorbidities	166 (74.4)	67 (73.6)	99 (75.0)	0.876
Hypertension	119 (53.4)	41 (45.1)	78 (59.1)	0.042
CVDs	33 (14.8)	10 (11.0)	25 (17.4)	0.249
Diabetics	52 (23.3)	17 (18.7)	35 (26.5)	0.199
Carcinoma	9 (4.0)	3 (6.6)	3 (2.3)	0.164
Initial common symptome	213 (95.5)	86 (94.5)	127 (96.2)	0.744
Fever	155 (69.5)	59 (64.8)	96 (72.7)	0.237
Cough	114 (51.1)	44 (48.4)	70 (53.0)	0.499
Myalgia or Fatigue	43 (19.3)	15 (16.5)	28 (21.2)	0.395
Dyspnea	70 (31.4)	19 (20.9)	51 (38.6)	0.005
Admission body temperature, °C	36.7 (36.5–37.2)	36.7 (36.5–37.2)	36.7 (36.5–37.2)	0.883
Symptom onset to admission, days	10.0 (7.0–14.0)	10.0 (7.0–14.0)	10.0 (6.0–14.0)	0.647
Hospitalization, days	13.0 (7.0–20.0)	20.0 (15.0–25.0)	8.0 (5.0–13.0)	<0.001
Systolic pressure, mm Hg	131.0 (120.0–146.0)	128.0 (120.5–141.0)	133 (120.0–148.5)	0.152
Respiratory rate, breaths per min	22.0 (20.0–26.0)	20.0 (20.0–25.0)	22 (20.0–29.3)	0.022
Pulse rate, beats per min	88.0 (80.0–100.5)	84.0 (78.0–96.0)	90 (80.0–103.0)	0.003
**Treatments**				
Therapy	…	…	…	…
Antibiotics	192 (86.1)	65 (71.4)	127 (96.2)	<0.001
Antiviral treatment	214 (96.0)	65 (71.4)	124 (93.9)	0.086
Corticosteroids	159 (71.3)	57 (62.6)	102 (77.3)	0.024
Interferon atmotherapy	105 (47.1)	59 (64.8)	46 (34.8)	<0.001
Immunoglobin	107 (48.0)	32 (35.2)	75 (56.8)	0.002
Oxygen therapy	213 (95.5)	82 (90.1)	131 (99.2)	0.002
UOC	189 (84.8)	82 (90.1)	107 (81.1)	0.087
HFNO	30 (13.5)	20 (22.0)	10 (7.6)	0.003
NIMV	98 (43.9)	7 (7.7)	91 (68.9)	<0.001
IMV	39 (17.5)	1 (1.1)	38 (28.8)	<0.001
ECMO	5 (2.2)	0	5 (3.8)	0.081
**Laboratory findings**				
WBC count, × 10^9^/L	…	…	…	<0.001
<3.5	19 (8.5)	9 (9.9)	10 (7.6)	…
3.5-9.5	128 (57.4)	70 (76.9)	58 (43.9)	…
>9.5	76 (34.1)	12 (13.2)	64 (48.5)	…
Neutrophil count, × 10^9^/L	…	…	…	<0.001
<1.8	9 (4.0)	5 (5.5)	4 (3.0)	…
1.8–6.3	107 (48.0)	64 (70.3)	43 (32.6)	…
>6.3	107 (48.0)	22 (24.2)	85 (64.4)	…
Lymphocyte count, × 10^9^/L	…	…	…	0.000
<1.1	180 (80.7)	63 (69.2)	117 (88.6)	…
≥1.1	43 (19.3)	28 (30.8)	15 (11.4)	…
PLT count, × 10^9^/L	…	…	…	<0.001
<125	57 (25.6)	10 (11.0)	47 (35.6)	…
125–350	157 (70.4)	76 (83.5)	81 (61.4)	…
>350	9 (4.0)	5 (5.5)	4 (3.0)	…
APTT, s	…	…	…	0.017
<29	18 (8.1)	3 (3.3)	15 (11.4)	…
29–42	155 (69.5)	72 (79.1)	83 (62.9)	…
>42	50 (22.4)	16 (17.6)	34 (25.8)	…
PT, s	…	…	…	<0.001
≤14.5	139 (62.3)	75 (82.4)	64 (48.5)	…
>14.5	84 (37.7)	16 (17.6)	68 (51.5)	…
D-D dimer, ug/ml FEU	…	…	…	<0.001
<0.5	50 (22.4)	39 (42.9)	11 (8.3)	…
≥0.5	173 (77.6)	52 (57.1)	121 (91.7)	…
ALT, U/L	…	…	…	0.553
<33 (female) or 41 (male)	156 (70.0)	66 (72.5)	90 (68.2)	…
≥33 (female) or 41 (male)	67 (30.0)	25 (27.5)	42 (31.8)	…
AST, U/L	…	…	…	<0.001
<32 (female) or 40 (male)	112 (50.2)	61 (67.0)	51 (38.6)	…
≥32 (female) or 40 (male)	111 (49.8)	30 (33.0)	81 (61.4)	…
LDH, U/L	…	…	…	<0.001
≤214 (female) or 225 (male)	25 (11.2)	20 (22.0)	5 (3.8)	…
>214 (female) or 225 (male)	198 (88.8)	71 (78.0)	127 (96.2)	…
ALP, U/L	…	…	…	<0.001
<35 (female) or 40 (male)	12 (5.4)	7 (7.7)	5 (3.8)	…
35–105 (female) or 40–130 (male)	185 (83.0)	83 (91.2)	102 (77.3)	…
>105 (female) or 130 (male)	26 (11.7)	1 (1.1)	25 (18.9)	…
γ-GT, U/L	…	…	…	0.224
<6 (female) or 10 (male)	2 (0.9)	2 (2.2)	0	…
6–42 (female) or 10–71 (male)	170 (76.2)	70 (76.9)	100 (75.8)	…
>42 (female) or 71 (male)	51 (22.9)	19 (20.9)	32 (24.2)	…
Urea, mmol/L	…	…	…	<0.001
<2.6 (female) or 3.6 (male)	10 (4.5)	5 (5.5)	5 (3.8)	…
2.6–7.5 (female) or 3.6–9.5 (male)	130 (58.3)	72 (79.1)	58 (43.9)	…
>7.5 (female) or 9.5 (male)	83 (37.2)	14 (15.4)	69 (52.3)	…
Albumin, g/L	…	…	…	<0.001
<35	152 (68.2)	48 (52.7)	104 (78.8)	…
≥35	71 (31.8)	43 (47.3)	24 (18.2)	…
Total cholesterol, mmol/L	…	…	…	1.000
<5.18	212 (95.1)	87 (95.6)	125 (94.7)	…
≥5.18	11 (4.9)	4 (4.4)	7 (5.3)	…
Total bilirubin, μmol/L	…	…	…	0.009
<21 (female) or 26 (male)	198 (88.8)	87 (95.6)	111 (84.1)	…
≥21 (female) or 26 (male)	25 (11.2)	4 (4.4)	21 (15.9)	…
hs-CRP, mg/L	…	…	…	<0.001
<10.0	26 (11.7)	21 (23.1)	5 (3.8)	…
≥10.0	197 (88.3)	70 (76.9)	127 (96.2)	…

*Data are median (IQR), or n (%). ICU, Intensive care unit; ARDS, Acute respiratory distress syndrome; UOC, Usual oxygen care, including standard nasal catheter and facemask inhalation; HFNO, High-flow nasal cannula oxygen; (N)IMV, (Non-) Invasive mechanical ventilation; ECMO, Extracorporeal membrane oxygenation; WBC, White blood cell; PLT, Blood platelet; APTT, Activated partial thromboplastin time; PT, Prothrombin time; ALT, Alanine aminotransferase; AST, Aspartate aminotransferase; LDH, Lactate dehydrogenase; ALP, Alkaline phosphatase; γ-GT, gamma-Glutamyl transpeptidase; hs-CRP, high-sensitivity C-reactive protein*.

### Potential Survival Factors Associated With Vital Status for Elderly Patients With COVID-19

The results of a univariate analysis showed that the odds of survival for elderly patients with COVID-19 were higher in patients having elevations in lymphocyte count, albumin, creatinine, and blood platelet count (Table 2). Among the recorded comorbidities only hypertension was significantly associated with survival (*p* = 0.042), others such as diabetes, carcinoma, and coronary diseases did not influence survival in this cohort (Table 2). Drug treatments including antibiotics (OR: 10.16 [95% CI 3.73–27.69], *p* < 0.001), corticosteroids (OR: 2.03 [95% CI 1.13–3.65], *p* = 0.018) and immunoglobulin (OR: 2.43 [95% CI 1.4–4.21], *p* = 0.002) negatively influenced survival in this cohort, whereas interferon atmotherapy (OR: 0.29 [95% CI 0.17–0.51], *p* < 0.001) showed a positive association with survival. Other potential factors were listed in Table 2. Using a multivariate analysis in this cohort (132 survivals vs. 91 decreased), the results showed that three significant survival factors were identified, including decreased D-Dimer, decreased IM.Index, decreased neutrophil-to-lymphocyte ratio (Table 2).

**Table 2:** Survival/Risk factors associated with mortality in age-specific patient cohort infected with COVID-19.

**Univariate analysis**	**AOR (95%CI)**	**Wald's *P*-value**
**Demographic and clinical characteristics**		
Hypertension: 1 vs. 0	1.76 (1.03, 3.02)	0.040
Respiratory rate, breaths per min	1.08 (1.02, 1.13)	0.006
Pulse rate, beats per min	1.03 (1.01, 1.05)	0.001
**Treatments**		
Antibiotics: 1 vs. 0	10.16 (3.73, 27.69)	<0.001
Corticosteroids: 1 vs. 0	2.03 (1.13, 3.65)	0.018
Interferon atmotherapy: 1 vs. 0	0.29 (0.17, 0.51)	<0.001
Immunoglobulin: 1 vs. 0	2.43 (1.4, 4.21)	0.002
Non-invasive mechanical ventilation (NIMV): 1 vs.0	27.3 (11.6, 64.26)	<0.001
Invasive mechanical ventilation (IMV): 1 vs.0	36.77 (4.94, 273.53)	<0.001
**Immune components**		
White blood cell (WBC) count, × 10^9^/L	1.23 (1.14, 1.34)	<0.001
Neutrophil count, × 10^9^/L	1.28 (1.17, 1.41)	<0.001
Lymphocyte count, × 10^9^/L	0.2 (0.1, 0.41)	<0.001
Neutrophil ratio	1.08 (1.03, 1.21)	<0.001
Lymphocyte ratio	0.12 (0.08, 0.23)	<0.001
Neutrophil/lymphocyte ratio	1.12 (1.08, 1.17)	<0.001
IM.Index	13.82 (6.19, 30.83)	<0.001
**Other laboratory findings**		
Alanine transaminase (ALT), U/L	1.01 (1, 1.03)	0.049
Aspartate aminotransferase (AST), U/L	1.04 (1.02, 1.06)	<0.001
Alkaline phosphatase (ALP), U/L	1.02 (1.01,1.04)	<0.001
Lactate dehydrogenase (LDH), U/L	1.0063 (1.0042, 1.0085)	<0.001
gamma-Glutamyl transpeptidase (γ-GT), U/L	1.007 (1.0002, 1.0139)	0.044
Total bilirubin, μmol/L	1.11 (1.06, 1.17)	<0.001
Albumin, g/L	0.83 (0.77, 0.89)	<0.001
Creatine kinase (CK), U/L	1.0078 (1.0009, 1.0147)	0.026
Urea, mmol/L	1.11 (1.05, 1.17)	<0.001
Uric acid, μmol/L	1.0025 (1.0005, 1.0045)	0.013
K+, mmol/L	1.63 (1.08, 2.46)	0.020
Na+, mmol/L	1.06 (1.01, 1.11)	0.025
Cl -, mmol/L	1.05 (1, 1.09)	0.039
P, mmol/L	9.36 (2.26, 38.82)	0.002
Mg2+, mmol/L	17.12 (3.1, 94.6)	0.001
high-sensitivity C-reactive protein (hs-CRP), mg/L	1.02 (1.01, 1.03)	<0.001
Creatinine, umol/L	0.98 (0.97, 0.99)	<0.001
Blood platelet (PLT) count, × 10^9^/L	0.9928 (0.9893, 0.9963)	<0.001
Prothrombin time (PT), s	1.24 (1.08, 1.42)	0.003
D-D dimer, μg/mL FEU	1.21 (1.12, 1.32)	<0.001
**Multivariate analysis**	**AOR (95%CI)**	**Wald's** ***P*****-value**
Number of events/patients (%)	132/223 (59.2%)	…
D-D dimer, μg/mL FEU	1.13 (1.04–1.22)	0.005
IM.Index	6.42 (2.66–15.48)	<0.001
Neutrophil-to-lymphocyte ratio	1.08 (1.03–1.13)	<0.001
**Covariate**	**Coefficient**	**Score**
D-D dimer, μg/mL FEU	2	2 × D-D dimer
IM.Index	10	10 × IM.Index
Neutrophil-to-lymphocyte ratio	1	1 × ratio
**Total computed score and risk stratification**		
Low risk		≤ 10^6^
High risk		> 10^6^

*AOR, Adjusted odds ratio; CI, Confidence interval*.

### Pathological Alternation From the Admission to Clinical Endpoint

**Figure 2:**
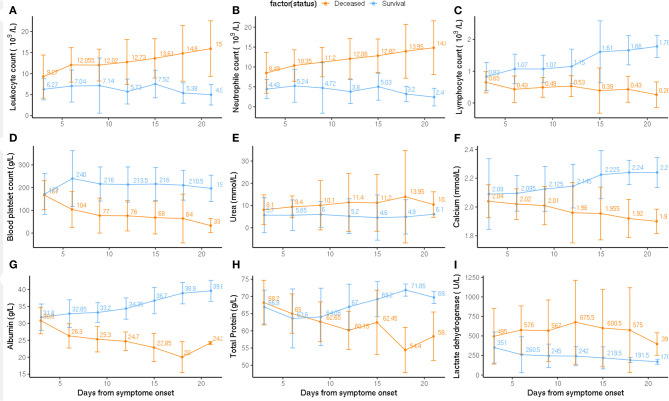
Pathological alternation from the admission to clinical endpoint between deceased and survival subgroups. Timeline charts illustrate the laboratory parameters (**A**: Leukocyte count, **B**: Neutrophil count, **C**: Lymphocyte count, **D**: Blood platelet count, **E**: Urea, **F**: Calcium, **G**: Albumin, **H**: Total Protein, **I**: Lactate dehydrogenase) in elderly COVID-19 patients during defined period of clinical observation. Survival and deceased groups are symbolized with dark orange and blue color.

Laboratory markers regarding immunity, inflammation, metabolism, and other physiological functions were tracked from admission to the end point of clinical observation (Figure 2). Baseline counts of leukocytes and neutrophils increased continuously in deceased subgroup compared to the survival subgroup; whereas severe lymphopenia occurred in deceased subgroup. Levels of blood platelet count, calcium, and albumin were continuously reduced in the deceased subgroup (Figure 2). From 10 days on, the level of total protein significantly decreased in deceased than in survival subgroup. The level of urea and lactate dehydrogenase were moderately higher in deceased than in survival subgroup (Figure 2).

### Metabolic Alteration Between Deceased and Survival in COVID-19

**Figure 3:**
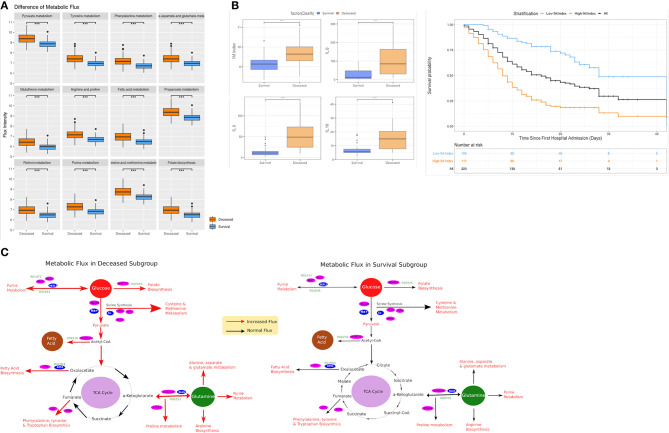
Comparison of metabolic flux and IM.Index. **(A)** Difference of metabolic flux in 12 metabolic pathways between survival and deceased subgroup in the elderly cohort (≥ 65 years old); **(B)** Differences of IM.Index, IL6, IL8, and IL10 in survival vs. deceased subgroups, and the clinical differentiation of IM.Index in the cohort. **(C)** Visualization of metabolic flux in survival vs. deceased subgroup (a more comprehensive view in Supplementary Figure 1). ***P = 0.001, **P = 0.01, *P = 0.05.

**Figure 4:**
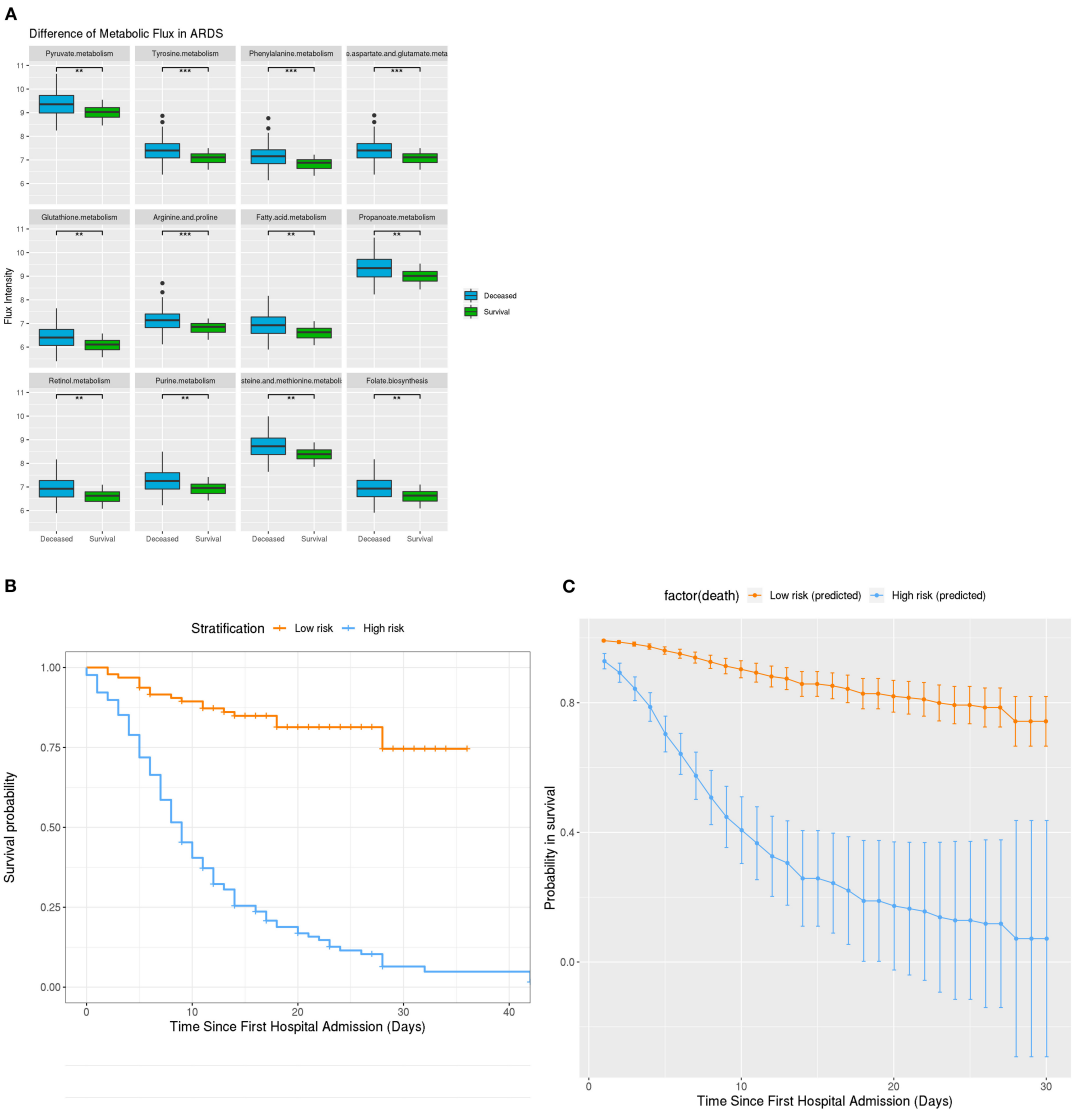
Metabolic Difference in ARDS and Clinical stratification on the basis of developed survival algorithm AlgSurv. **(A)** Flux difference in 9 different metabolic pathways between deceased and survival subgroups with ARDS. **(B)** OS in the low- and high-risk subgroups defined by a cutoff of < 10^6^ and ≥ 10^6^. **(C)** Predicted OS rates in the elderly cohort. Smooth lines represent mean predicted OS probabilities for each risk group. ***P = 0.001, **P = 0.01, *P = 0.05.

We hypothesized a significant difference in metabolic pathway utilization could exist between deceased and surviving subgroups. Thus, a genome-scale metabolic flux analysis was conducted for both subgroups. The results showed that fluxes from 12 metabolic pathways in the deceased patients were consistently and significantly higher than that of the surviving patients (Figure 3A). These changes were seen in the phenylalanine- (OR: 28.27 [95% CI 10.56–75.72], *p* < 0.001), tyrosine- (25.81 [95% CI 9.8–67.99], *p* < 0.001), glutathionie- (17.23 [95% CI 7.16–41.46], *p* < 0.001), purine- (16.34 [95% CI 6.88–38.88], *p* < 0.001), fatty acid- (15.61 [95% CI 6.66–36.6], *p* < 0.001), pyruvate- (12.86 [95% CI 5.85–28.28], *p* < 0.001) -metabolic pathways, among others (Table 3). Similar differences were observed between the patients surviving after ARDS and those who did not (Figure 4A). Differences in metabolic flux was not observed between genders. The results of metabolic flux analysis also showed that the immune-related metabolic index (IM.Index) was significantly lower in the surviving subgroup than that of the deceased subgroup (9.14 [95% CI 8.86–9.34] vs. 9.64 [95% 9.3–10.0]; Table 1, Figure 3C). Further, circulating levels of three cytokines (IL6, IL8, and IL10) were significantly increased in the deceased subgroup (Figure 3B).

**Table 3:** Odds ratio of metabolic pathway in age-specific patient cohort infected with COVID-19.

**Pathway survival analysis**	**AOR (95%CI)**	**Wald's *P*-value**
**Metabolic pathway**		
Phenylalanine metabolism	28.27 (10.56, 75.72)	<0.001
Arginine and proline	27.68 (10.38, 73.84)	<0.001
Tyrosine metabolism	25.81 (9.8, 67.99)	<0.001
Alanine aspartate and glutamate metabolism	25.72 (9.77, 67.69)	<0.001
Glutathione metabolism	17.23 (7.16, 41.46)	<0.001
Cysteine and methionine metabolism	16.78 (7.14, 39.41)	<0.001
Folate biosynthesis	16.38 (6.9, 38.88)	<0.001
Purine metabolism	16.34 (6.88, 38.81)	<0.001
Steroid biosynthesis	15.62 (6.66, 36.62)	<0.001
Retinol metabolism	15.62 (6.66, 36.61)	<0.001
Fatty acid metabolism	15.61 (6.66, 36.6)	<0.001
Pyruvate metabolism	12.86 (5.85, 28.28)	<0.001
Propanoate metabolism	12.82 (5.84, 28.15)	<0.001

*AOR, Adjusted odds ratio; CI, Confidence interval*.

### Algorithm for Survival Prediction in Elderly Patients With COVID-19

Given the high mortality rate of elderly patients, an algorithm (AlgSurv) to predict survival was developed based on the weight of the three significant predictors (Table 2). The AlgSurv yielded an AUC of 0.863 (95% CI 0.817–0.909) in successfully predicting survival in this cohort as well as a Harrell's c-index of 0.769 (95% CI 0.73–0.808) for overall survival. In this elderly cohort, the AlgSurv was able to define a high-risk subgroup with a significantly lower prognosis (hazard ratio: 5.85 [95% CI 3.76–9.08]) vs. a low-risk subgroup with a high probability of survival (Figure 4B). The predicted 30-days survival rates of the high- and low-risk subgroups in this cohort is illustrated in Figure 4C (cutoff ≥ 10^6^ and <10^6^).

## Discussions

Elderly patients infected with COVID-19 have an increased risk of mortality which can be seen by the death rate in this multi-center study being 59.2%. In this cohort of patients, 62.8% developed acute respiratory distress syndrome (ARDS) with a total of 25.3% patients recovering. Underlying comorbidities were assessed to determine any association with mortality. Among them, hypertension was commonest comorbidity (54.3%) and was the only factor significantly associated with survival. Other identifiable survival factors of COVID-19 infection in this elderly cohort were use of interferon atmotherapy, reduced activity reduction of metabolic pathways and specific biochemical markers trends (i.e., decreased D dimer, decreased IM index, decreased neutrophil to lymphocyte ratio). Based on these factors, an algorithm AlgSurv was developed, which yielded an AUC of 0.863 for predicting to outcome and C-index of 0.769 for overall survival, respectively.

The complicated virus/host interactions involved in the pathogenesis and outcome of SARS-CoV-2 remain under investigation. It was hypothesized that like other coronaviruses SARS-CoV-2 may evolve to alter metabolic pathways in its host to meet a drastically increasing demand of resource for replication and expansion (19, 20). In this study, a system-level metabolic flux analysis was performed to assess changes in metabolic pathways that were characteristic for older patients surviving the infection vs. those that did not. The fluxes of 12 metabolic pathways were consistently lower in surviving vs. the deceased subgroup. All of these metabolic pathways were involved with energy and nutrient metabolism such as glucose (21), fatty acid (22) and nucleic acid (23). This finding provides important evidence that host metabolism may play an important role in the general pathogenesis of COVID-19.

As the virus replicates, large amounts of energy and nutrients are needed and obtained from the metabolism of the host cells (24). In cellular metabolism, pyruvate metabolism connects ATP production from the citric acid cycle with oxidative phosphorylation pathways and many biosynthetic pathways (25). Moreover, this pathway was recently found to be a predictor of outcome follow a H7N9 virus infection (26). In our cohort, the flux of fatty acid metabolism was significantly increased in the deceased vs. surviving subgroup. Its upregulation could indicate an enhanced virus replication that parallels deterioration of host health. Of note, purine release and metabolism has a high impact on inflammatory responses relating to cytokine signaling (27). The enhanced activity of purine metabolism in the deceased subgroup may indicate an overly strong inflammatory response at the system level, as evidenced by the hyper-elevated cytokine production in this subgroup. Such a “cytokine storm” may thereby decrease the probability for survival.

Phenylalanine metabolism was observed to be highly upregulated in the deceased subgroup. These findings indicate that SARS-CoV-2 may function in a similar way as the respiratory syncytial virus (28) in utilizing the phenylalanine residue for the production of internal viral protein and its subsequent assembly into viral particles to deteriorate the pneumonia in its host. Such a finding would suggest continued viral replication and could be exploited to differentiate between SARS-CoV-2 PCR-positive patients that have resolved infection from those that remain contagious.

Relatively low expressed fluxes in these metabolic pathways in the surviving subgroup may therefore imply that virus replication and related inflammatory responses were not as extensive as that in the deceased subgroup. This may significantly contribute to survival in elderly patients with COVID-19. Additionally, no significant difference of BMI between both subgroups ruled out the possibility that the elevated metabolic flux (activity) may have been caused by pre-existing deregulated metabolic states, ahead the COVID-19 infection.

The immune-related metabolic index (IM.Index) was used to summarize the metabolic flux in a genome-scale model and shown to be significantly lower in the surviving patients. These results suggest that abnormally high metabolic activity during COVID-19 infection may be associated with a weakened immune function and a lower probability of survival. Our results are in line with recent discoveries, demonstrating that a regulated metabolism could be actually a driving force for innate and adaptive immunity (13, 29, 30). Although genetically-based, or acquired, immune impairment worsen the COVID-19 disease course (31, 32), our results further suggest that a dysregulation of metabolism may also impair immunity contributing to fatal outcome during COVID-19, especially for elderly patients.

Further, the metabolic fluxes of 23 (25.3%) patients surviving ARDSs were also significantly lower than in those not surviving ARDSs. These results imply that patients suffering from ARDS and associated with relatively low metabolic activities have an increased probability for survival.

The majority of patients in this study received antivirals (lopinavir/ritonavir), antibiotics, and corticosteroids. The univariate analysis results showed that application of antibiotics (OR: 10.16) and corticosteroids (OR: 2.03) did not improve survival in this cohort. Only a dose reduction in corticosteroids during the course of treatment had a positive influence on survival. In contrast, patients receiving the interferon atmotherapy (OR: 0.29) showed a better prognosis in this cohort. Although this finding is consistent with outcomes of two recent clinical trials (33, 34), future large-cohort studies are warranted to address the efficacy and side-effect of interferon based treatment on COVID-19 patients with a severe and critical ill disease course. The majority of patients (74.4%) in this cohort had underlying comorbidities. Although hypertension (*p* = 0.042) was slightly related to mortality, diabetes, carcinoma, and coronary heart diseases were not. This contradicts many recent studies that reported a significant association of underlying diseases with the mortality of patients with COVID-19 (35–37). The reason for this discrepancy could be that advanced age could be a risk factor associated with the mortality of COVID-19, and comorbidity itself could only be an independent factor associated with advanced age. For instance, elderly individuals are more likely to have chronic kidney disease and diabetes (38, 39). On the one hand, such diseases lowers the person's immunity, and when treated with angiotensin converting enzyme 2 (ACE-2) inhibitors or angiotensin-receptor blockers (ARBs) that upregulate ACE-2 receptor (20), could enable a more aggressive SARS-CoV-2 virus entry. In our age-specific cohort study, the influence of advanced age was drastically reduced and therefore the age related dependency of comorbidity was compromised, leading to its dissociation with observed mortality.

Of note, no difference in the time interval from symptom onset to hospital admission was observed between survival and deceased subgroups. Similarly, no difference in body temperature on admission between both subgroups was observed. These results indicated guidelines recommending hospital admission within 10 days and body temperature between 36.5 and 37.2 on admission may be unrelated to the survival of elderly patients with COVID-19.

This study has several strengths. First of all, it provides a comprehensive scientific discussion of COVID-19 that are clinically important and relevant to COVID-19 during this pandemic setting. To our knowledge, it is the first paper focusing on metabolic pathogenesis in the elderly population who are amongst the highest risk patients for severe complications of COVID-19. Furthermore, there is limited literature of this specific population given the novelty of the illness. Therefore, early recognition of at-risk elderly individuals, stratifying their risk profiles (i.e., comorbidities) and developing appropriate drug therapy to be prescribed in a timely matter is critical to reduce the mortality of COVID-19. Secondly, this paper assists a possible understanding of COVID-19 pathophysiology and explores how the deregulation of metabolic pathways can serve as a potential target for further drug development and may assist in future studies. Finally, it aims to identify important survival factors, such as prescription of interferon atmotherapy treatment, which has the potential for being an effective novel therapy.

This study has several limitations. First, the median age of this cohort is 72 years old, which may not reflect the whole elderly population. It is a small population study, which could be underpowered. Secondly, we focused only on elderly patients (≥65) infected with COVID-19 in Wuhan, China, which may not be representative of the whole population in China. The case-fatality rate in this study was therefore the highest among all reported studies to the present, and therefore likely much higher than the actual mortality of COVID-19 worldwide. Thirdly, the developed AlgSurv needs to be further validated with a large international cohort. Further, the treatment benefit of interferon atmotherapy was investigated in this cohort through PSM. However, PSM is not a substitute of a randomized study (40) and this finding therefore needs to be verified in a randomized control trial. Finally, we acknowledge that metabolic pathway's activity cannot actually assist the clinician in real-time, however, better understanding of this metabolic pathway can potentially assists in development of a target therapy for this pandemic disease. The discovered metabolic mechanisms related to the pathogenesis of COVID-19 were derived from this age-specific cohort. Follow-up studies will need to verify this issue in other cohorts. IM.Index was initially developed analyzing RNA sequencing data (under review). Under the emergency situation of this pandemic, this score index was applied to the analysis of the laboratory blood values of COVID-19 patients. Therefore, the impact of this index score applied here may different from that when analyzing RNA sequencing data.

## Conclusion

Our study investigated an age-specific elderly patient cohort suffering from COVID-19. Although a high mortality has been reported for these patients, several potential survival factors were identified in this study, such as interferon atmotherapy and reduced metabolic pathway activities. These survival factors may be relevant to improving outcomes among elderly patients with COVID-19. The developed AlgSurv can be applied for clinical risk stratification. Dysregulation of different metabolic pathways revealed in this study suggest potential new therapeutic targets against COVID-19.

## Data Availability Statement

The raw data supporting the conclusions of this article will be made available by the authors, without undue reservation.

## Ethics Statement

This study was approved by the institutional ethics board of Tongji Hospital of Tongji Medical College, Huazhong University of Science and Technology (No. IRBID:TJ-C 20200107). The patients/participants provided their written informed consent to participate in this study.

## Author Contributions

QM, AW, and JL: wrote the manuscript. JL and CK: statistical analysis. YY, ML, FW, and KM: data collection. LW, PW, HC, JLL, SD, and YL: data preparation and analysis. AB, CK, GH, and JL: revised the manuscript. GH, XY, and JL: conceptualization. All authors contributed to the article and approved the submitted version.

## Conflict of Interest

The authors declare that the research was conducted in the absence of any commercial or financial relationships that could be construed as a potential conflict of interest.

## Supplementary Material

Supplementary Figure 1 Estimation of metabolic flux in a large-scale molecular model MCPM.

Supplementary Information 1 Algorithm for utilization of laboratory measurements for the generation of IM.Index.

Supplementary Information 2 Flux difference of 46 pathways within MCPM model.

## References

[1] Zhu N Zhang D Wang W Li X Yang B Song J. A novel coronavirus from patients with pneumonia in China, 2019. N Engl J Med. (2020) 382:727–33. 10.1056/NEJMoa2001017 31978945 PMC7092803

[2] WHO Reports Available online at: https://covid19.who.int/ (accessed October 8, 2020).

[3] Richardson S Hirsch J Narasimhan M Crawford J McGinn T Davidson K. Presenting characteristics, comorbidities, and outcomes among 5700 patients hospitalized with COVID-19 in the New York City area. JAMA. (2020) 323:2052–9. 10.1001/jama.2020.6775 32320003 PMC7177629

[4] Huang C Wang Y Li X Ren L Zhao J Hu Y. Clinical features of patients infected with 2019 novel coronavirus in Wuhan, China. Lancet. (2020) 395:497–506. 10.1016/S0140-6736(20)30183-5 31986264 PMC7159299

[5] Kobayashi KI Kaki T Mizuno S Kubo K Komiya N Otsu S. Clinical characteristics of patients with coronavirus disease 2019 in Japan: a single-center case series. J Infect Dis. (2020) 222:194–7. 10.1093/infdis/jiaa244 32382746 PMC7239124

[6] Cummings MJ Baldwin MR Abrams D Jacobson SD Meyer BJ Balough EM. Epidemiology, clinical course, and outcomes of critically ill adults with COVID-19 in New York city: a prospective cohort study. Lancet. (2020) 395:1763–70. 10.1016/S0140-6736(20)31189-2 32442528 PMC7237188

[7] Guan W Ni Z Hu Y Liang W Ou C He J. Clinical characteristics of coronavirus disease 2019 in China. N Engl J Med. (2020) 382:1708–20. 10.1056/NEJMoa2002032 32109013 PMC7092819

[8] Goyal P Choi J Pinheiro LC Schenck E Chen R Jabri A. Clinical Characteristics of Covid-19 in New York City. N Engl J Med. (2020) 382:2372–4 10.1056/NEJMc2010419 32302078 PMC7182018

[9] Wu Z McGoogan JM. Characteristics of and important lessons from the coronavirus disease 2019 (COVID-19) outbreak in China: summary of a report of 72 314 cases from the chinese center for disease control and prevention. JAMA. (2020) 323:1239–42. 10.1001/jama.2020.2648 32091533

[10] Xu J Kang Y Park SK Yoon Y Bai SJ Jin YD. Nasality changes with age in normal korean-speaking adults. Clin Exp Otorhinolaryngol. (2019) 12:95–9. 10.21053/ceo.2018.00717 30257547 PMC6315219

[11] Aw D Silva AB Palmer DB. Immunosenescence: emerging challenges for an ageing population. Immunology. (2007) 120:435–46. 10.1111/j.1365-2567.2007.02555.x 17313487 PMC2265901

[12] Xia S Zhang X Zheng S Khanabdali R Kalionis B Wu J. An update on inflamm-aging: mechanisms, prevention, and treatment. J Immunol Res. (2016) 2016:8426874. 10.1155/2016/8426874 27493973 PMC4963991

[13] Kelly PN Metabolism as a driver of immune response. Science. (2019) 363:137–9. 10.1126/science.363.6423.137-j

[14] Maasilta P Holsti LR Halme M Kivisaari L Cantell K Mattson K. Natural alpha-interferon in combination with hyperfractionated radiotherapy in the treatment of non-small cell lung cancer. Int J Radiat Oncol. (1992) 23:863–8. 10.1016/0360-3016(92)90660-a 1319982

[15] Chen L Shi M Deng Q Liu W Li Q Ye P A multi-center randomized prospective study on the treatment of infant bronchiolitis with interferon α1b nebulization. PLoS ONE. (2020) 15:e0228391 10.1371/journal.pone.0228391 32084142 PMC7034796

[16] Phelan AL Katz R Gostin LO. The novel coronavirus originating in Wuhan, China: challenges for global health governance. JAMA. (2020) 323:709–10. 10.1001/jama.2020.1097 31999307

[17] Zhang M Saad C Le L Halfter K Bauer B Mansmann U. Computational modeling of methionine cycle-based metabolism and DNA methylation and the implications for anti-cancer drug response prediction. Oncotarget. (2018) 9:22546–58. 10.18632/oncotarget.24547 29875994 PMC5989406

[18] Vasquez MM Hu C Roe DJ Chen Z Halonen M Guerra S. Least absolute shrinkage and selection operator type methods for the identification of serum biomarkers of overweight and obesity: simulation and application. BMC Med Res Methodol. (2016) 16:154. 10.1186/s12874-016-0254-8 27842498 PMC5109787

[19] Wu Q Zhou L Sun X Yan Z Hu C Wu J. Altered lipid metabolism in recovered SARS patients twelve years after infection. Sci Rep. (2017) 7:9110. 10.1038/s41598-017-09536-z 28831119 PMC5567209

[20] Zheng YY Ma YT Zhang JY Xie X. COVID-19 and the cardiovascular system. Nat Rev Cardiol. (2020) 17:259–60. 10.1038/s41569-020-0360-5 32139904 PMC7095524

[21] Lunt SY Vander Heiden MG. Aerobic glycolysis: meeting the metabolic requirements of cell proliferation. Annu Rev Cell Dev Biol. (2011) 27:441–64. 10.1146/annurev-cellbio-092910-154237 21985671

[22] Huber K Hofer DC Trefely S Pelzmann H Maddreiter-Sokolowski C Duta-Mare M. N-acetylaspartate pathway is nutrient responsive and coordinates lipid and energy metabolism in brown adipocytes. Biochim Biophys Acta Mol Cell Res. (2019) 1866:337–48. 10.1016/j.bbamcr.2018.08.017 30595160 PMC6390944

[23] Lane AN Fan TWM Regulation of mammalian nucleotide metabolism and biosynthesis. Nucleic Acids Res. (2015) 43:2466–85. 10.1093/nar/gkv047 25628363 PMC4344498

[24] Cui J Li F Shi ZL. Origin and evolution of pathogenic coronaviruses. Nat Rev Microbiol. (2019) 17:181–92. 10.1038/s41579-018-0118-9 30531947 PMC7097006

[25] Gray LR Tompkins SC Taylor EB. Regulation of pyruvate metabolism and human disease. Cell Mol Life Sci. (2014) 71:2577–604. 10.1007/s00018-013-1539-2 24363178 PMC4059968

[26] Sun X Song L Feng S Li L Yu H Wang Q. Fatty acid metabolism is associated with disease severity after H7N9 infection. EBioMedicine. (2018) 33:218–29. 10.1016/j.ebiom.2018.06.019 29941340 PMC6085509

[27] Linden J Koch-Nolte F Dahl G. Purine release, metabolism, and signaling in the inflammatory response. Annu Rev Immunol. (2019) 37:325–47. 10.1146/annurev-immunol-051116-052406 30676821

[28] Shaikh FY Cox RG Lifland AW Hotard AL Williams JV Moore ML. A critical phenylalanine residue in the respiratory syncytial virus fusion protein cytoplasmic tail mediates assembly of internal viral proteins into viral filaments and particles. MBio. (2012) 3:e00270-11. 10.1128/mBio.00270-11 22318318 PMC3280462

[29] Buck MD O'Sullivan D Pearce EL. T cell metabolism drives immunityT cell metabolism drives immunity. J Exp Med. (2015) 212:1345–60. 10.1084/jem.20151159 26261266 PMC4548052

[30] Blagih J Coulombe F Vincent E Dupuy F Galicia-Vazquez G Yurchenko E. The energy sensor AMPK regulates T cell metabolic adaptation and effector responses *in vivo*. Immunity. (2015) 42:41–54. 10.1016/j.immuni.2014.12.030 25607458

[31] Zhang Q Bastard P Liu Z Le Pen J Moncada-Velez M Chen J Inborn errors of type I IFN immunity in patients with life-threatening COVID-19. Science. (2020) 370:abd4570 10.1126/science.abd4570 PMC7857407 32972995

[32] van der Made CI Simons A Schuurs-Hoejimakers J van den Heuvel G Mantere T Kersten S. Presence of genetic variants among young men with severe COVID-19. JAMA. (2020) 324:663–73 10.1001/jama.2020.13719 32706371 PMC7382021

[33] Hung IFN Lung KC Tso E Liu R Chung T Chu MY. Triple combination of interferon beta-1b, lopinavir–ritonavir, and ribavirin in the treatment of patients admitted to hospital with COVID-19: an open-label, randomised, phase 2 trial. Lancet. (2020) 395:1695–704. 10.1016/S0140-6736(20)31042-4 32401715 PMC7211500

[34] Zhou Q Chen V Shannon CP Wei XS Xiang X Wang X Interferon-α2b treatment for COVID-19. Front Immunol. (2020) 11:1061 10.3389/fimmun.2020.01061 32574262 PMC7242746

[35] Wang D Hu B Hu C Zhu F Liu X Zhang J. Clinical characteristics of 138 hospitalized patients with 2019 novel coronavirus-infected pneumonia in Wuhan, China. JAMA. (2020) 323:1061–9. 10.1001/jama.2020.1585 32031570 PMC7042881

[36] Ruan Q Yang K Wang W Jiang L Song J Clinical predictors of mortality due to COVID-19 based on an analysis of data of 150 patients from Wuhan, China. Intensive Care Med. (2020) 46:846–8. 10.1007/s00134-020-05991-x 32125452 PMC7080116

[37] Zhou F Yu T Du R Fan G Liu Y Liu Z Clinical course and risk factors for mortality of adult inpatients with COVID-19 in Wuhan, China: a retrospective cohort study. Lancet. (2020) 395:1054–62. 10.1016/S0140-6736(20)30565-3 32171076 PMC7270627

[38] Glassock RJ Warnock DG Delanaye P. The global burden of chronic kidney disease: estimates, variability and pitfalls. Nat Rev Nephrol. (2017) 13:104–14. 10.1038/nrneph.2016.163 27941934

[39] Zheng Y Ley SH Hu FB. Global aetiology and epidemiology of type 2 diabetes mellitus and its complications. Nat Rev Endocrinol. (2018) 14:88–98. 10.1038/nrendo.2017.151 29219149

[40] Reiffel JA Propensity-score matching: optimal, adequate, or incomplete? J Atr Fibrillation. (2018) 11:2130 10.4022/jafib.2130 31139292 PMC6533842

