# Supplementary figures and images for: Survival Factors and Metabolic Pathogenesis in Elderly Patients (≥65) With COVID-19: A Multi-Center Study
Source: Front Med (Lausanne). 2021 Jan 7;7:595503. doi: 10.3389/fmed.2020.595503 (PMC7873923; doi:10.3389/fmed.2020.595503)

# Supplement Figure 1

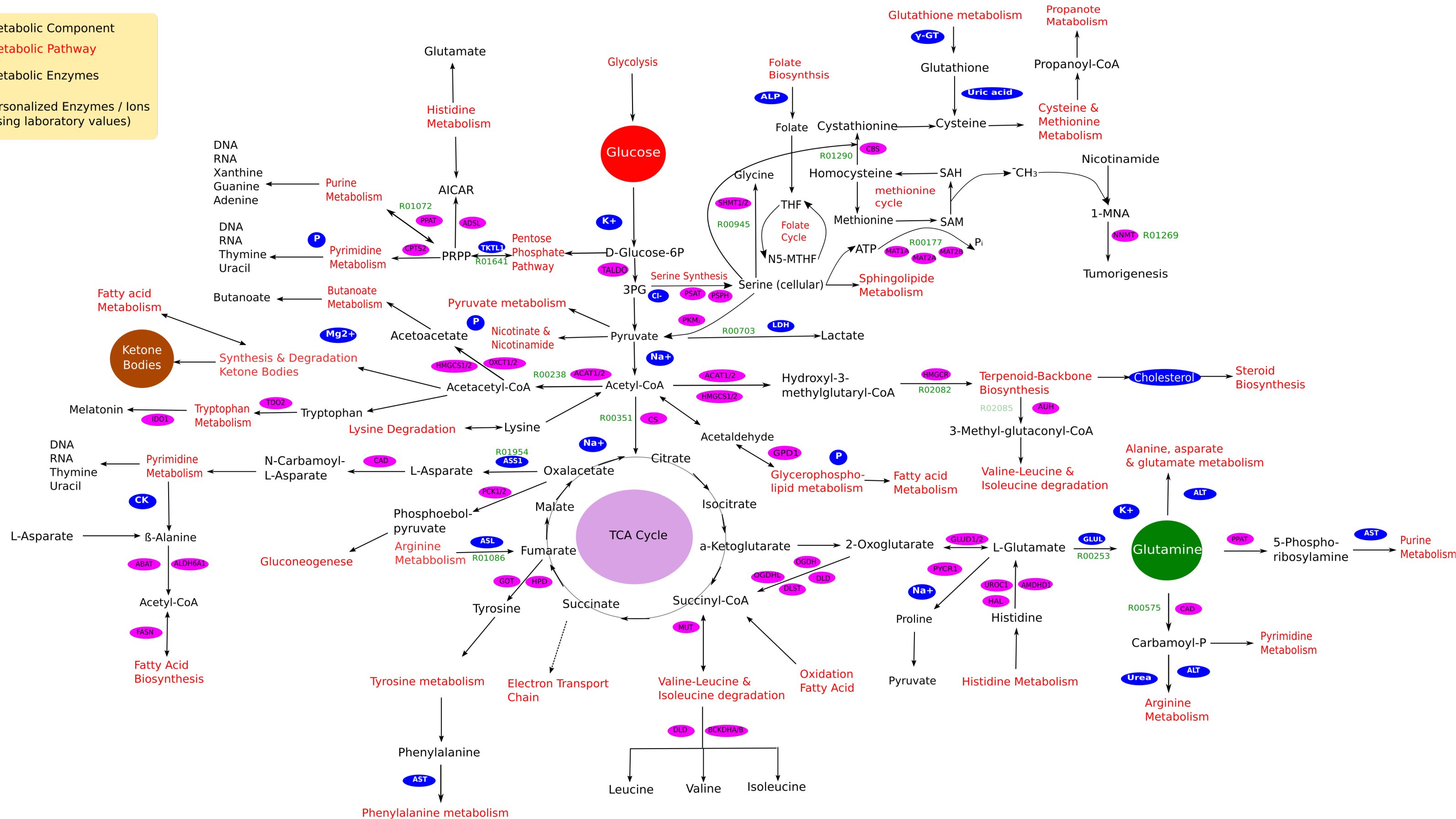

Supplement: Supplementary Figure 1 — Estimation of metabolic flux in a large-scale molecular model MCPM. [file Data_Sheet_1.PDF]
