# Supplementary material for: Survival Factors and Metabolic Pathogenesis in Elderly Patients (≥65) With COVID-19: A Multi-Center Study
Source: Front Med (Lausanne). 2021 Jan 7;7:595503. doi: 10.3389/fmed.2020.595503 (PMC7873923; doi:10.3389/fmed.2020.595503)
Supplement: Supplementary Information 1 — Algorithm for utilization of laboratory measurements for the generation of IM.Index. [file Data_Sheet_2.PDF]

Supplement Information 1. Algorithm for utilization of laboratory measurements for the generation of IM.Index

The MCPM is a molecular metabolic model containing 54 pathways that consists of 1314 gene, 2654 RNA, 1374 protein, 993 compound and 67 complex. The structure of MCPM can be therefore divided into four level: gene --> RNA --> protein / complex --> pathway (Supplement Figure 1; Supplement_model.xml). The assumptions for this application are a.) cellular environmental factors including water, oxygen, carbon dioxide, phosphate, AMP, GMP, and ion such as H+ are sufficiently available; b.) after viral invasion, the viral replication requires a large amount of energy and nutrient from the host cell, therefore the metabolism remodelling occurred and all metabolic genes become active.

The aim of this algorithm is to utilize the information provided by the laboratory data and generate differentiable flux flowing through the entire metabolic network. Diverse metabolic interactions are taking place either in a concurrent manner or one after another to influence the intensity and direction of flux flow. At the end, the IM.Index summarizes this flux in this model at a system level. We hypothesize that IM.Index may approximately reflect overall metabolic activity.

At first, the model file with XML format is input into a digital variable (M). All reactions in M are sorted into three categories to cope with the aforementioned structure level of the model: [1] transcriptions (TAs), [2] translations (TLs), [3] protein-protein interaction and metabolic reaction (PPs). Iteration through TAs to activate all transcription reaction and calculate the fluxes, afterwards the fluxes of TAs flowing to the TLs. While the fluxes of TLs flow into the PPs, the laboratory measurements are assigned to the corresponding components taking part in the different reactions in PPs. In this way, the intensities of flux can be influenced by the inputted laboratory values. After all reactions in the model possess their fluxes, the flux of each pathway can be summarized by influx (flux flowing into a pathway) and exflux (flux flowing out from a pathway). Flux comparison analysis has been performed to investigate whether pathway fluxes could be significantly influenced by the inputted laboratory data using two different states: control (without laboratory data) VS. active (with laboratory data). The results showed that out of 54 pathways, 46 pathways possess significant intensities of flux compared to that of pathways in the control state (Supplement Information 2).

The detailed algorithm:

For each patient:

1. M = read input file (Supplement_model.xml)

    1.1 iterate all components in the M and assign corresponding components with laboratory data.

    1.2 sort(M) => three arrays: TAs, TLs, and PPs

2. Calculation flux of reactions:

$$I_j \text{ (reaction, role) } = \prod_{c(object)} \text{ objects} \in \text{reactant (reaction,role).objects} \quad (1)$$

For i = 1 to number (TAs):

    2.1 calculate flux of each reaction in TAs using (1)

For j = 1 to number (TLs):

    2.2 calculate flux of each reaction in TLs using (1)

For k = 1 to number (PPs):

    2.3 calculate flux of each reaction in PPs using (1)

3. Calculation flux of pathways:

For n = 1 to number (pathways):

$$3.1 \text{ flux(P)} = \left(\sum_{i \in P} I_i\text{(reaction, role)} / \text{ N(P)} \right) - \text{flux(Crosstalk(P))} \quad (2)$$

4. Calculation of IM.Index of a patient:

$$4.1 \text{ IM.Index [patient]} = \sum \text{flux(P) / N(P)} \quad (3)$$
