# Supplementary material for: Survival Factors and Metabolic Pathogenesis in Elderly Patients (≥65) With COVID-19: A Multi-Center Study
Source: Front Med (Lausanne). 2021 Jan 7;7:595503. doi: 10.3389/fmed.2020.595503 (PMC7873923; doi:10.3389/fmed.2020.595503)
Supplement: Supplementary Information 2 — Flux difference of 46 pathways within MCPM model. [file Data_Sheet_3.PDF]

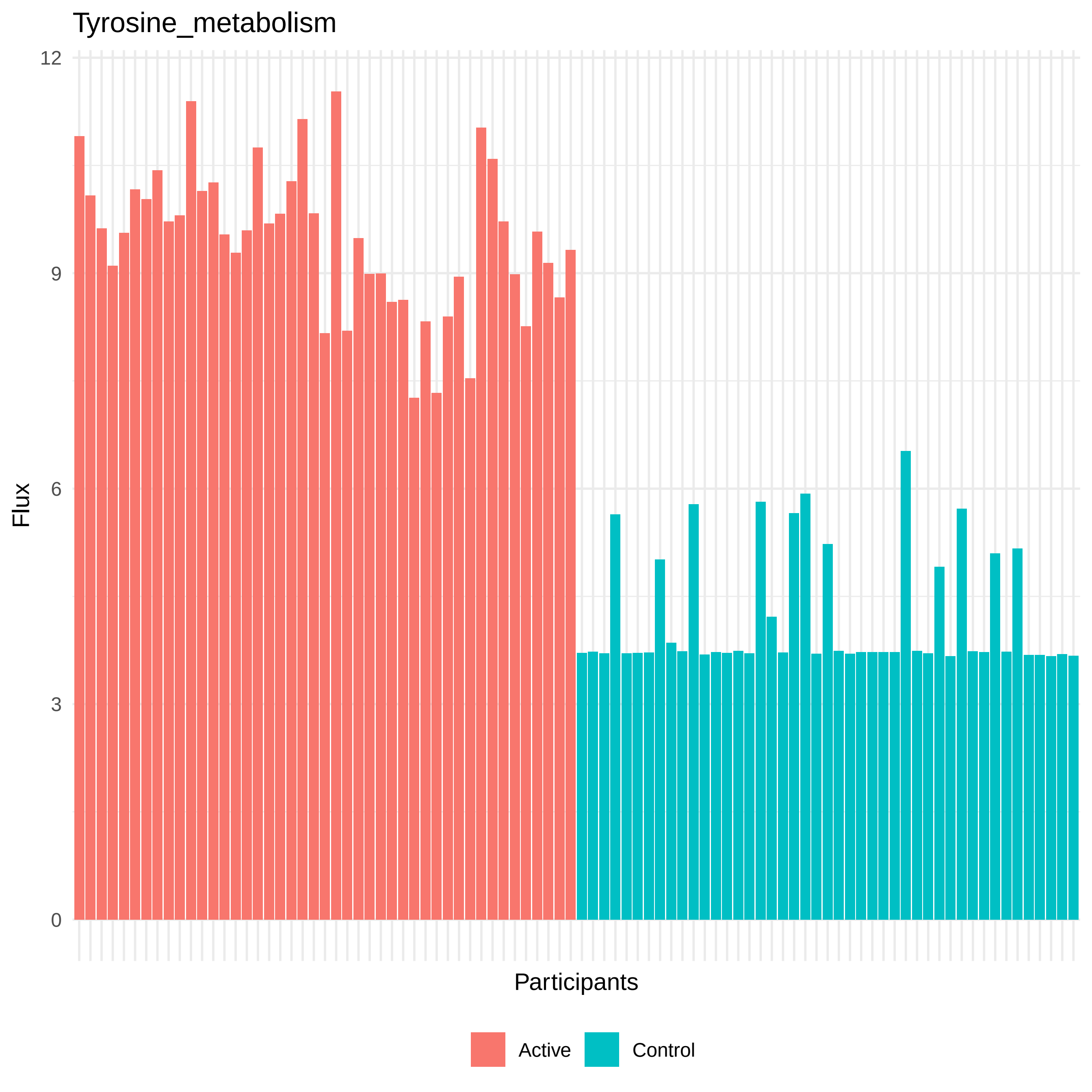

Tyrosine_metabolism
12
9
6
3
0
Flux
Participants
Active
Control

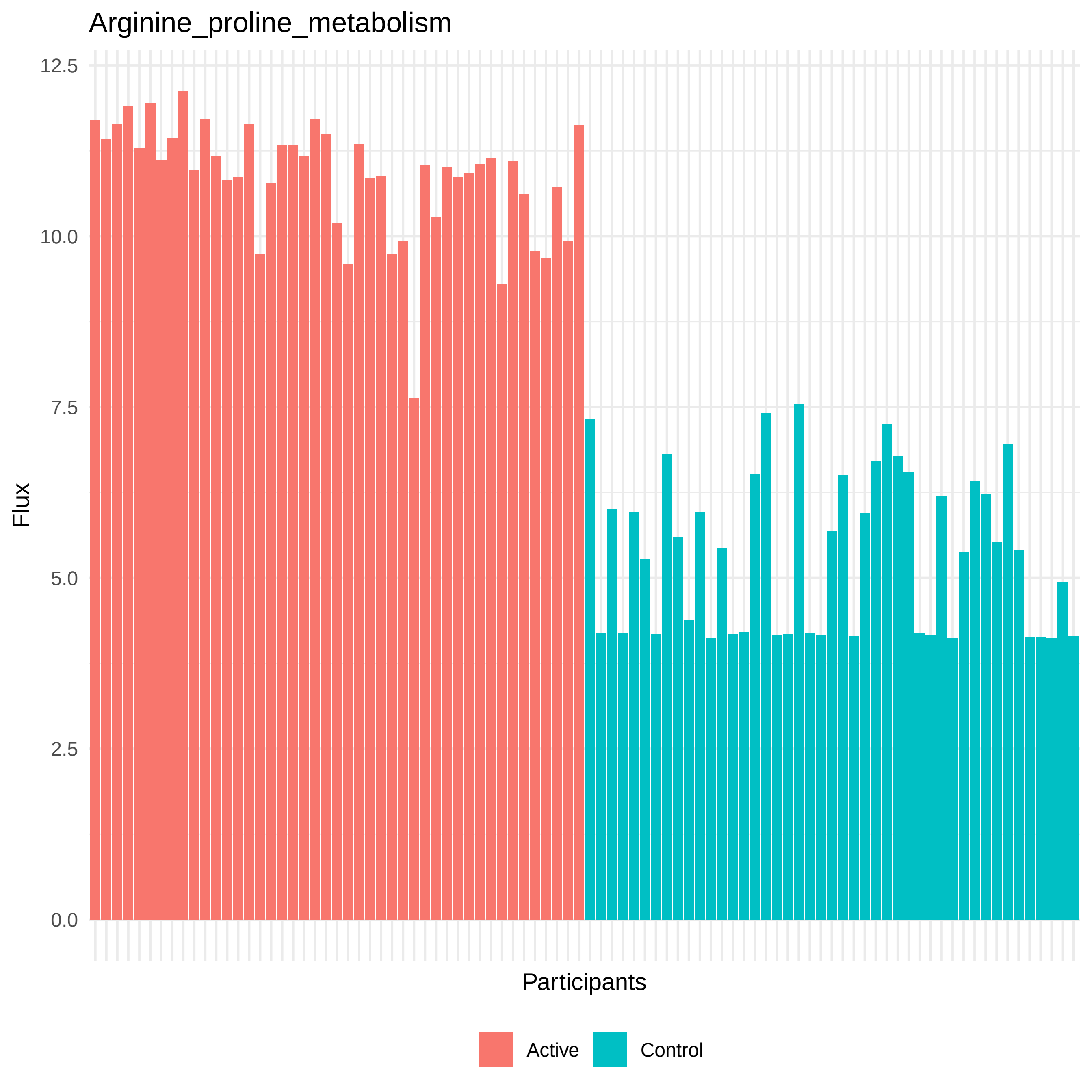
Arginine_proline_metabolism
Flux
12.5
10.0
7.5
5.0
2.5
0.0
Participants
Active
Control

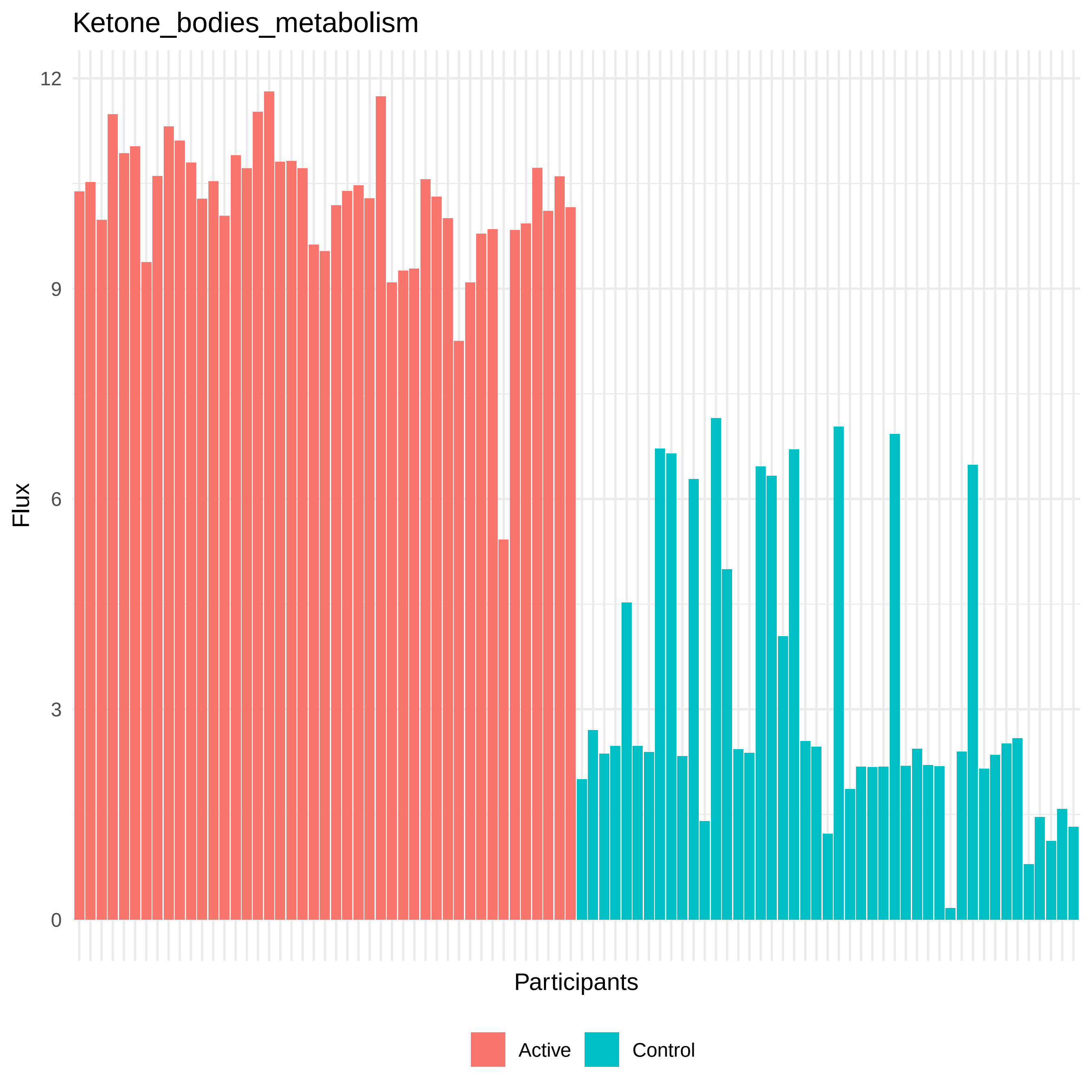
Ketone_bodies_metabolism
12
9
6
3
0
Flux
Participants
Active
Control

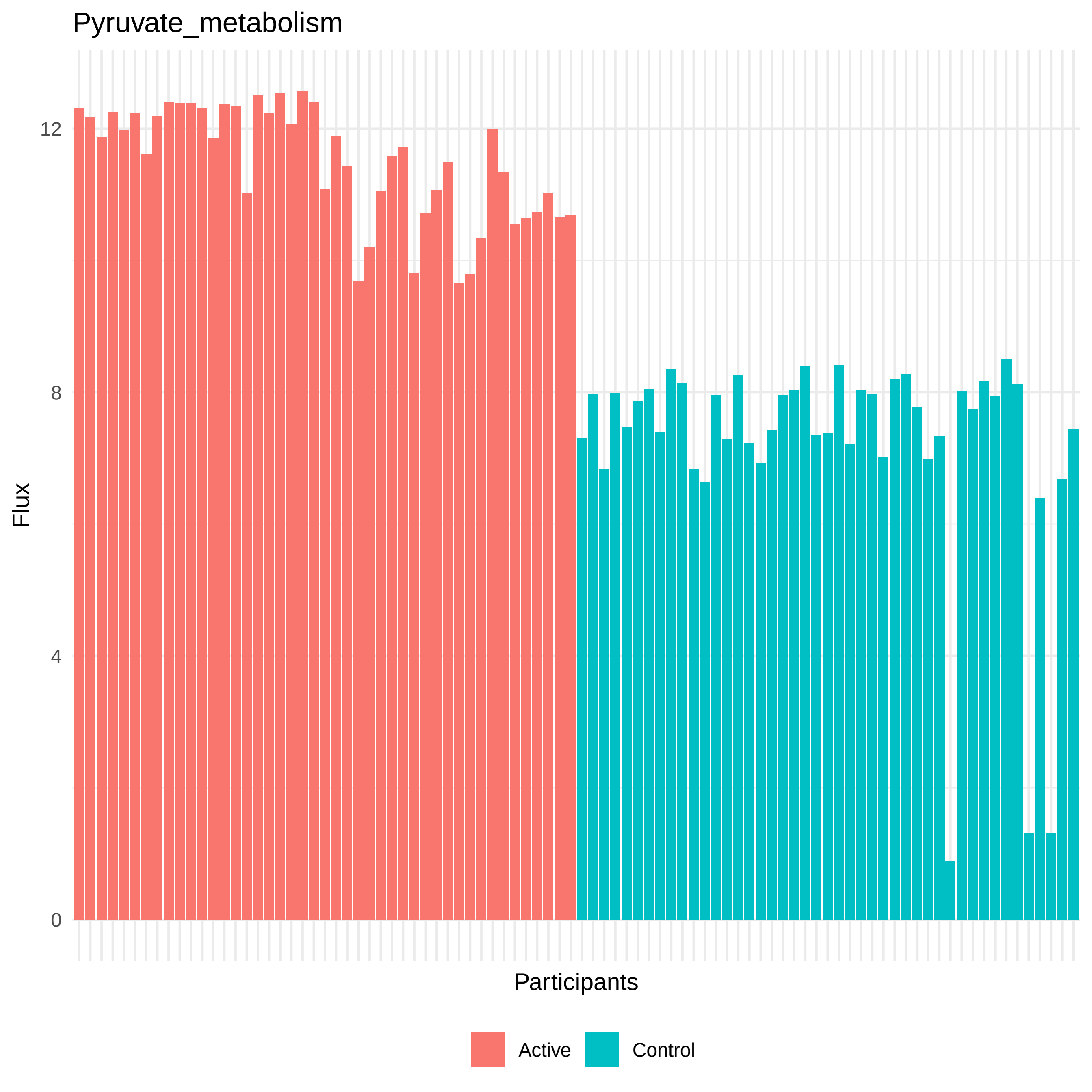

Pyruvate_metabolism
12
8
4
0
Flux
Participants
Active
Control

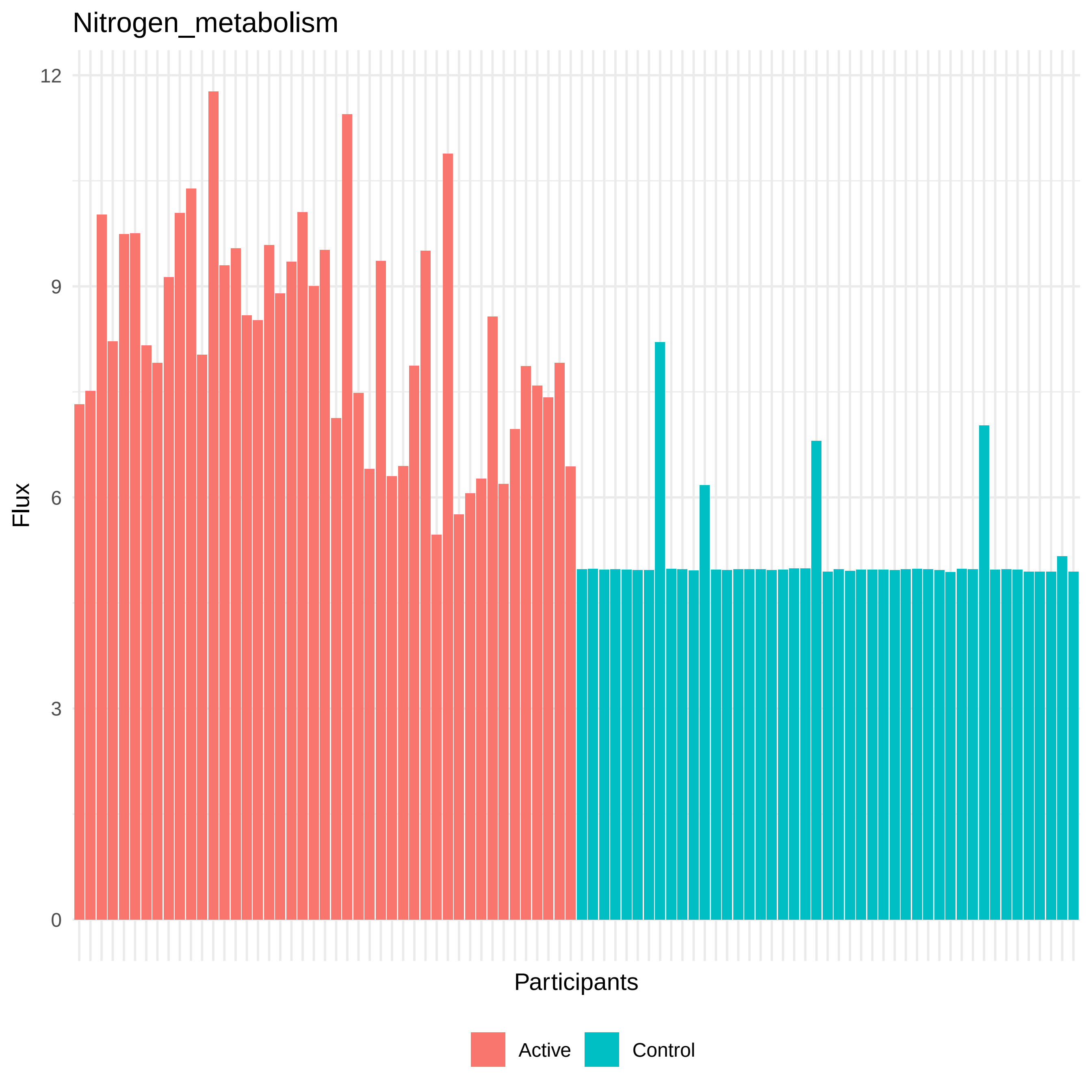

Nitrogen_metabolism
12
9
6
3
0
Flux
Participants
Active
Control

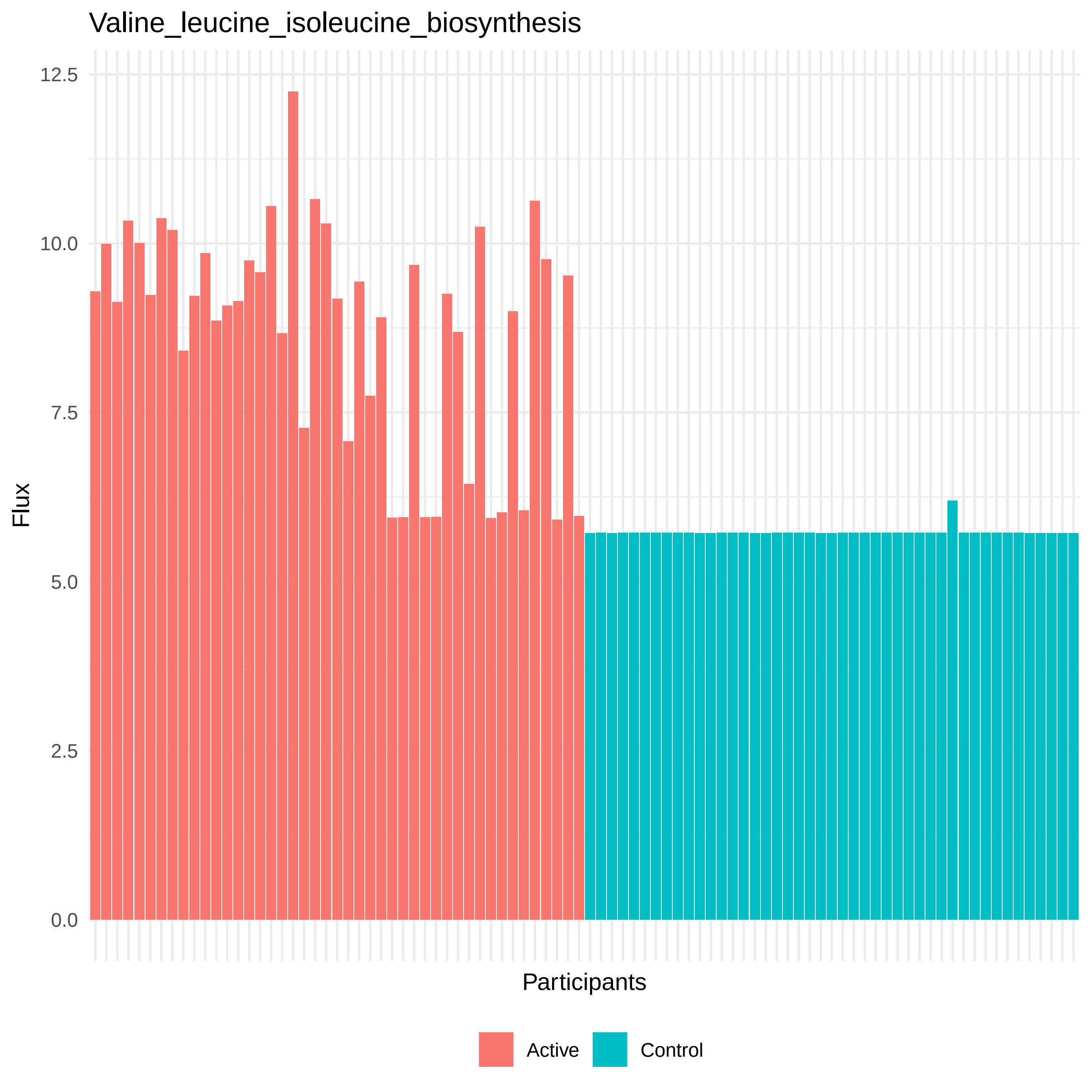

Valine_leucine_isoleucine_biosynthesis
12.5
10.0
7.5
5.0
2.5
0.0
Flux
Participants
Active
Control

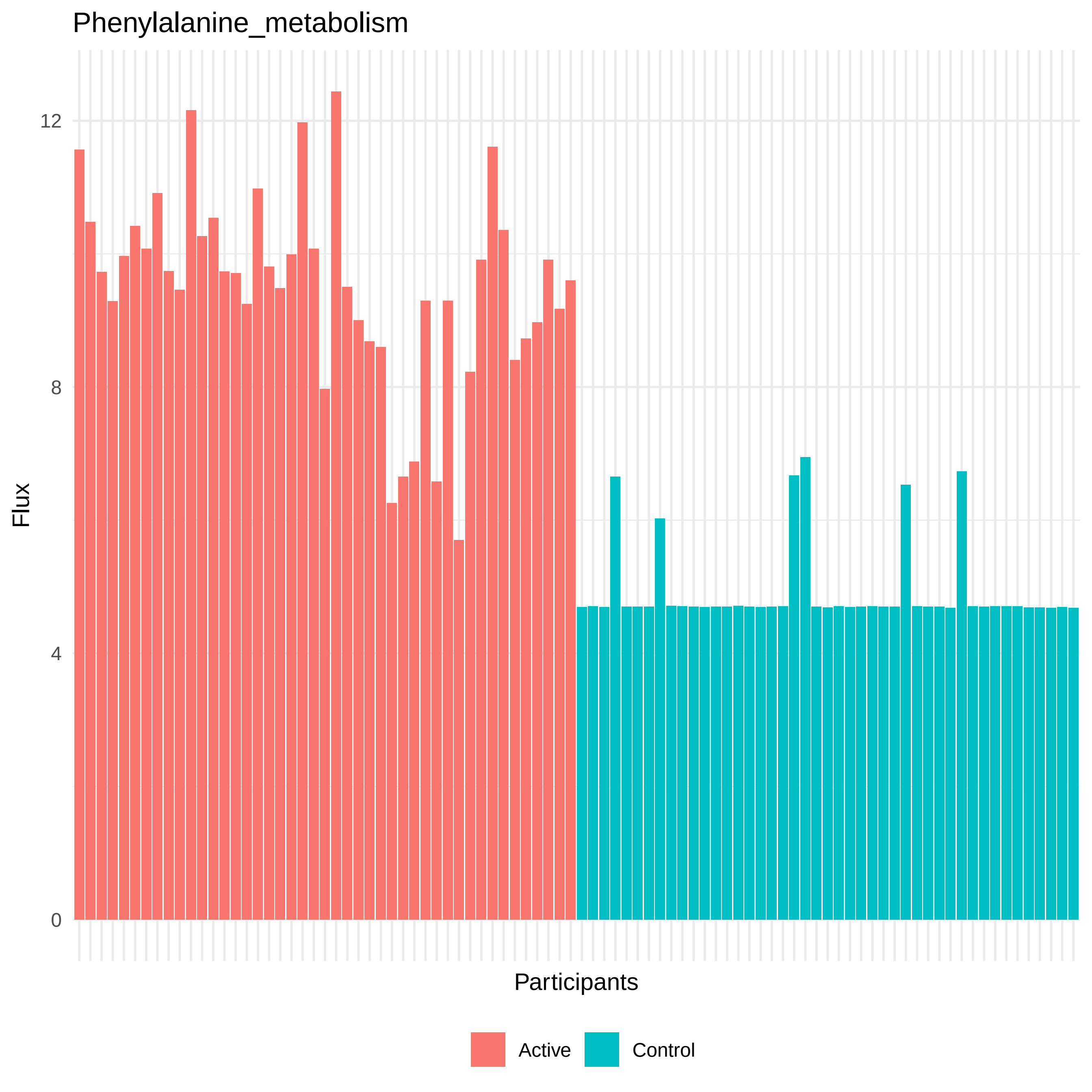

Phenylalanine_metabolism
Flux
12
8
4
0
Participants
Active
Control

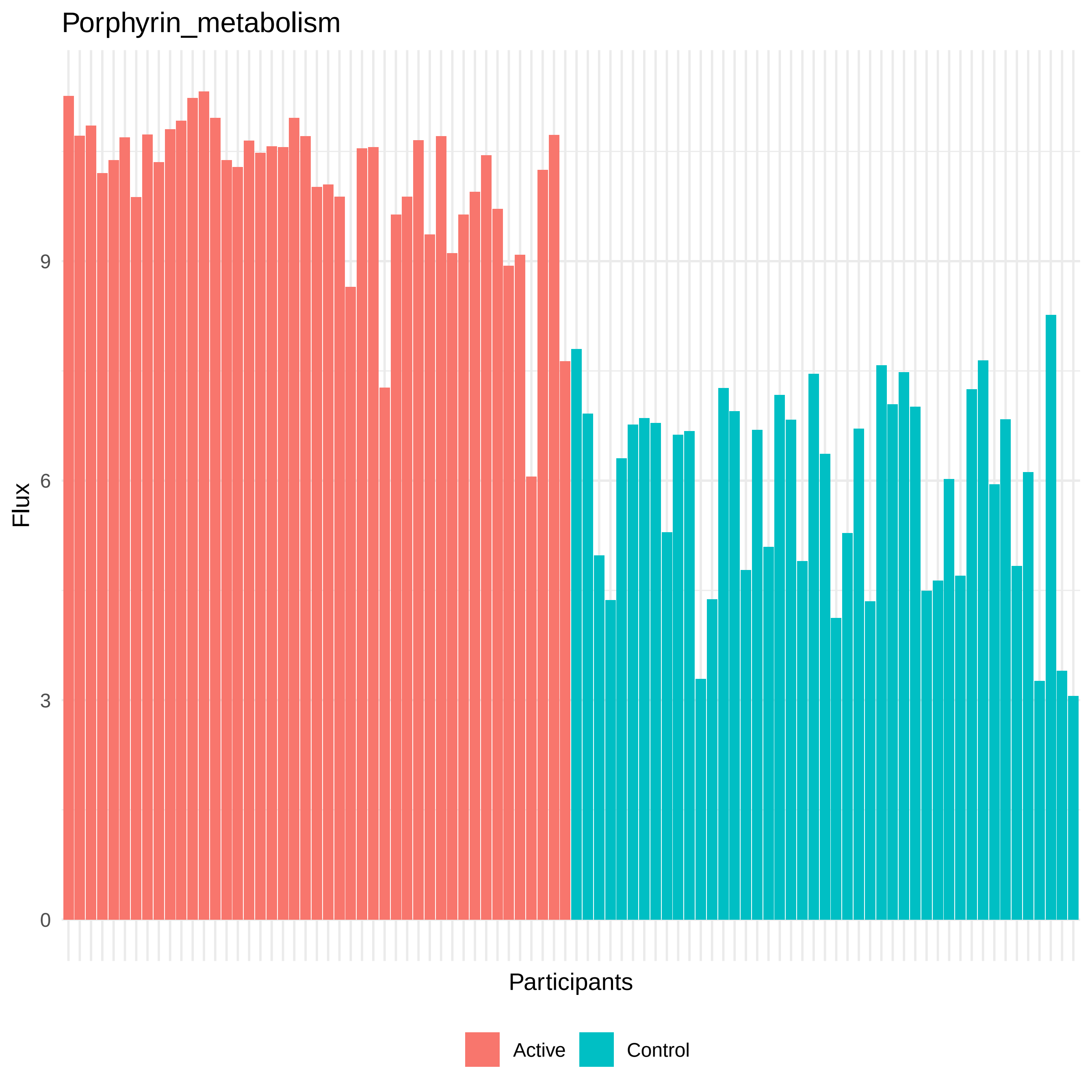
Porphyrin_metabolism
Flux
9
6
3
0
Participants
Active
Control

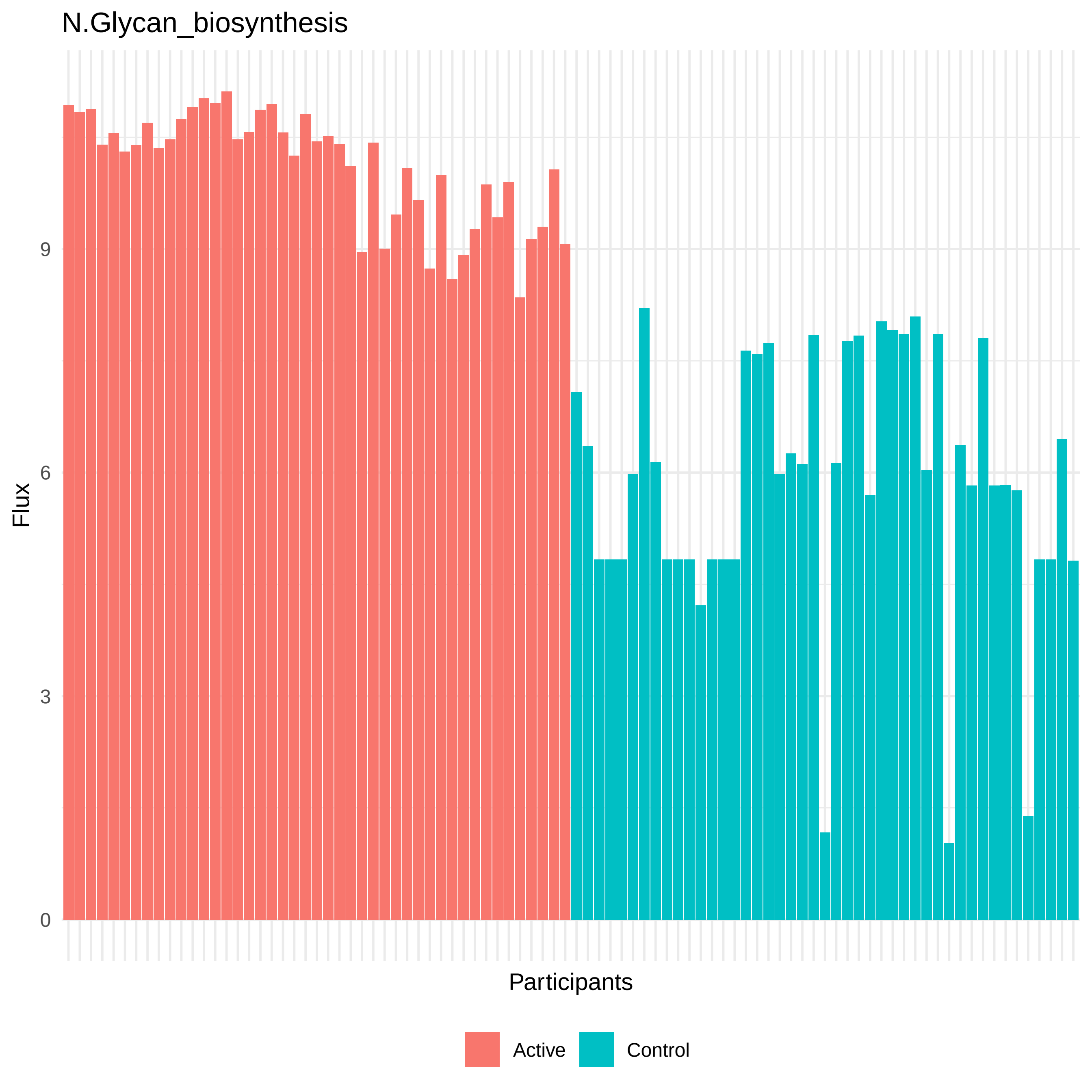

N.Glycan_biosynthesis
Flux
9
6
3
0
Participants
Active
Control

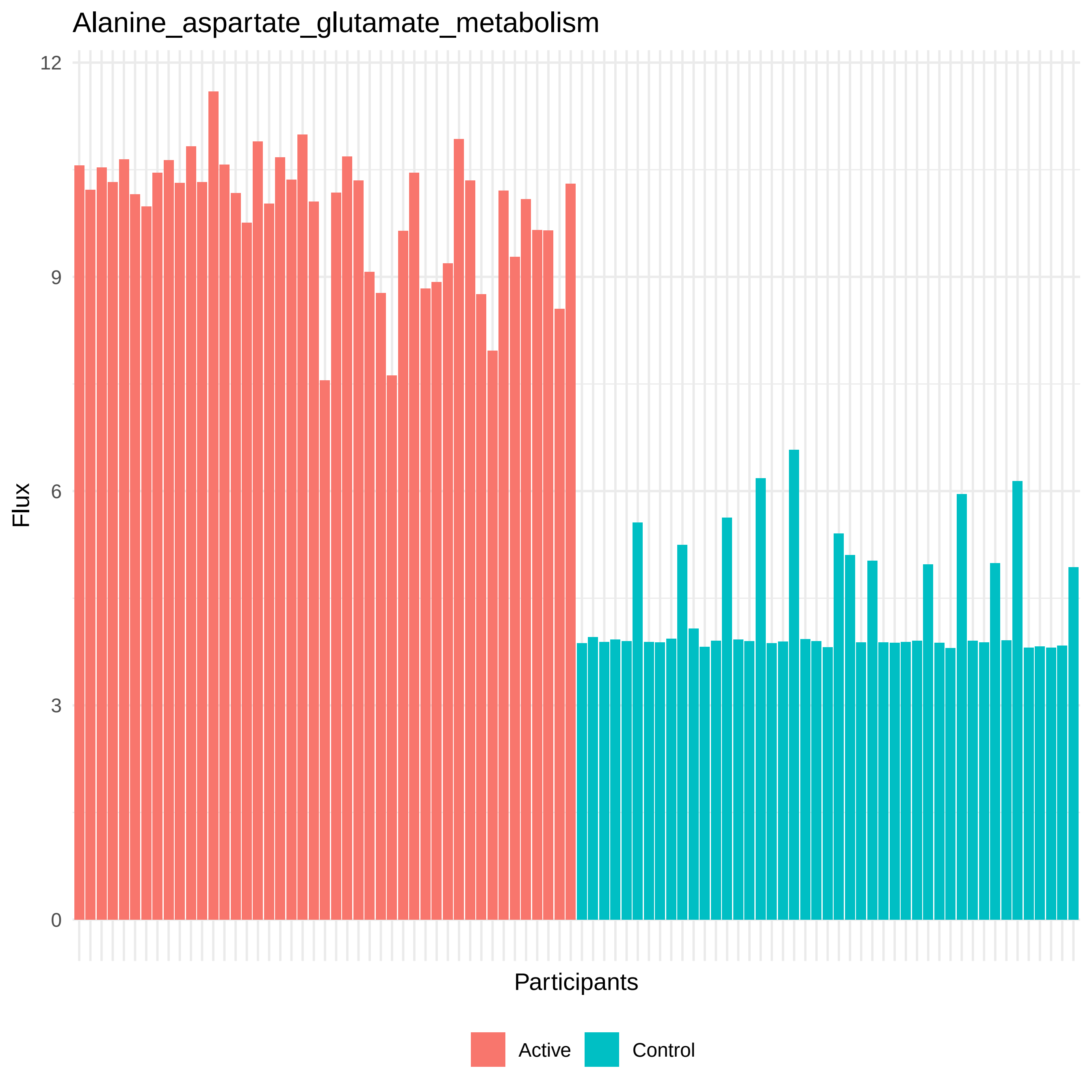
Alanine_aspartate_glutamate_metabolism
12
9
6
3
0
Flux
Participants
Active
Control

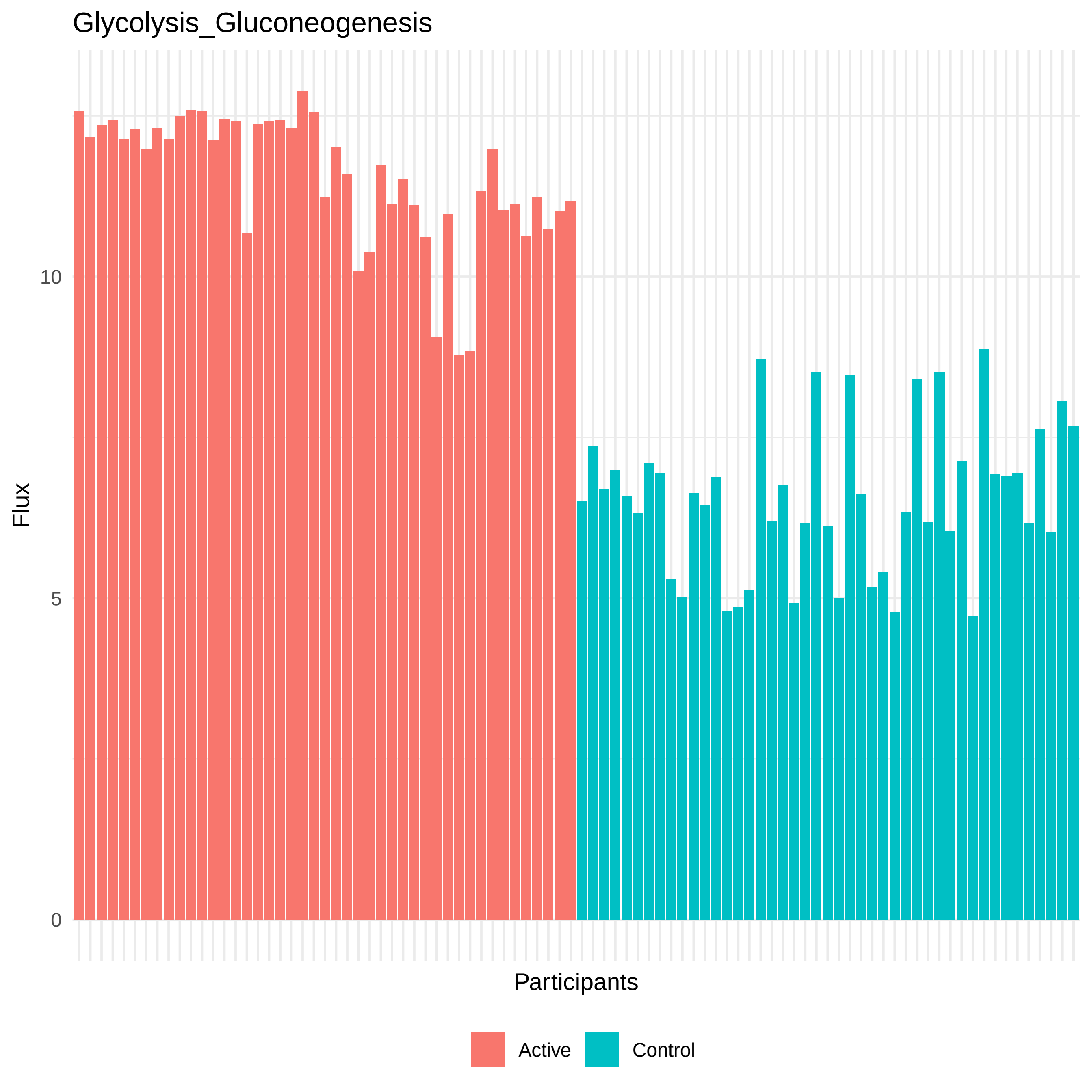
Glycolysis_Gluconeogenesis
Flux
10
5
0
Participants
Active
Control

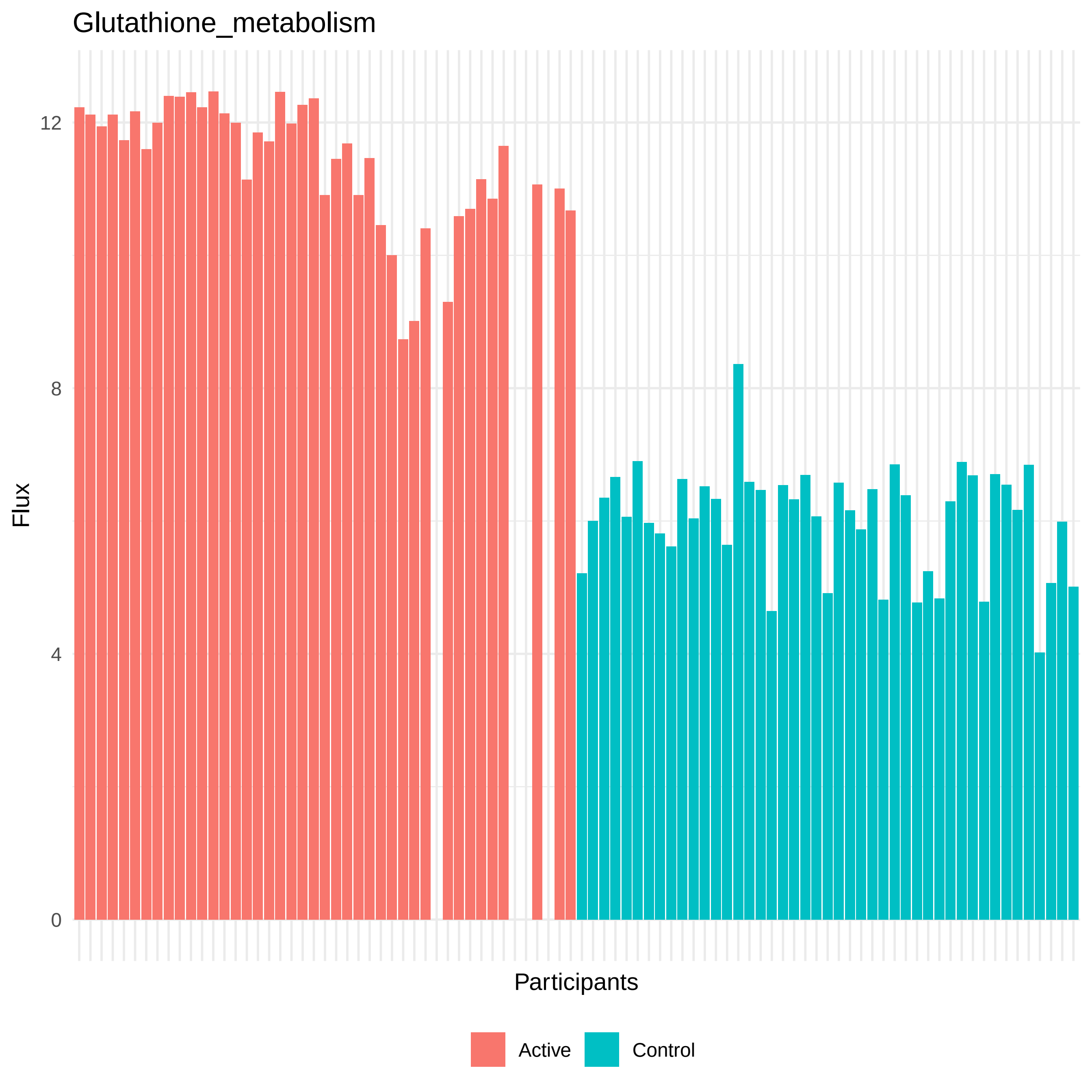
Glutathione_metabolism
12
8
4
0
Flux
Participants
Active
Control

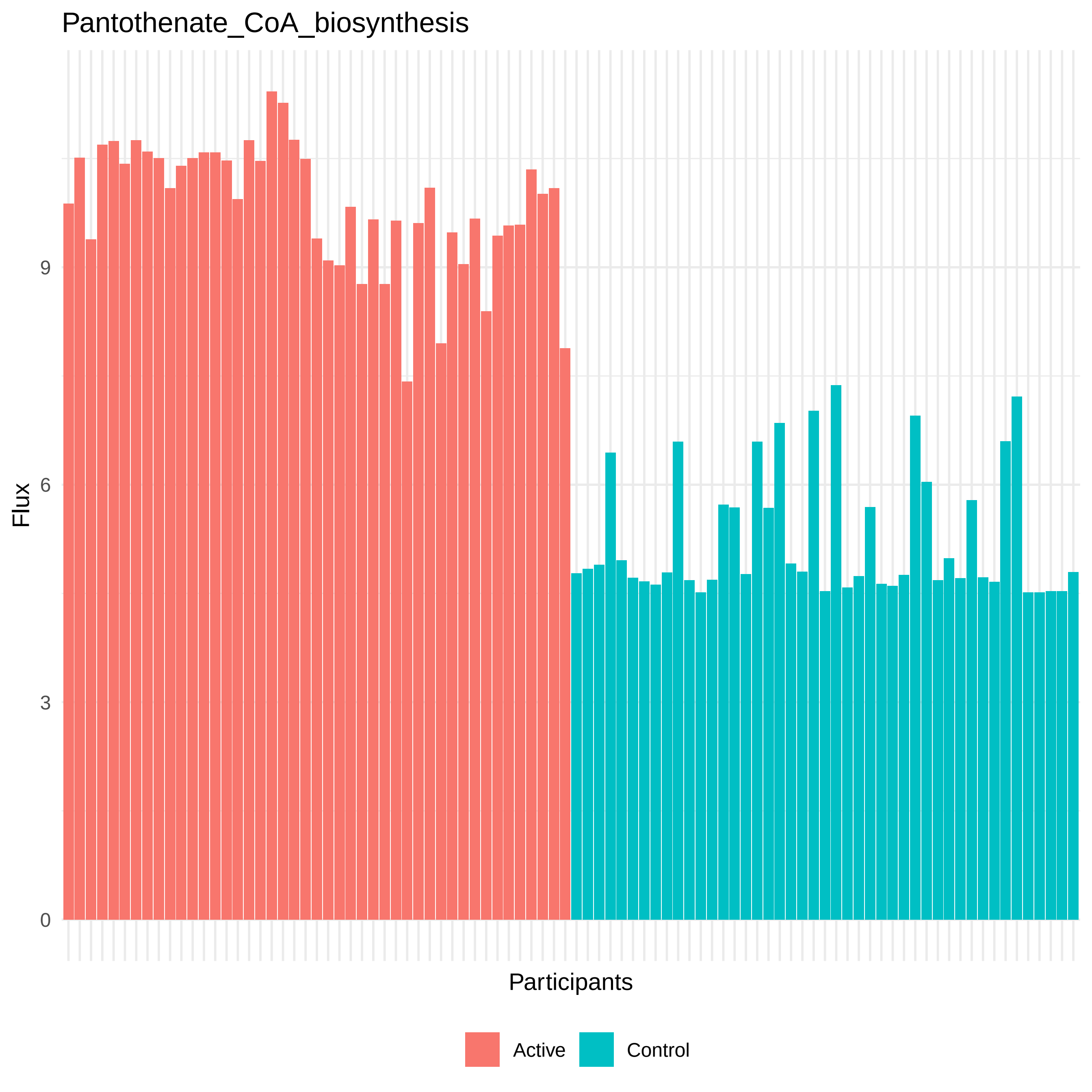

Pantothenate_CoA_biosynthesis
Flux
9
6
3
0
Participants
Active
Control

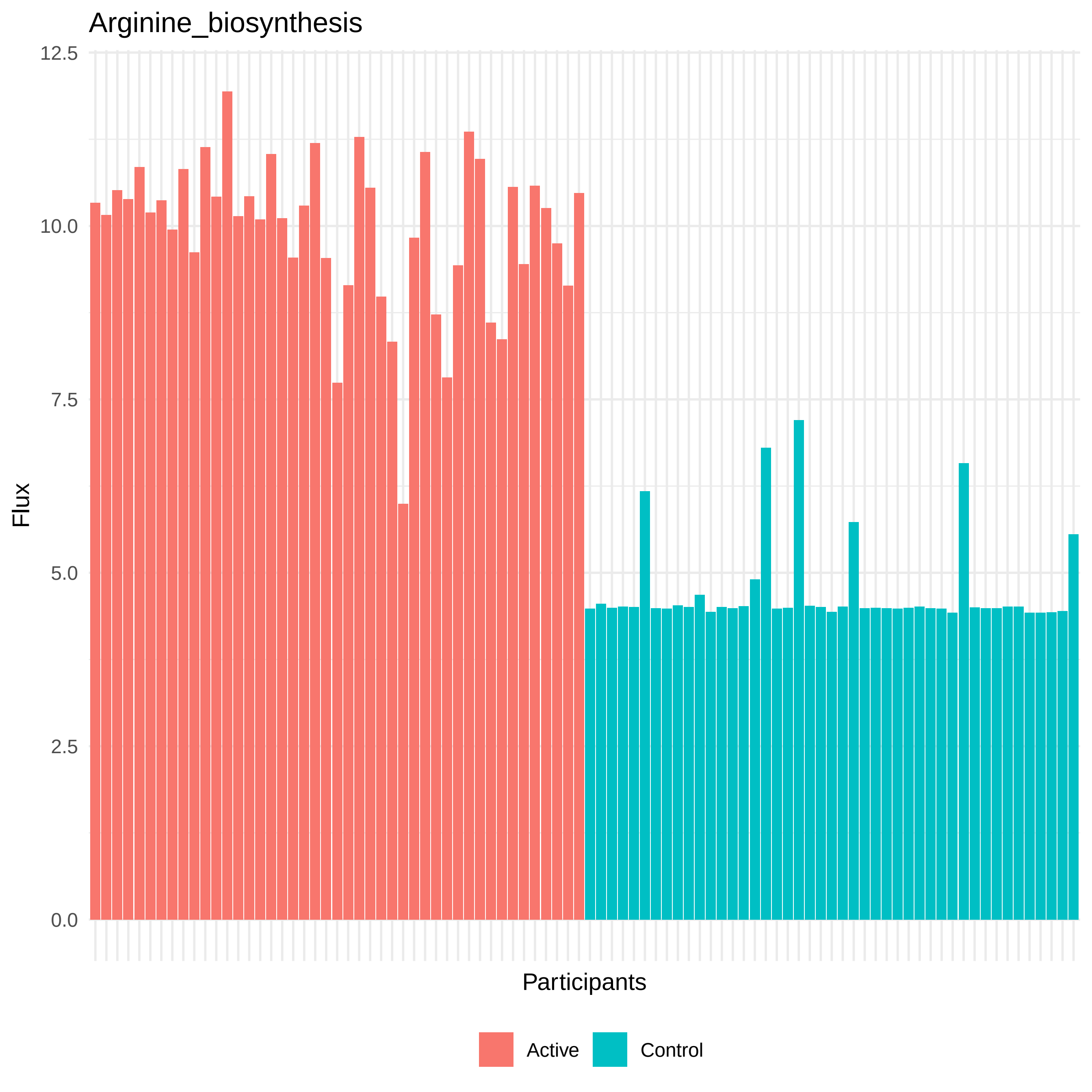
Arginine_biosynthesis
Flux
12.5
10.0
7.5
5.0
2.5
0.0
Participants
Active
Control

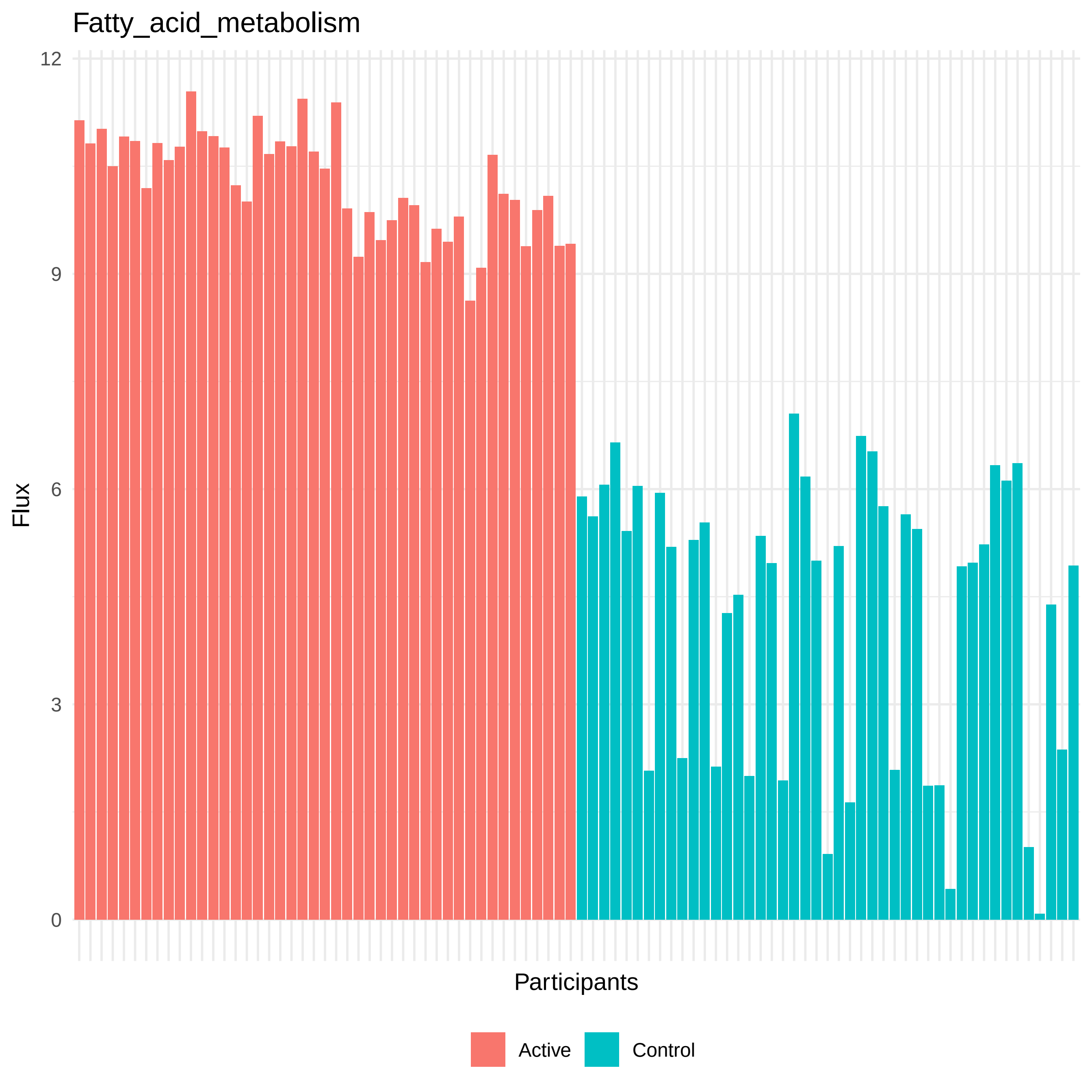

Fatty_acid_metabolism
Flux
12
9
6
3
0
Participants
Active
Control

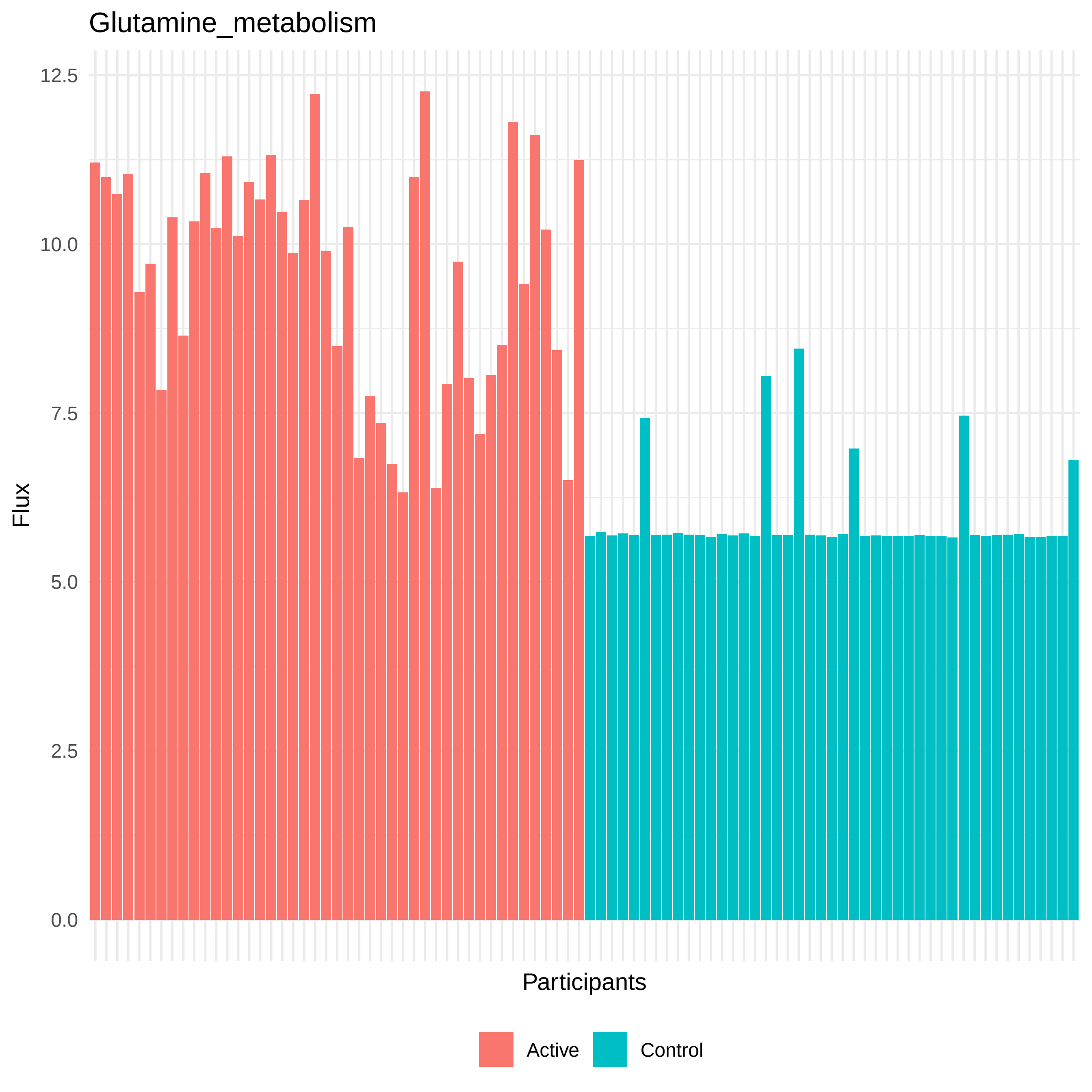
Glutamine_metabolism
12.5
10.0
7.5
5.0
2.5
0.0
Flux
Participants
Active
Control

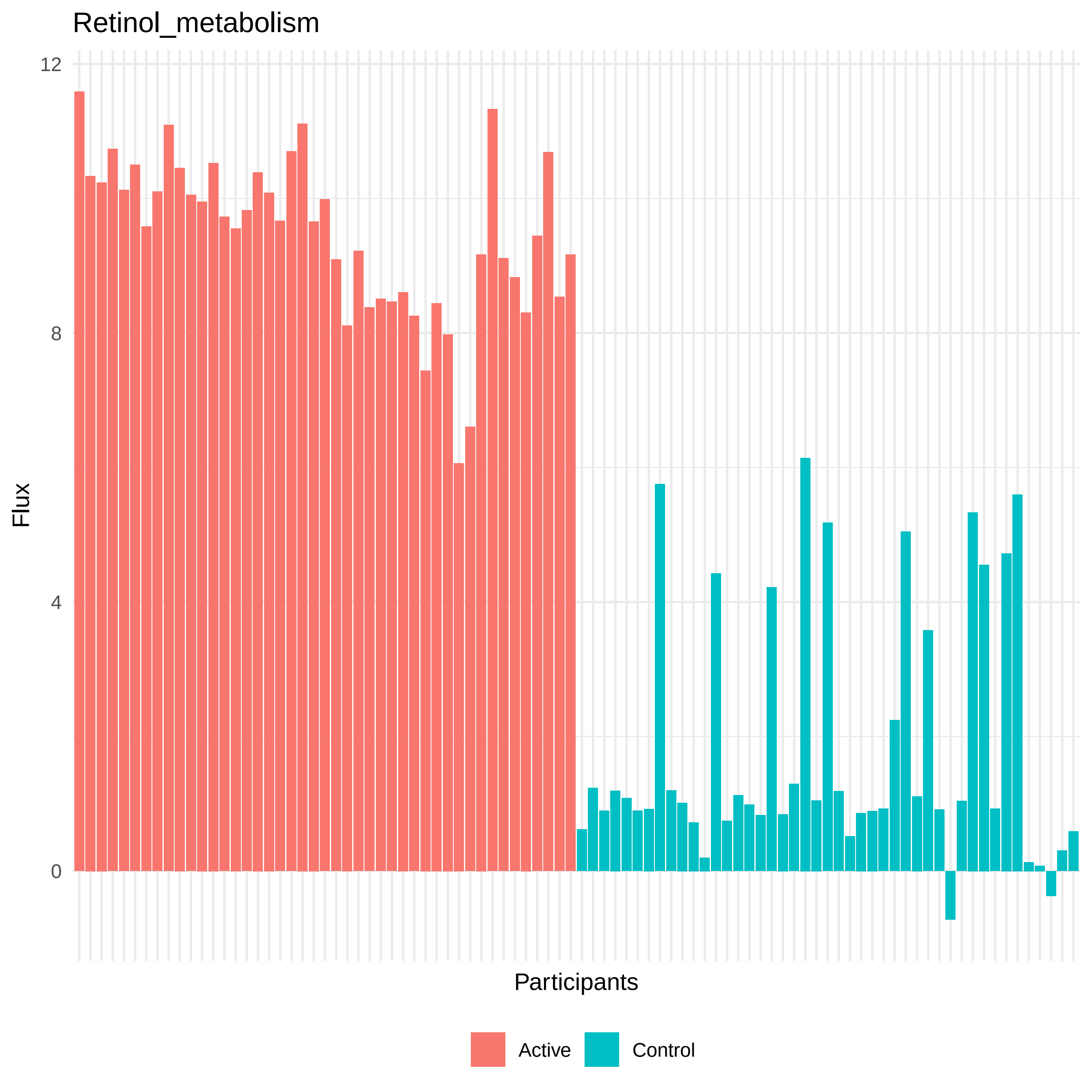

Retinol_metabolism
12
8
4
0
Flux
Participants
Active
Control

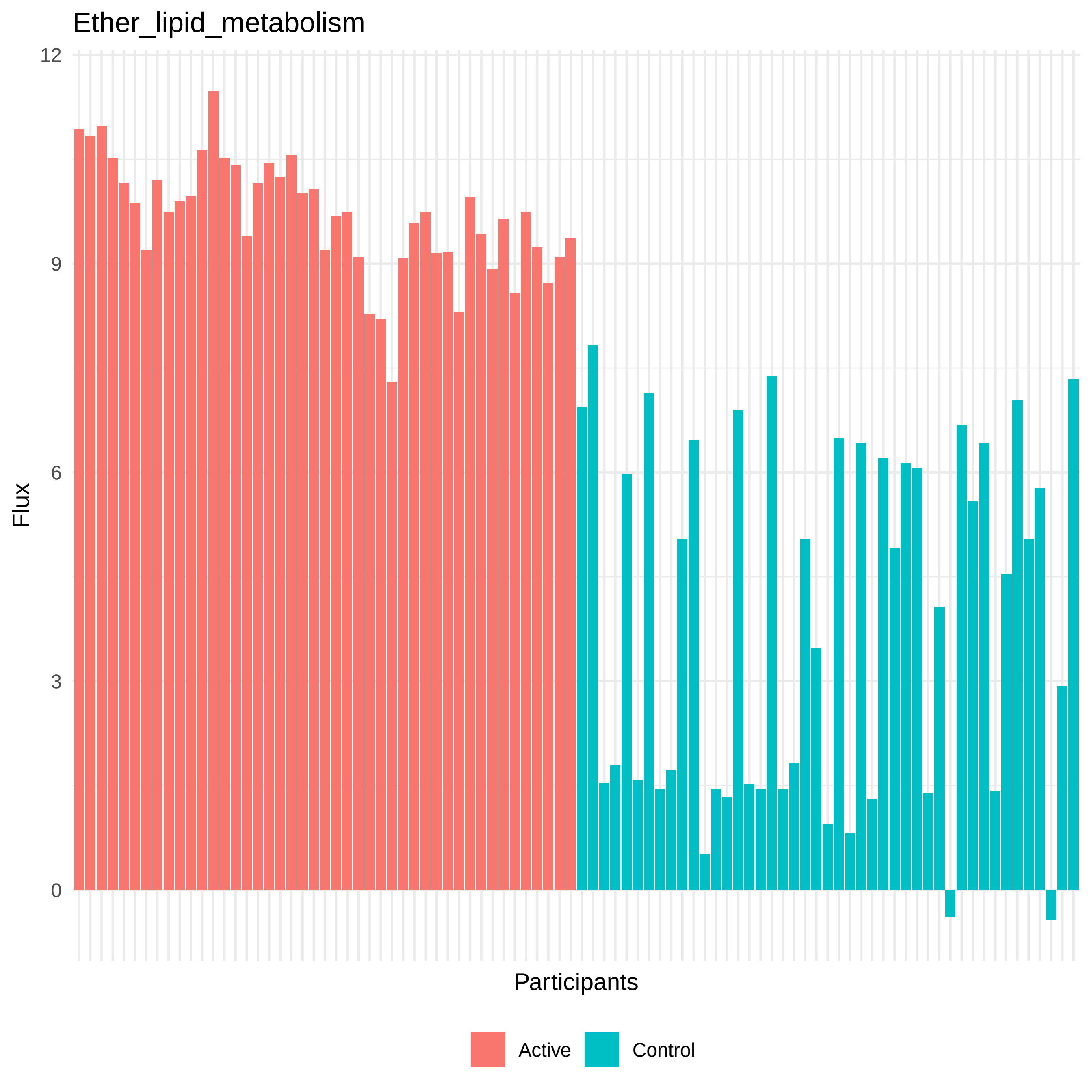

Ether_lipid_metabolism
12
9
6
3
0
Flux
Participants
Active
Control

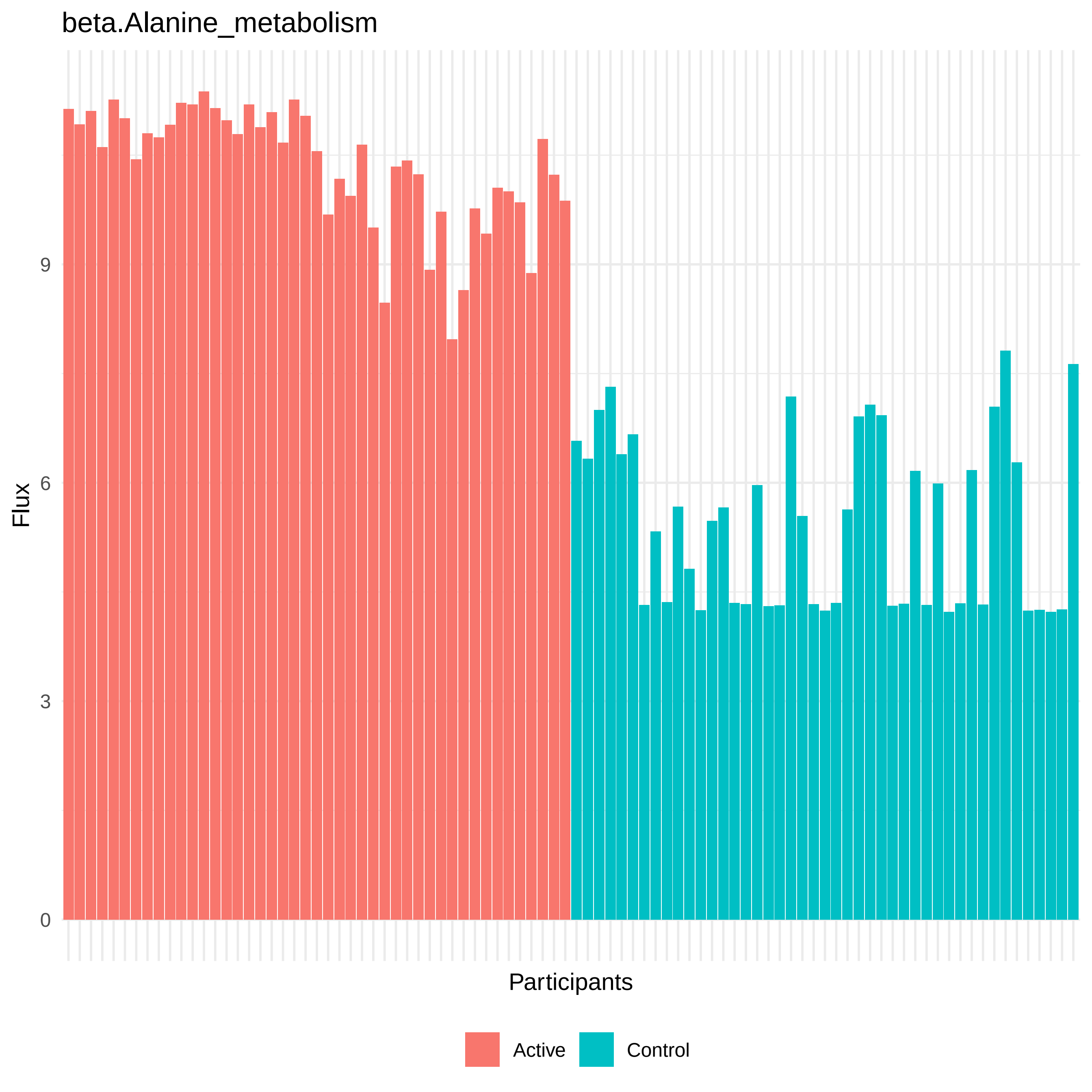
beta.Alanine_metabolism
Flux
0
3
6
9
Participants
Active
Control

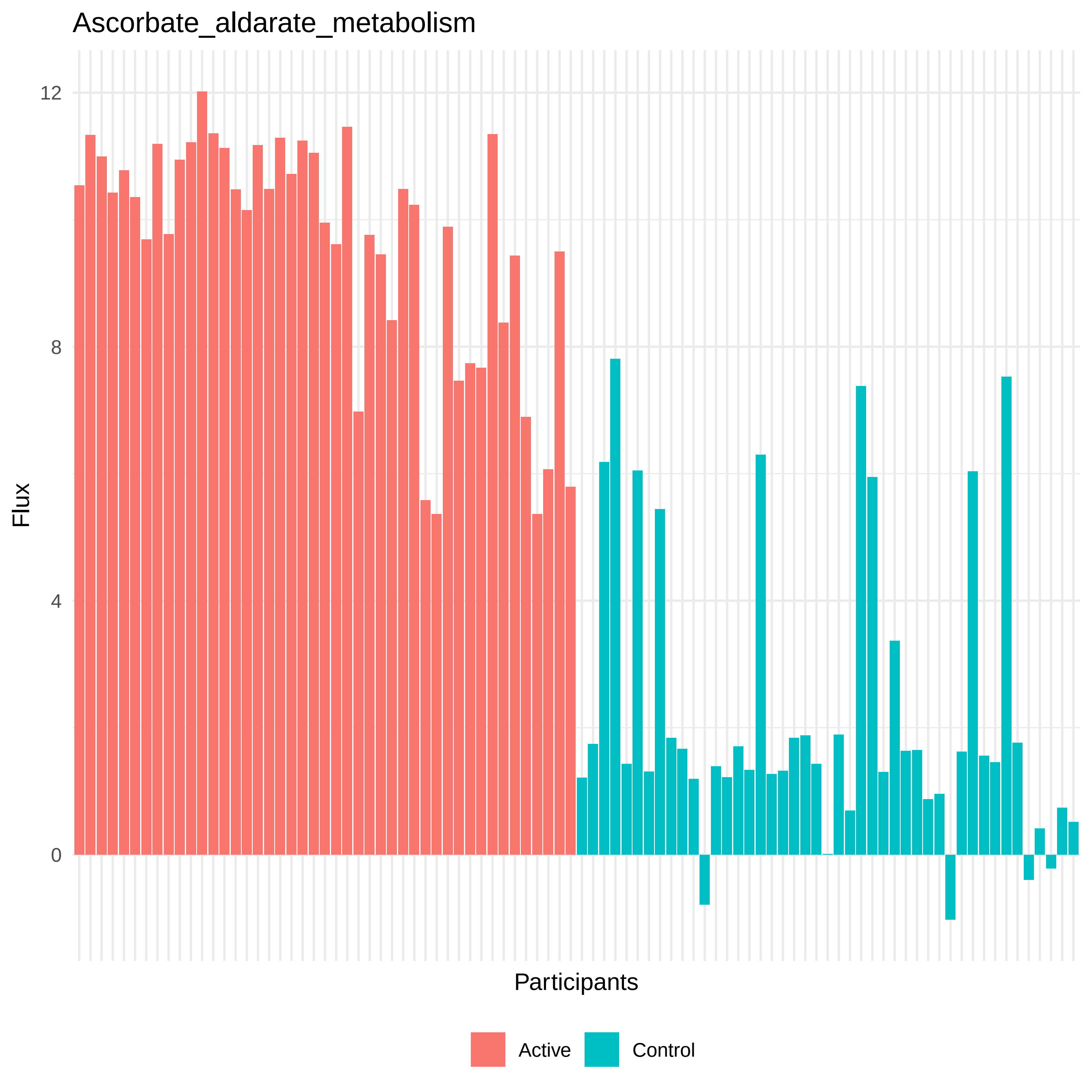

Ascorbate_aldarate_metabolism
12
8
4
0
Flux
Participants
Active
Control

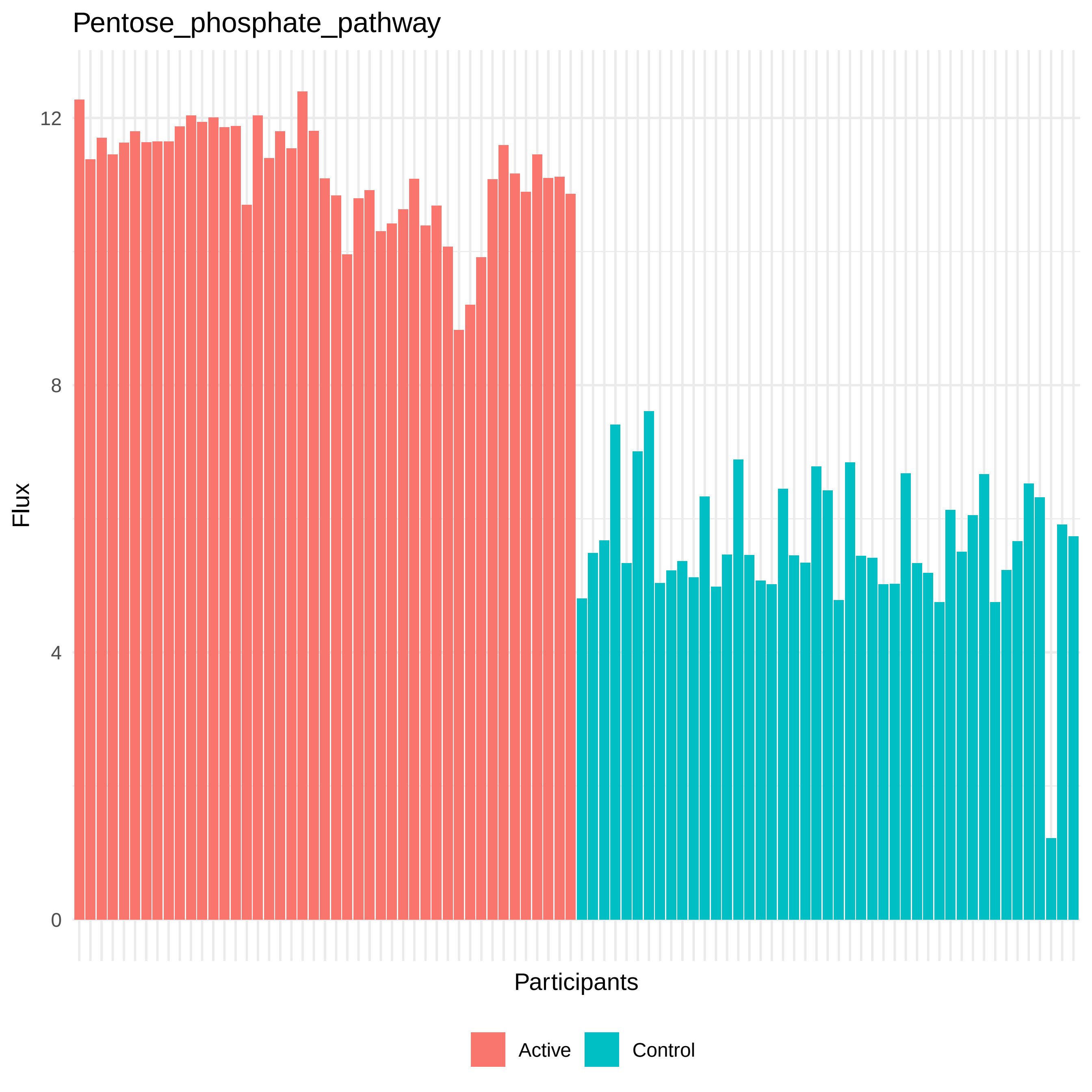

Pentose_phosphate_pathway
12
8
4
0
Flux
Participants
Active
Control

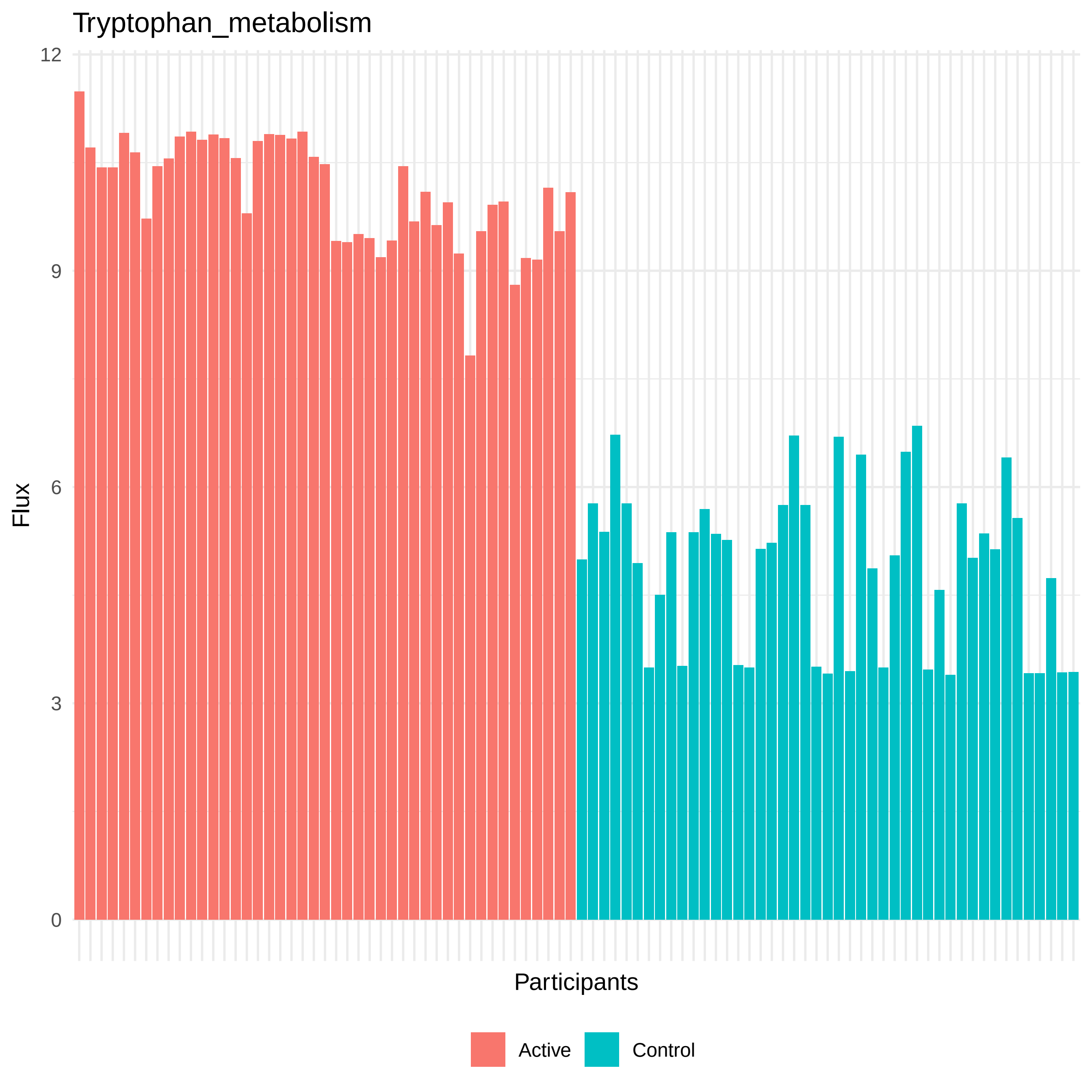
Tryptophan_metabolism
12
9
6
3
0
Flux
Participants
Active
Control

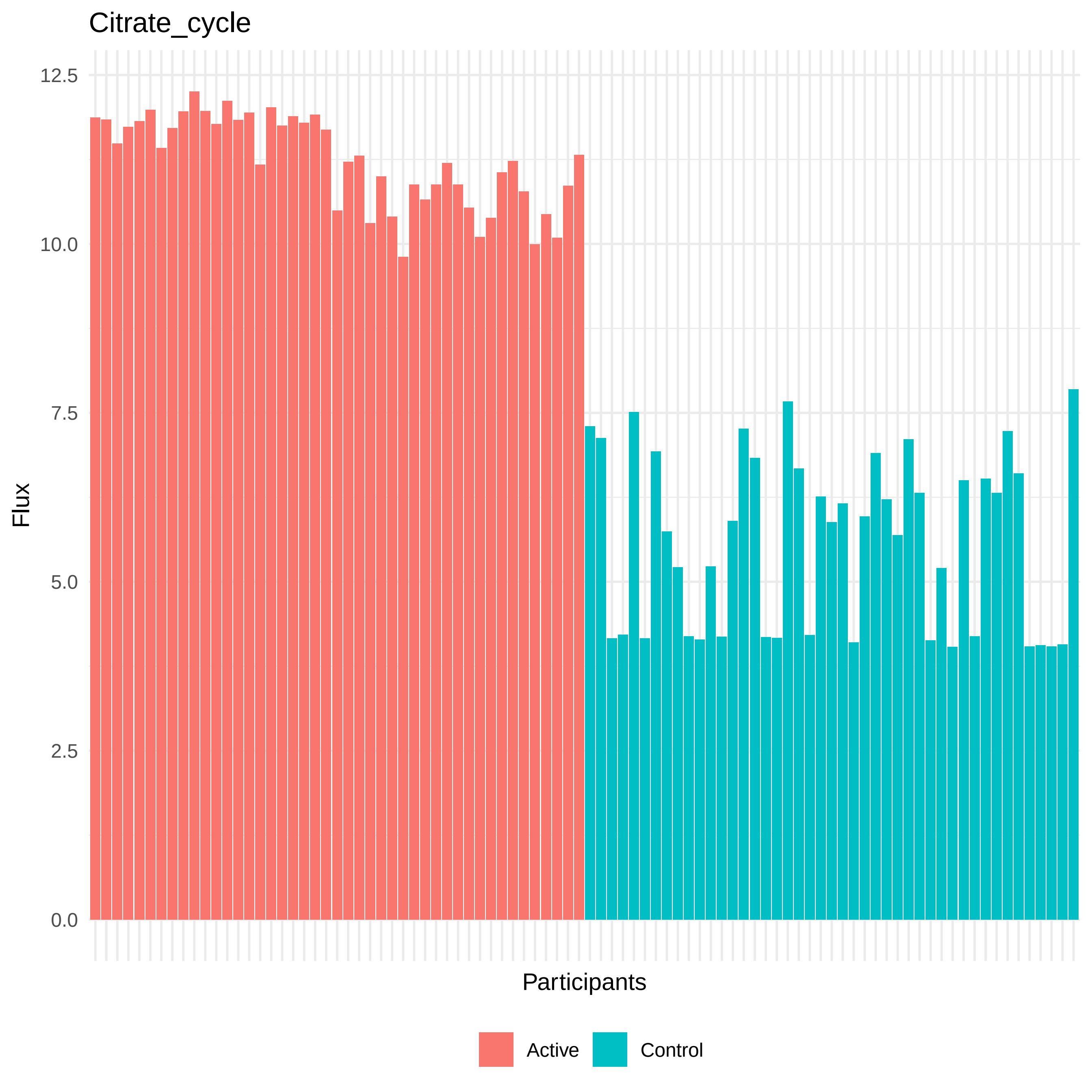

Citrate_cycle
12.5
10.0
7.5
5.0
2.5
0.0
Flux
Participants
Active
Control

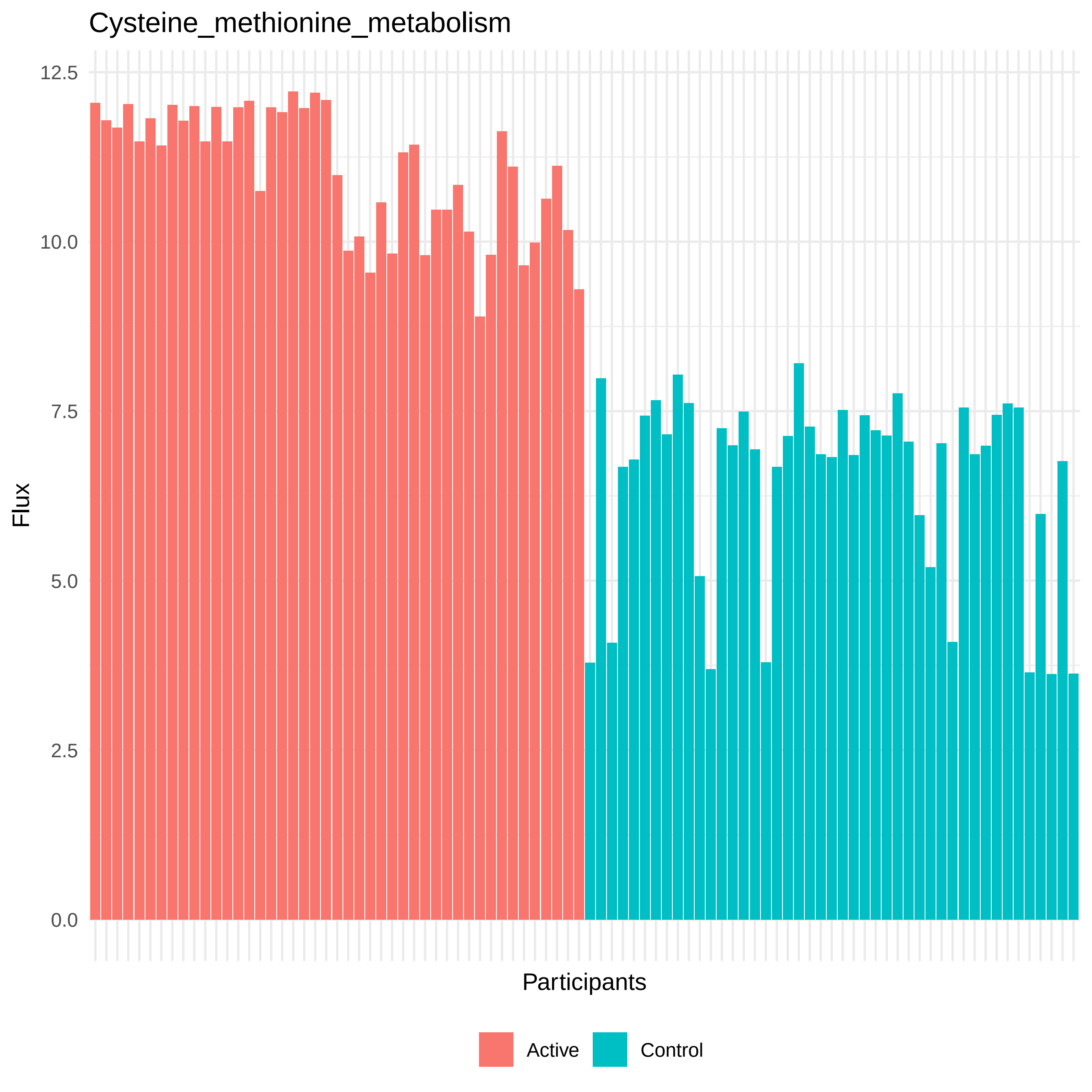
Cysteine_methionine_metabolism
12.5
10.0
7.5
5.0
2.5
0.0
Flux
Participants
Active
Control

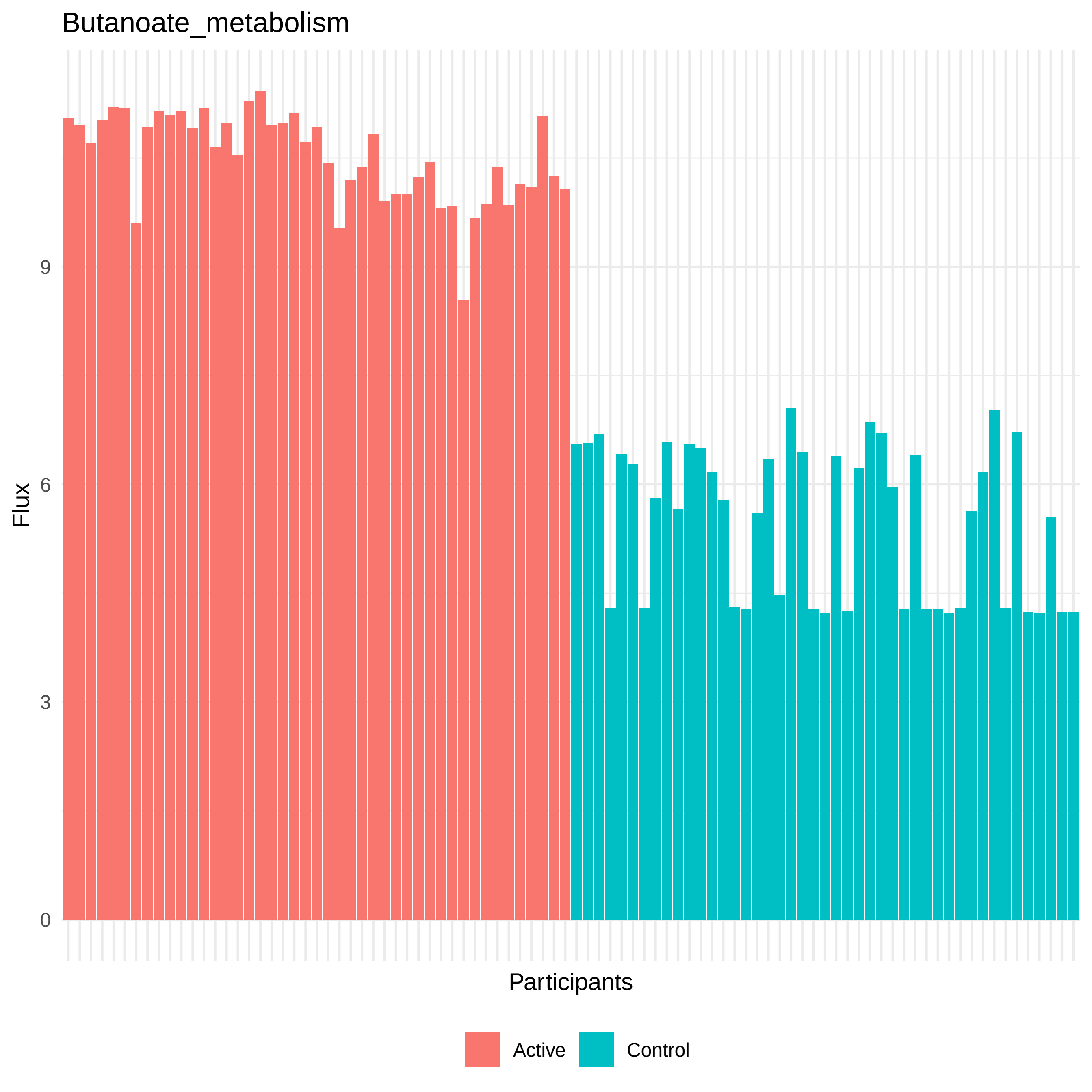

Butanoate_metabolism
Flux
9
6
3
0
Participants
Active
Control

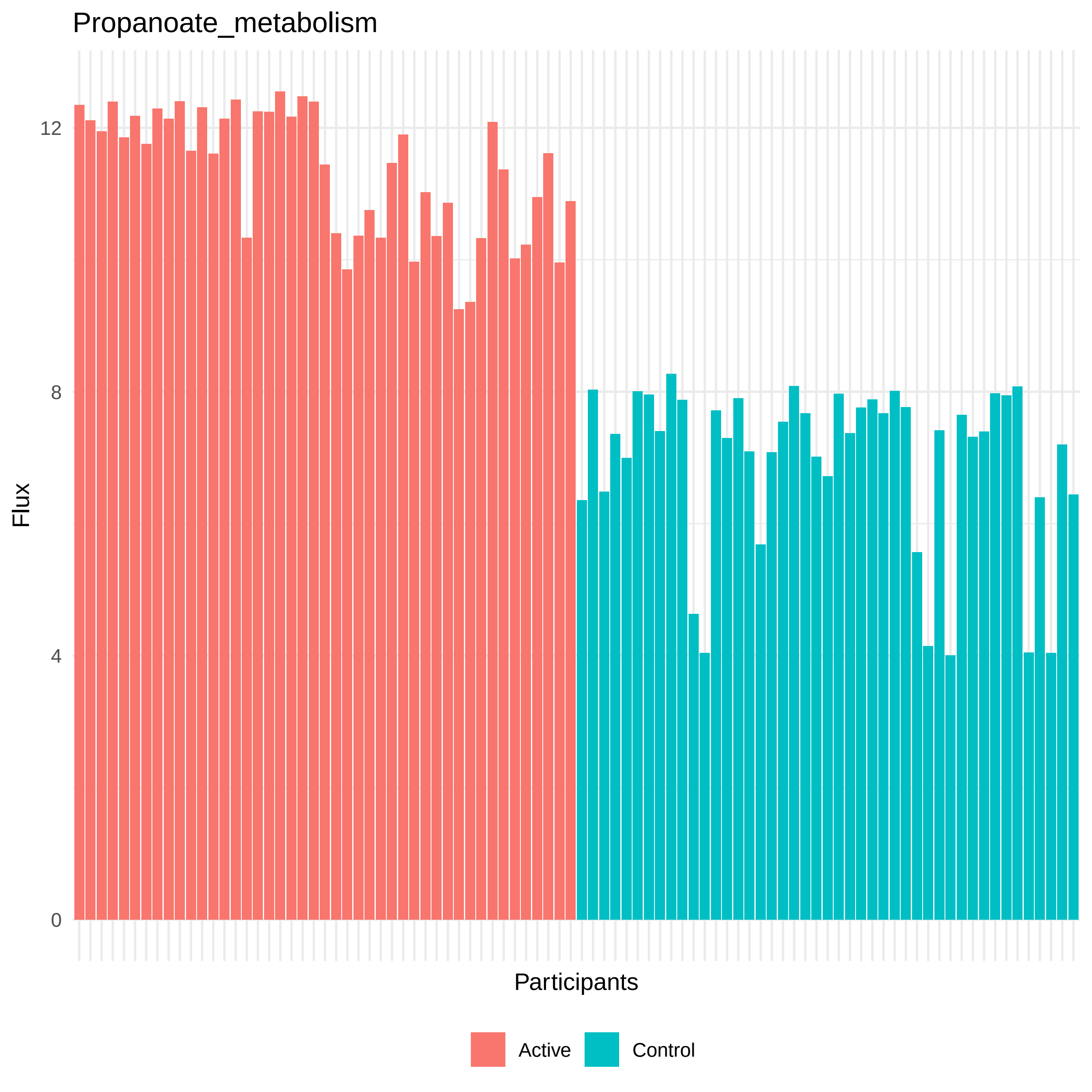

Propanoate_metabolism
Flux
12
8
4
0
Participants
Active
Control

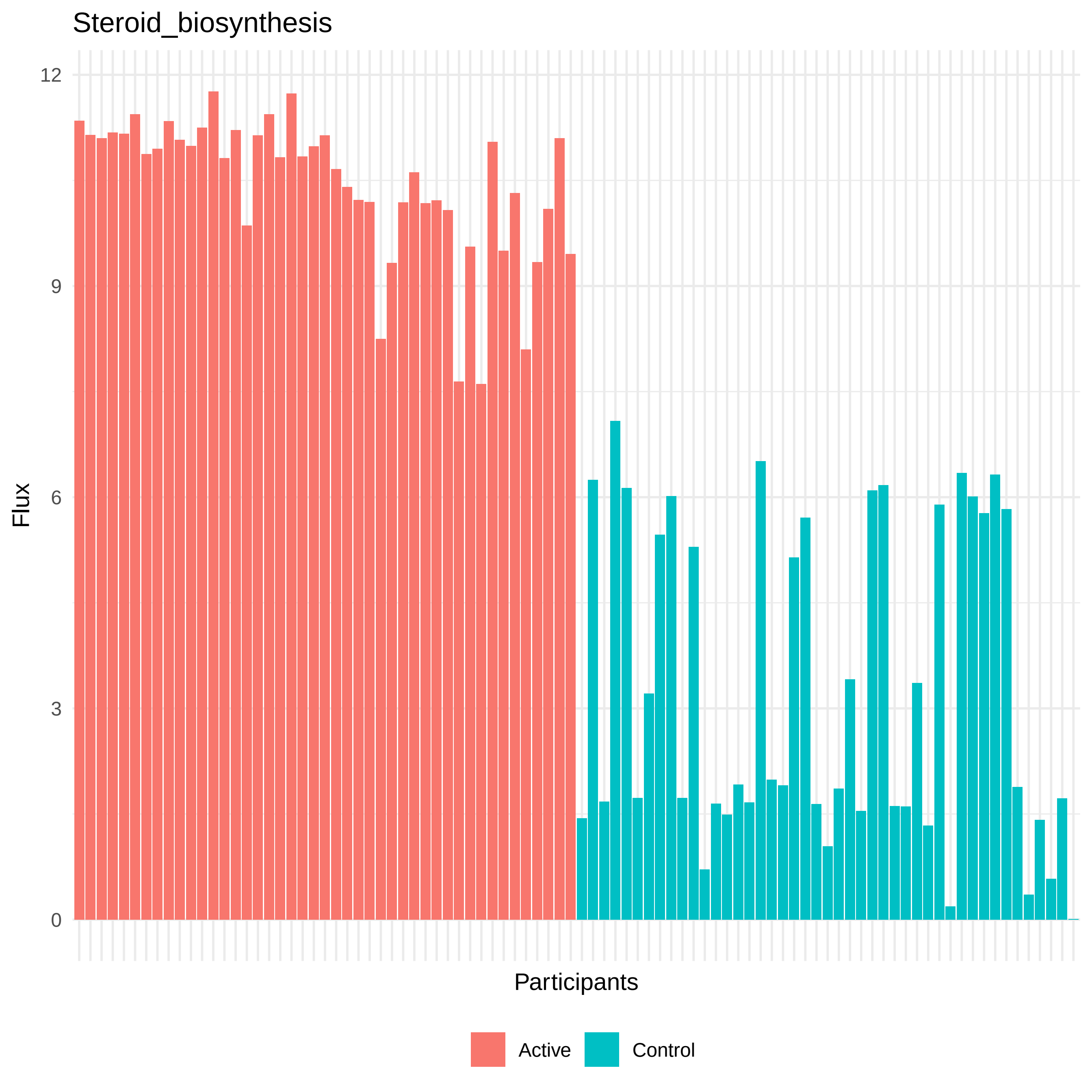

Steroid_biosynthesis
12
9
6
3
0
Flux
Participants
Active
Control

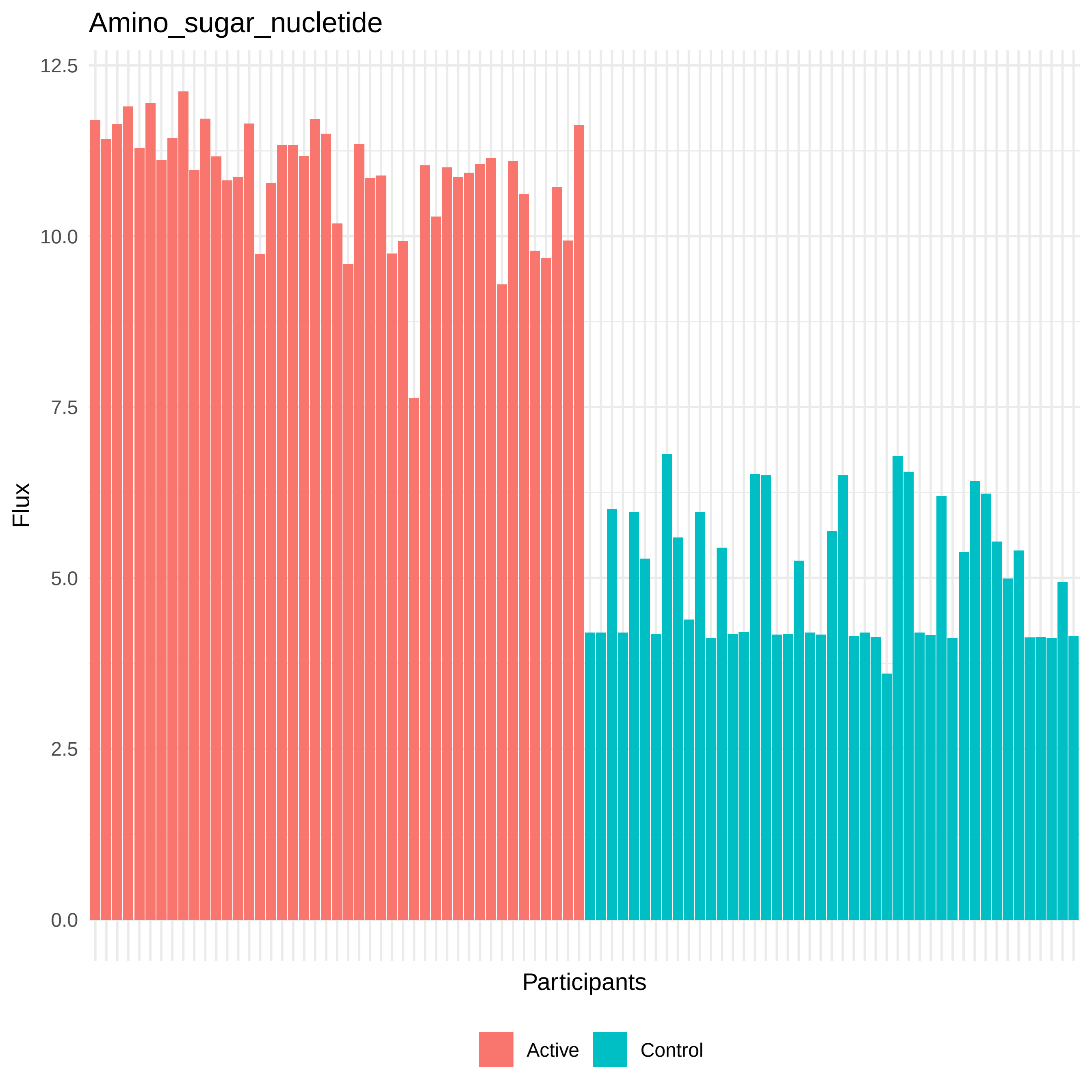
Amino_sugar_nucletide
Flux
12.5
10.0
7.5
5.0
2.5
0.0
Participants
Active
Control

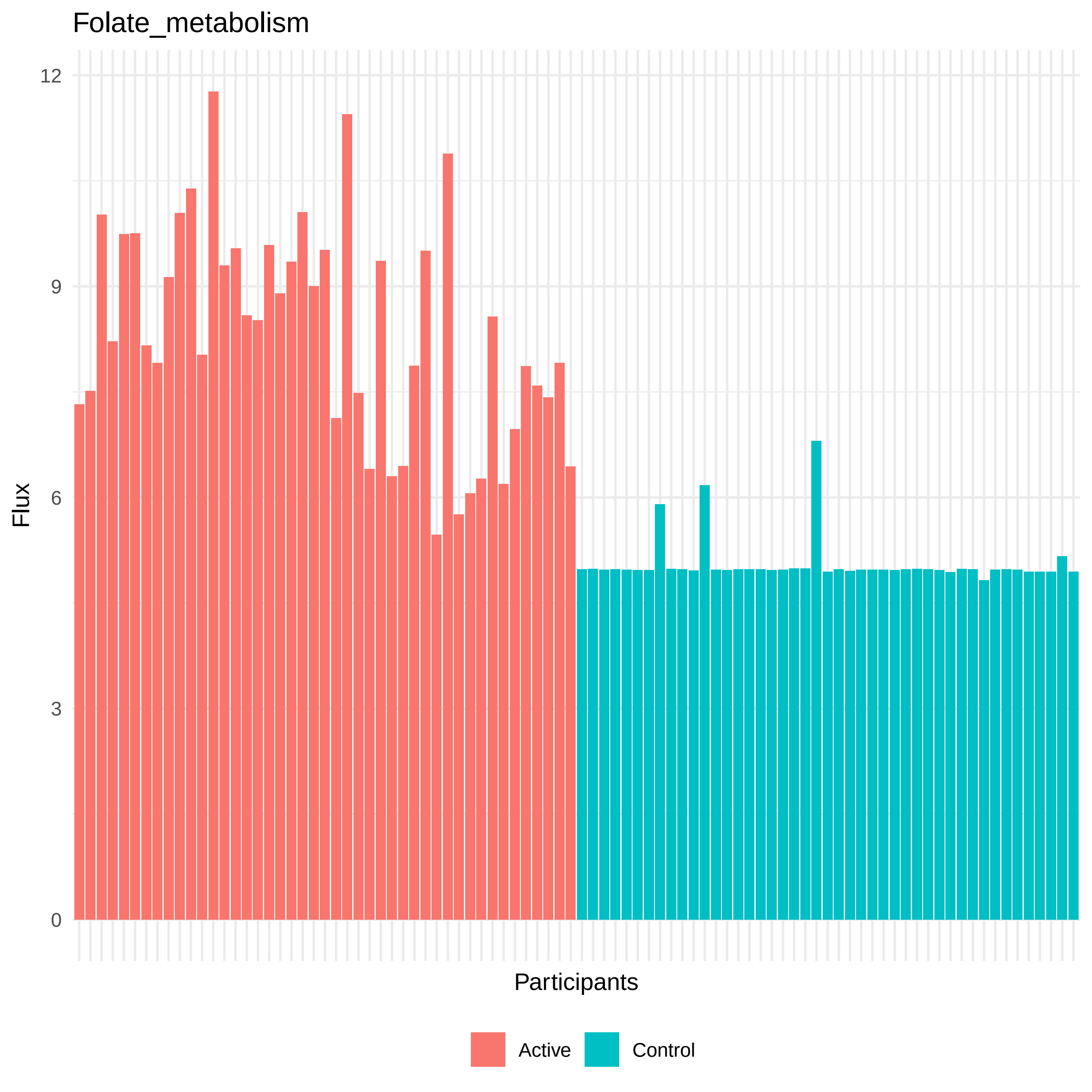

Folate_metabolism
Flux
12
9
6
3
0
Participants
Active
Control

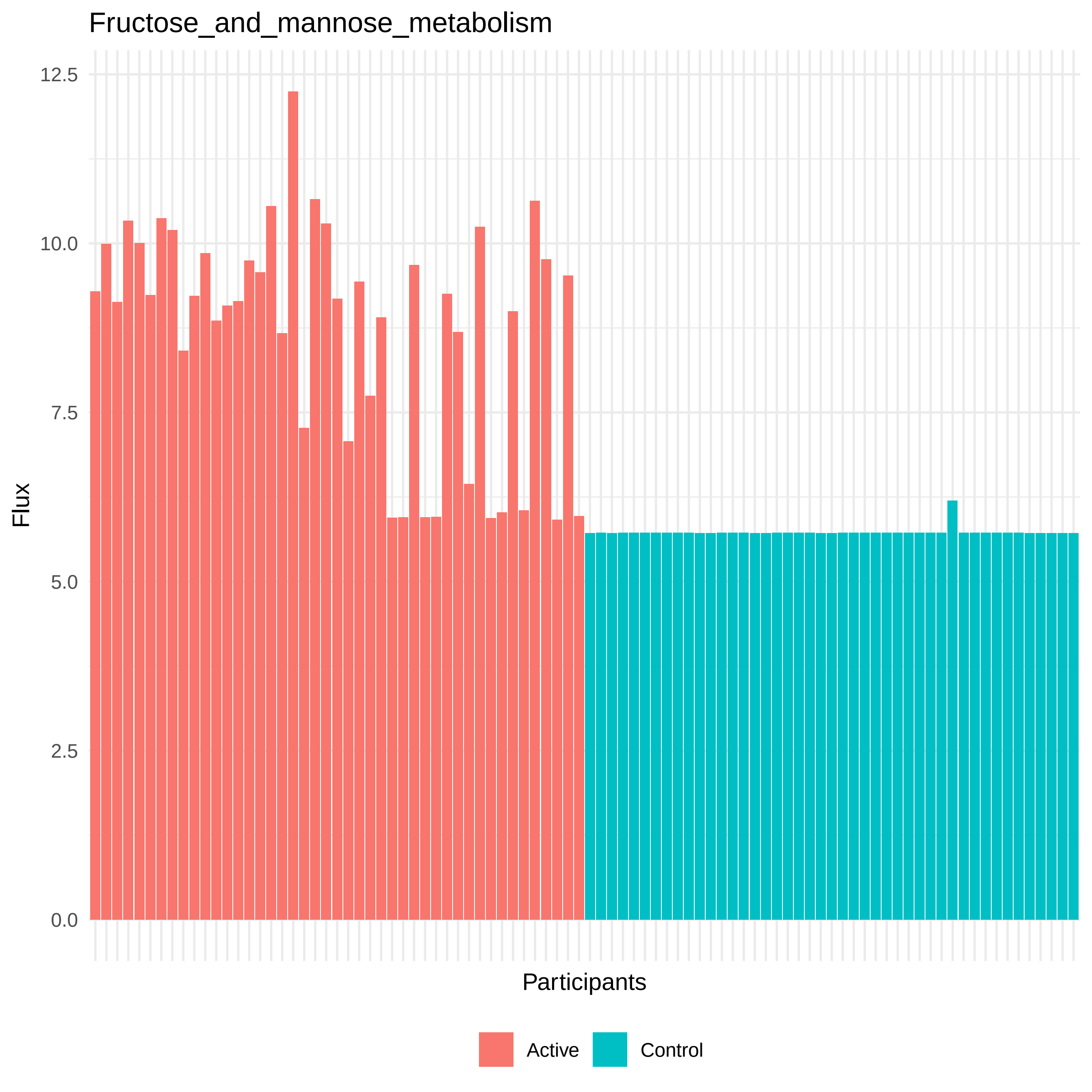

Fructose_and_mannose_metabolism
12.5
10.0
7.5
5.0
2.5
0.0
Flux
Participants
Active
Control

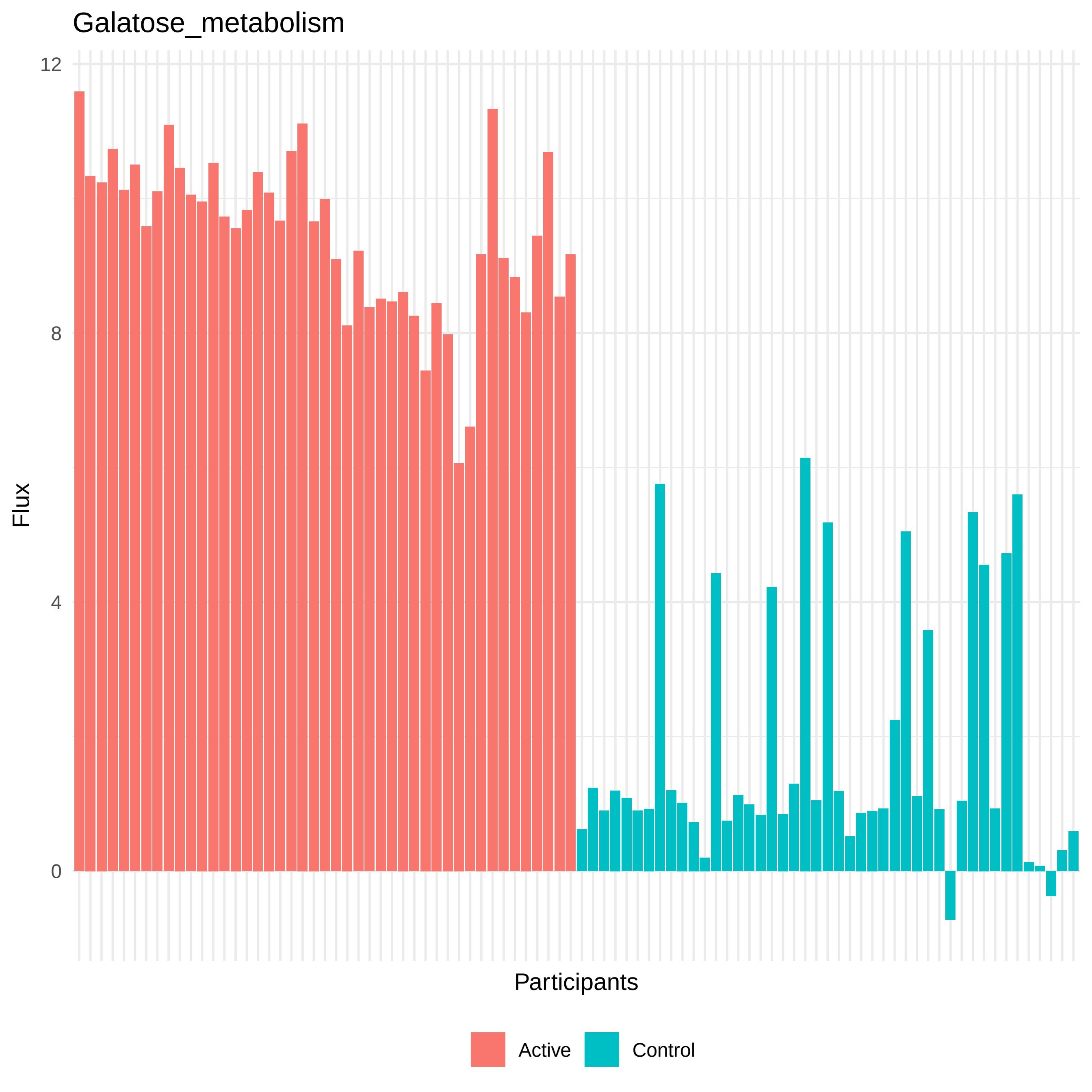
Galatose_metabolism
Flux
12
8
4
0
Participants
Active
Control

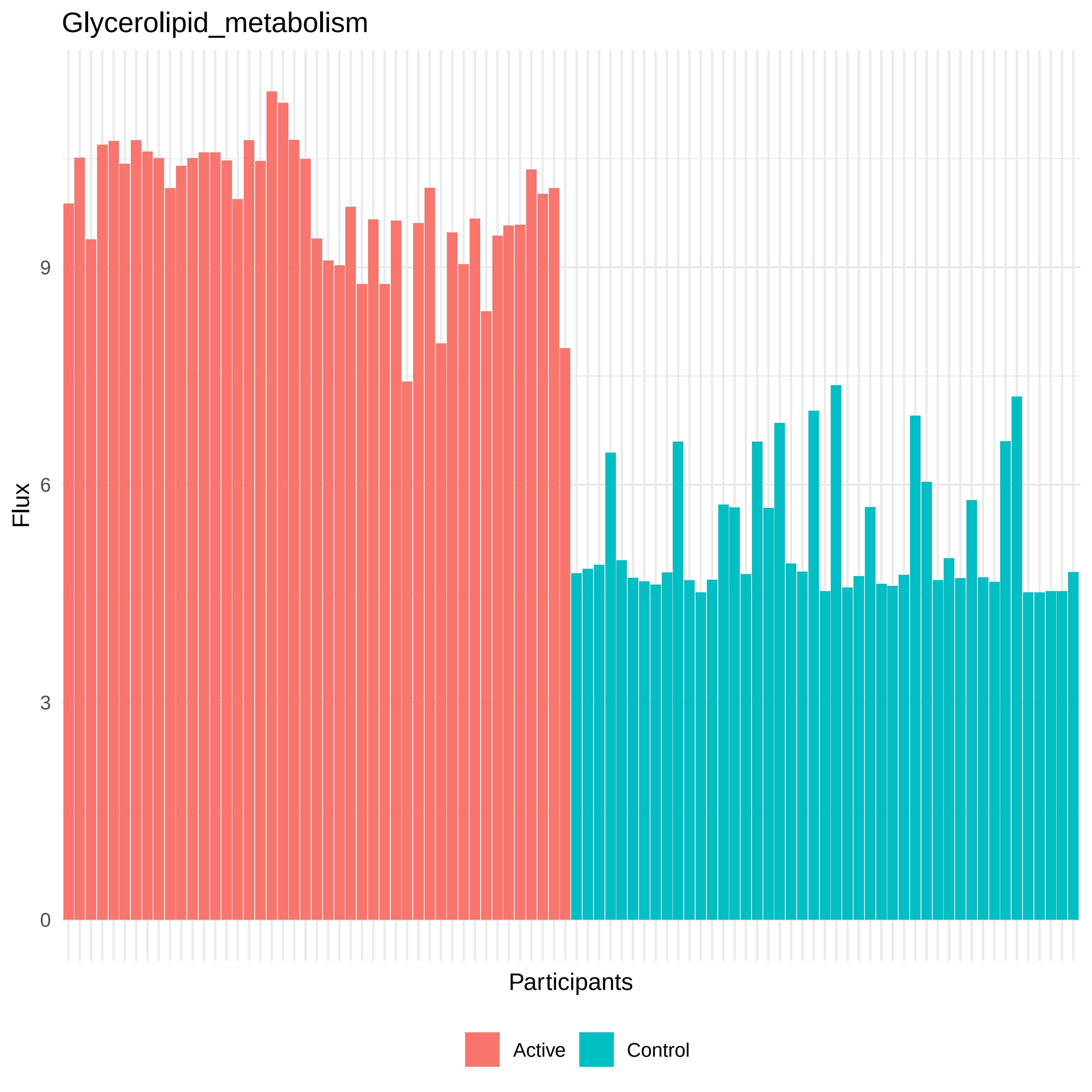

Glycerolipid_metabolism
Flux
9
6
3
0
Participants
Active
Control

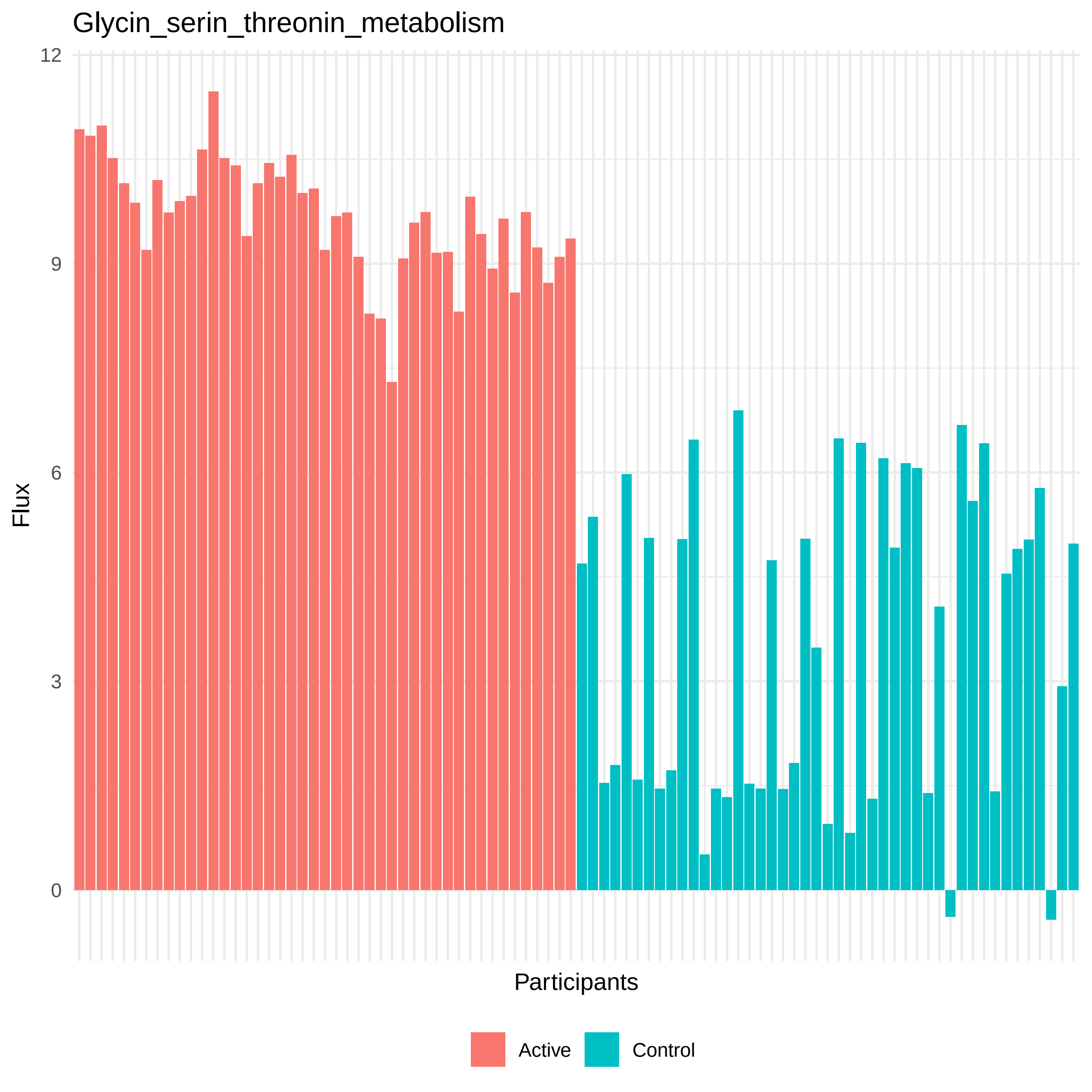

Glycin_serin_threonin_metabolism
12
9
6
3
0
Flux
Participants
Active
Control

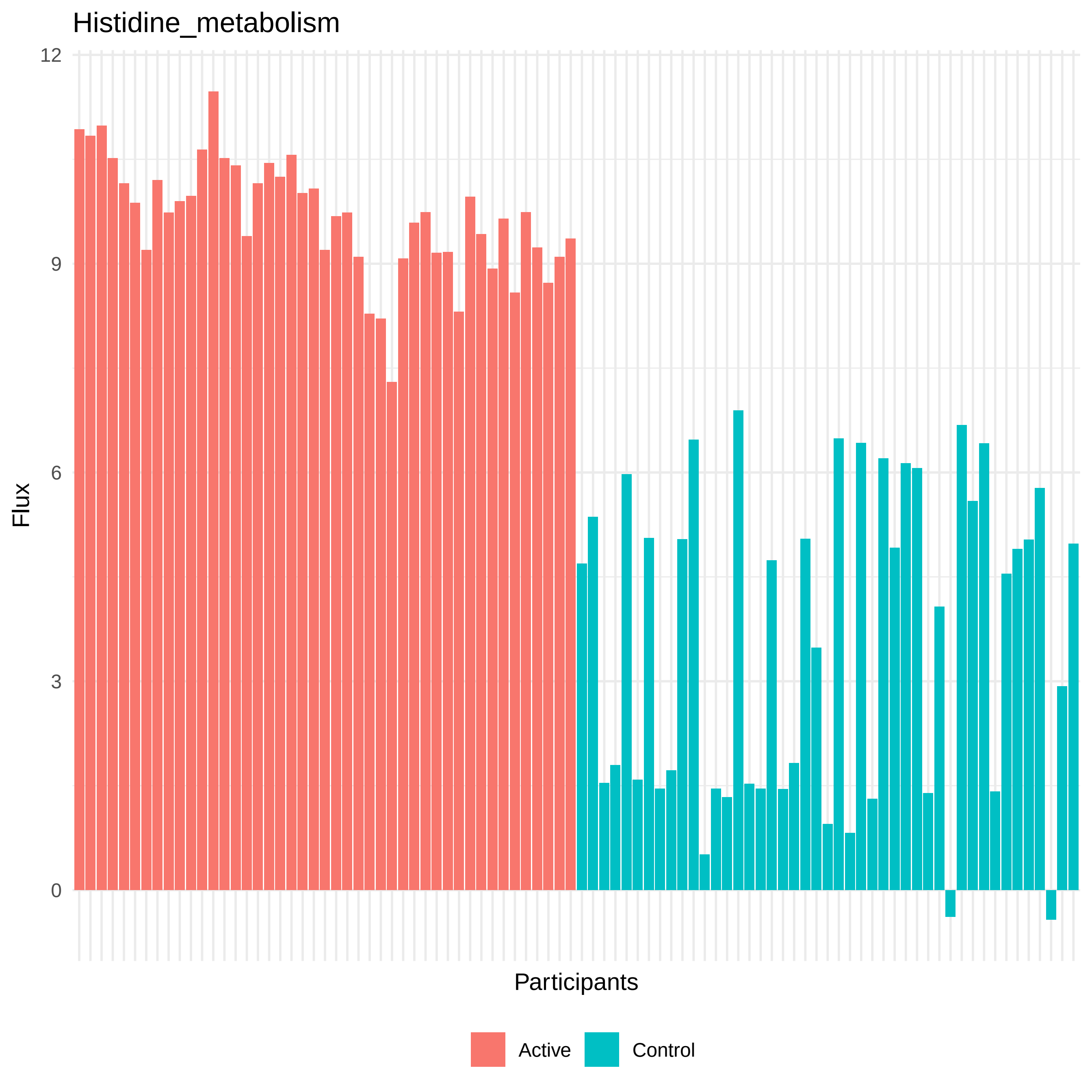

Histidine_metabolism
12
9
6
3
0
Flux
Participants
Active
Control

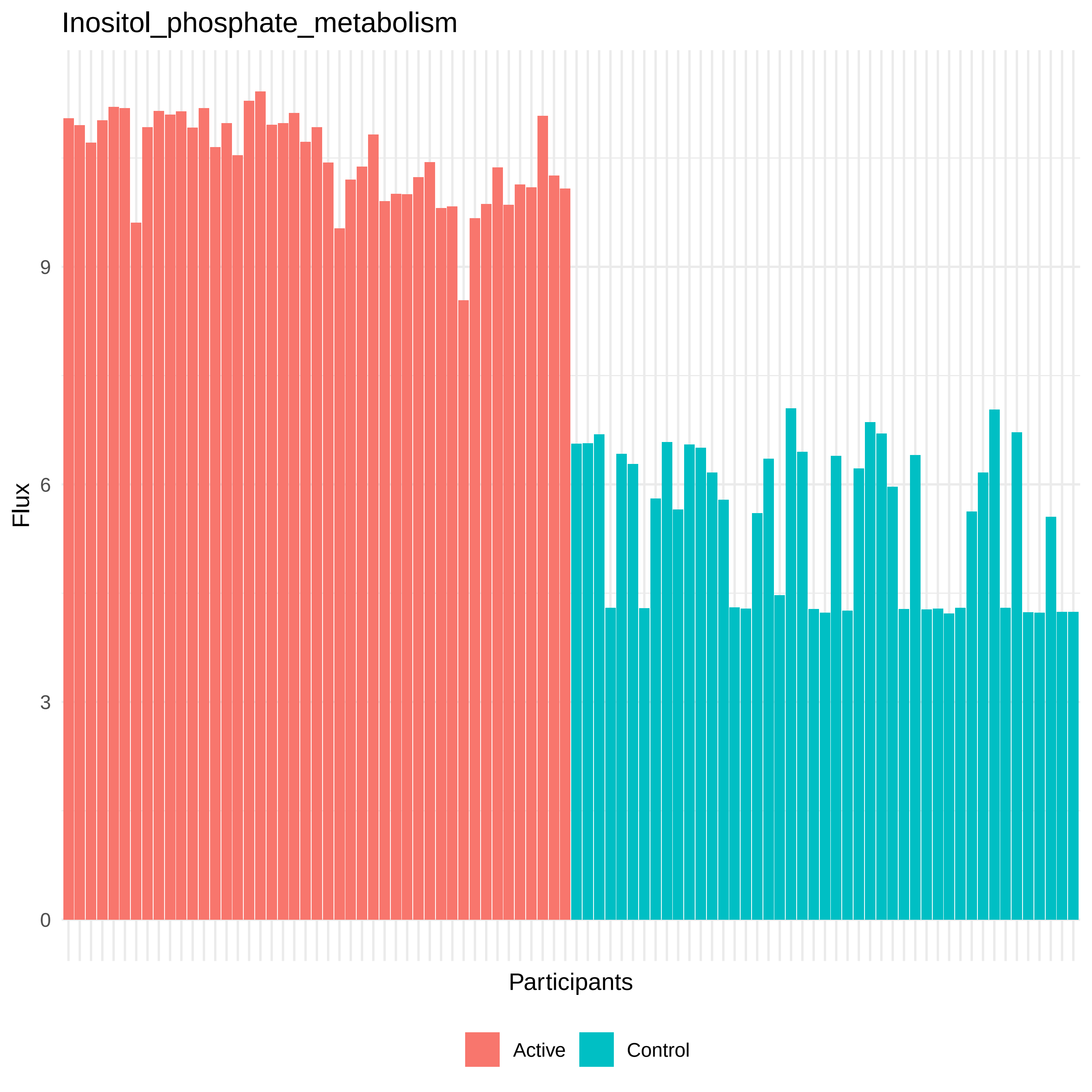
Inositol_phosphate_metabolism
Flux
9
6
3
0
Participants
Active
Control

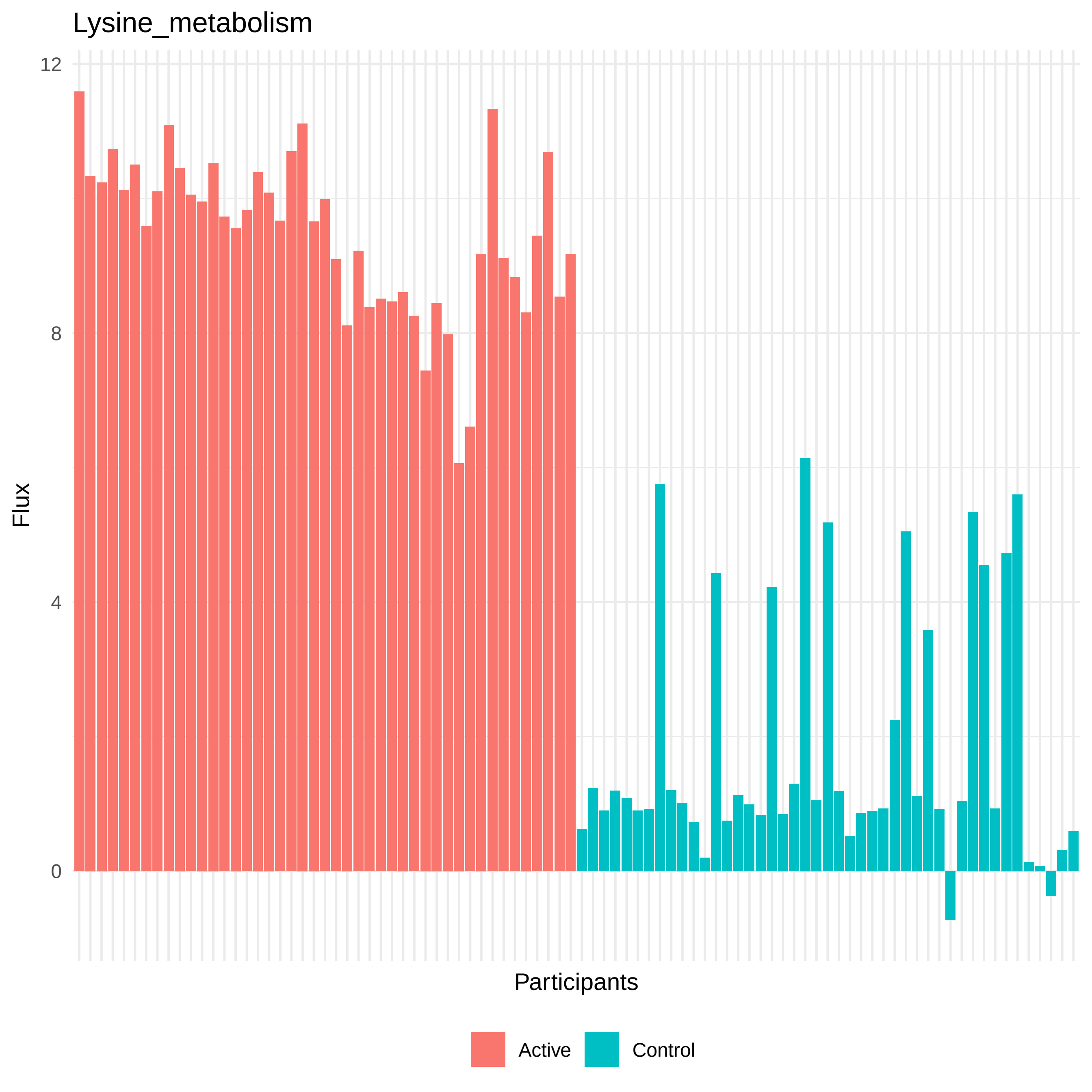
Lysine_metabolism
12
8
4
0
Flux
Participants
Active
Control

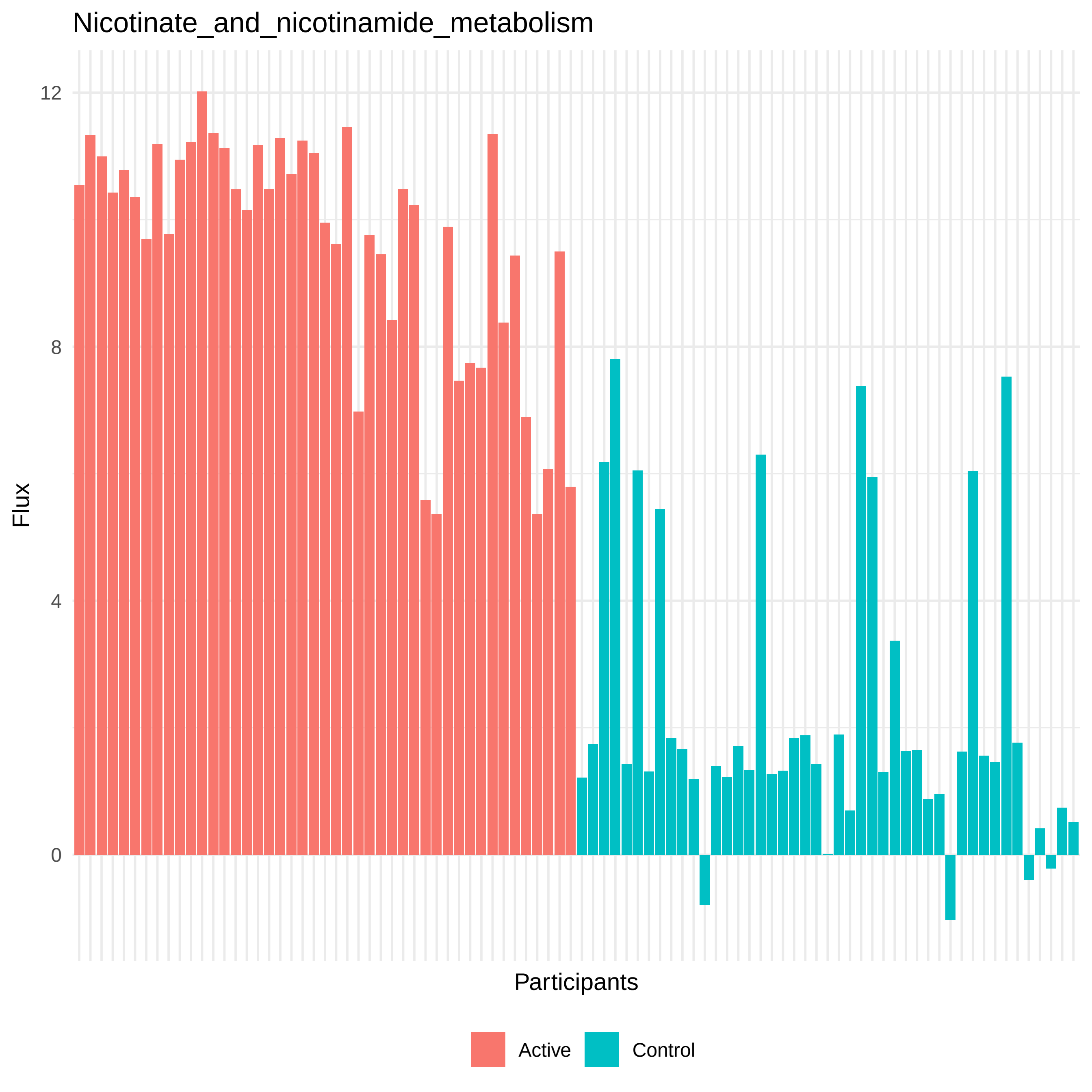
Nicotinate_and_nicotinamide_metabolism
12
8
4
0
Flux
Participants
Active
Control

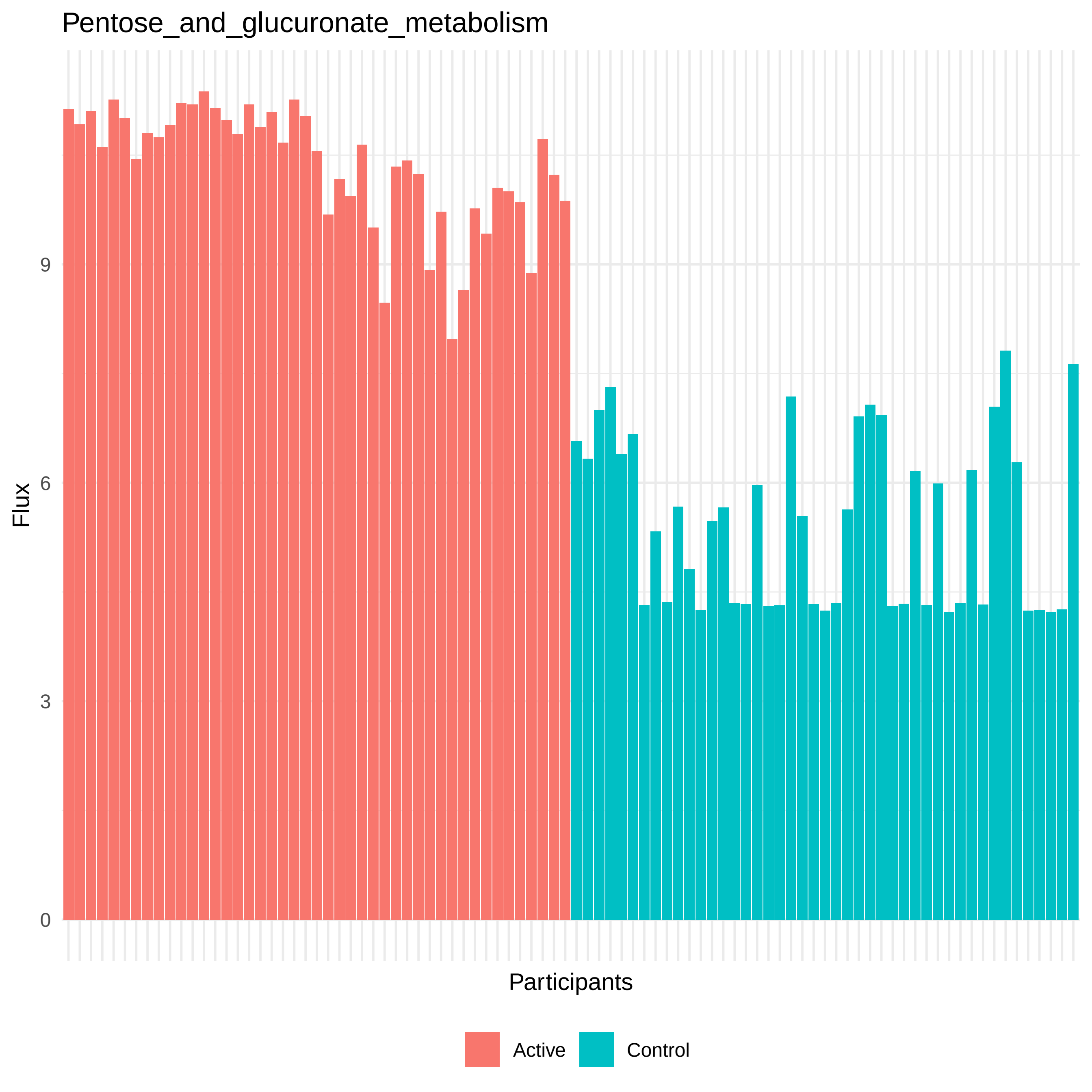
Pentose_and_glucuronate_metabolism
Flux
9
6
3
0
Participants
Active
Control

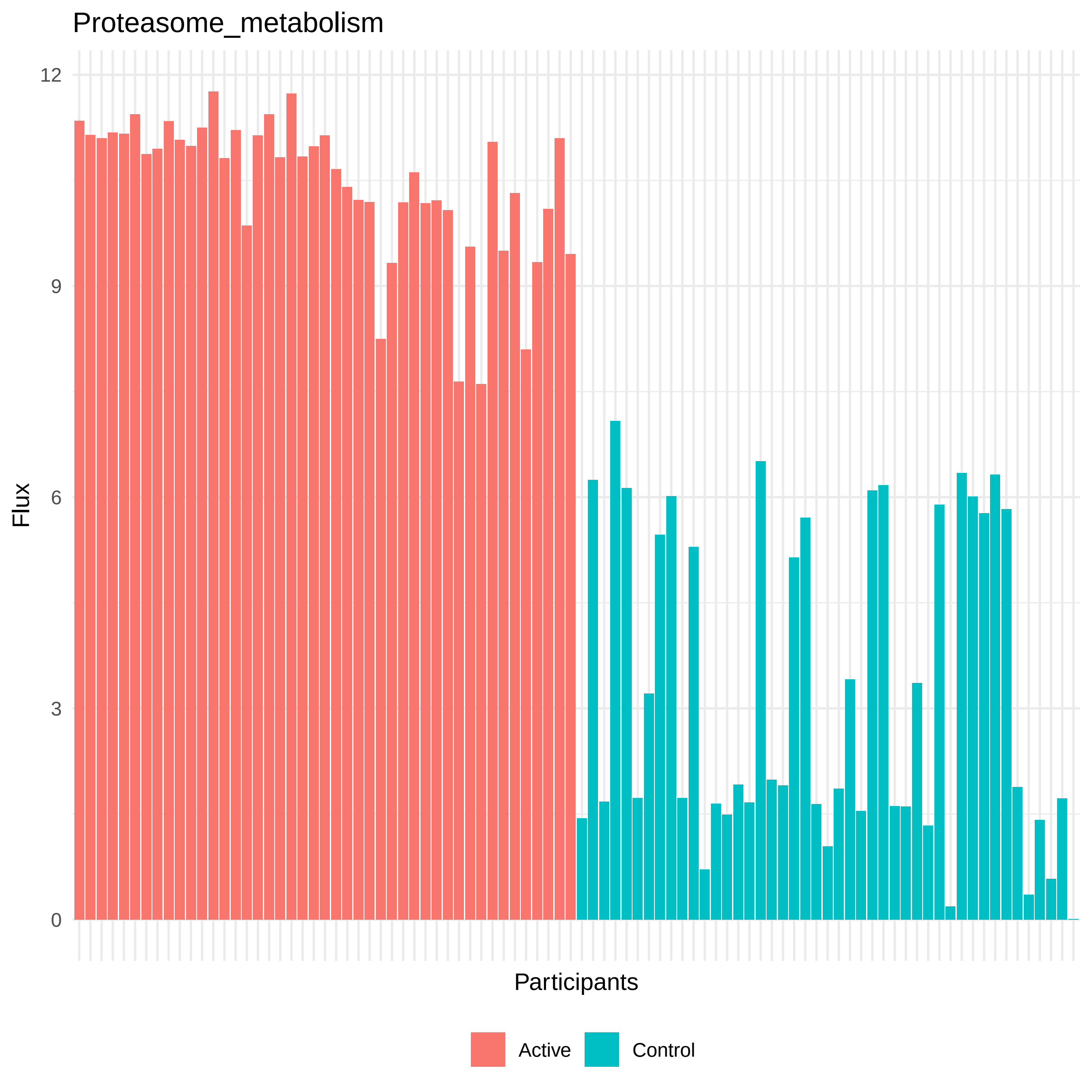

Proteasome_metabolism
12
9
6
3
0
Flux
Participants
Active
Control

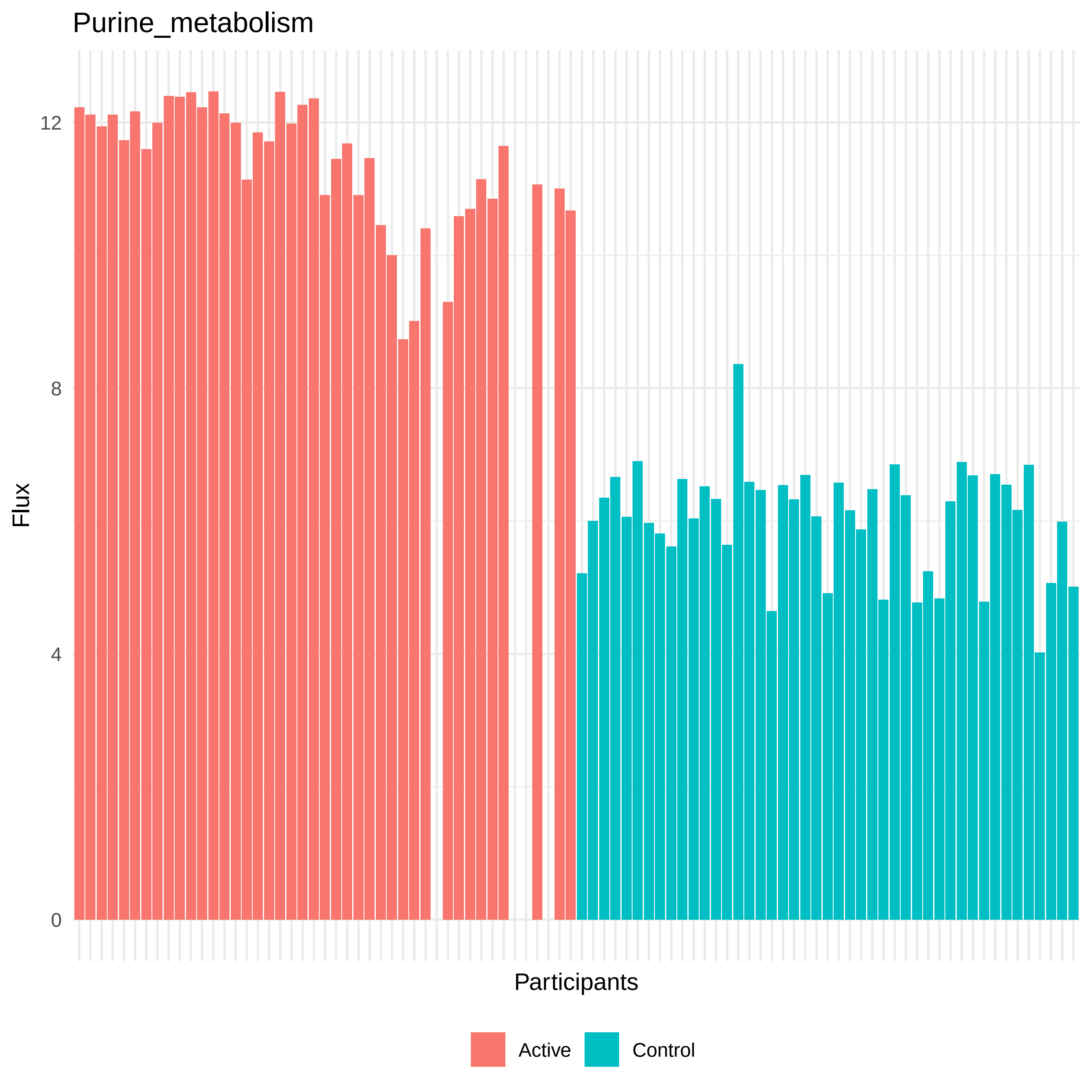

Purine_metabolism
12
8
4
0
Flux
Participants
Active
Control

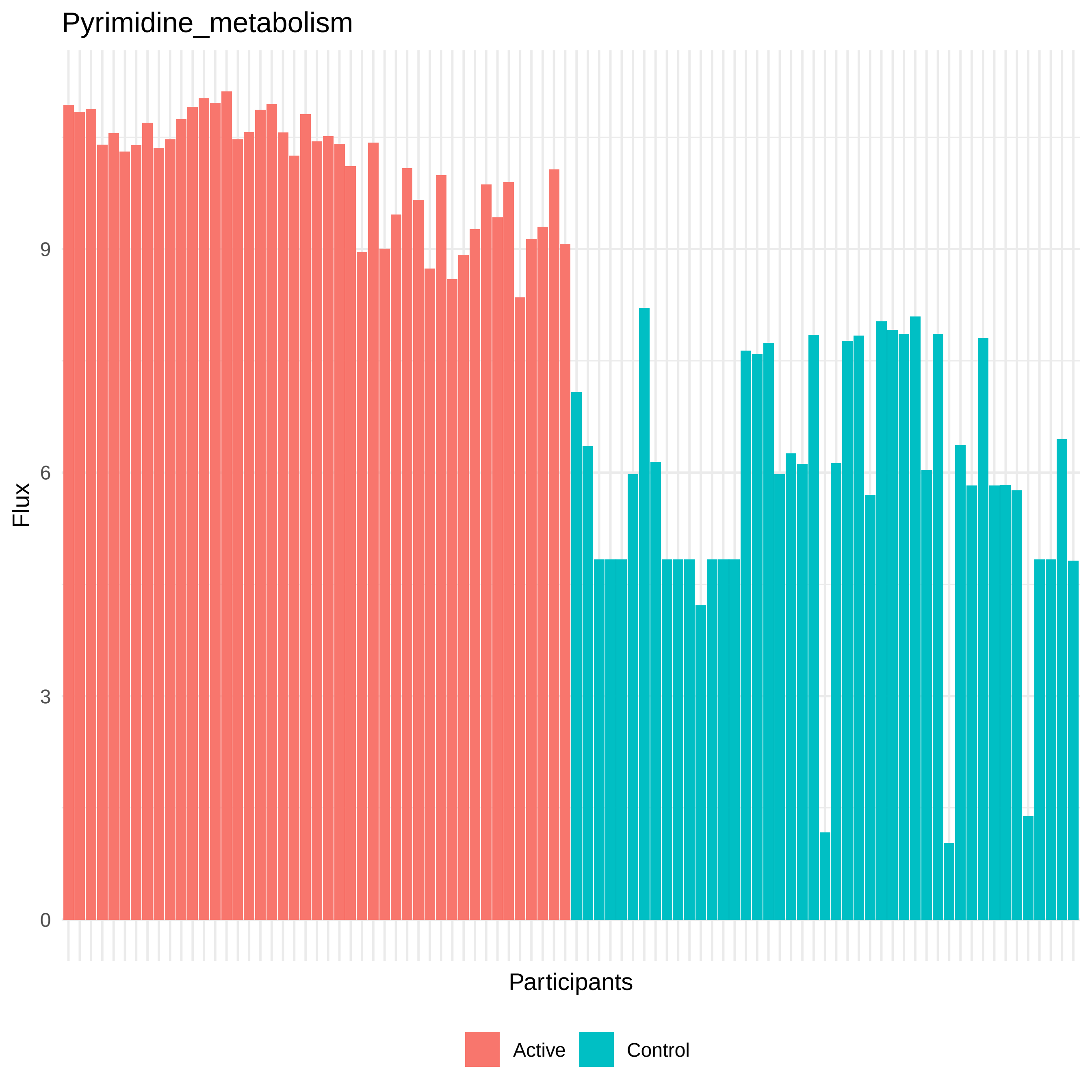

Pyrimidine_metabolism
Flux
9
6
3
0
Participants
Active
Control

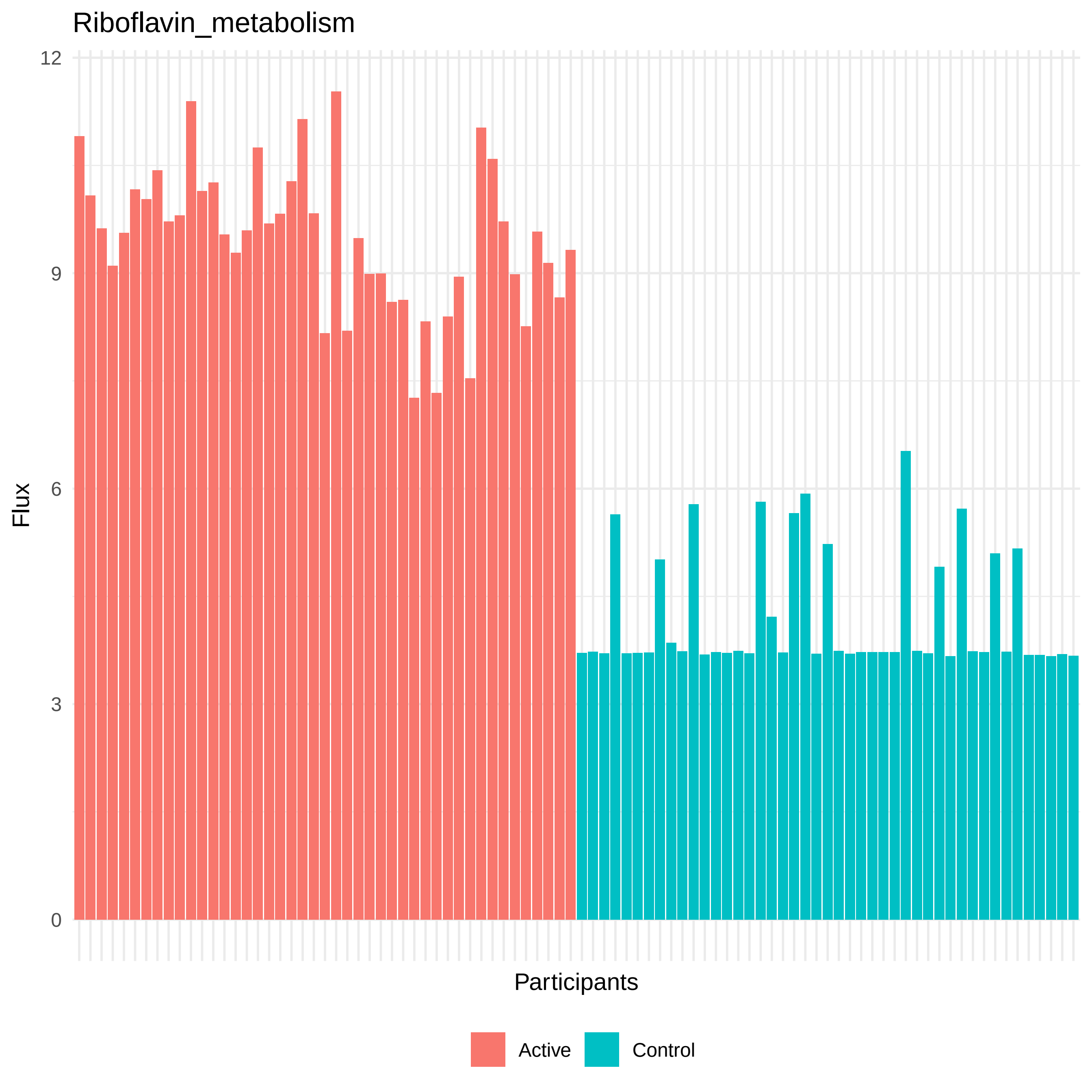

Riboflavin_metabolism
Flux
12
9
6
3
0
Participants
Active
Control

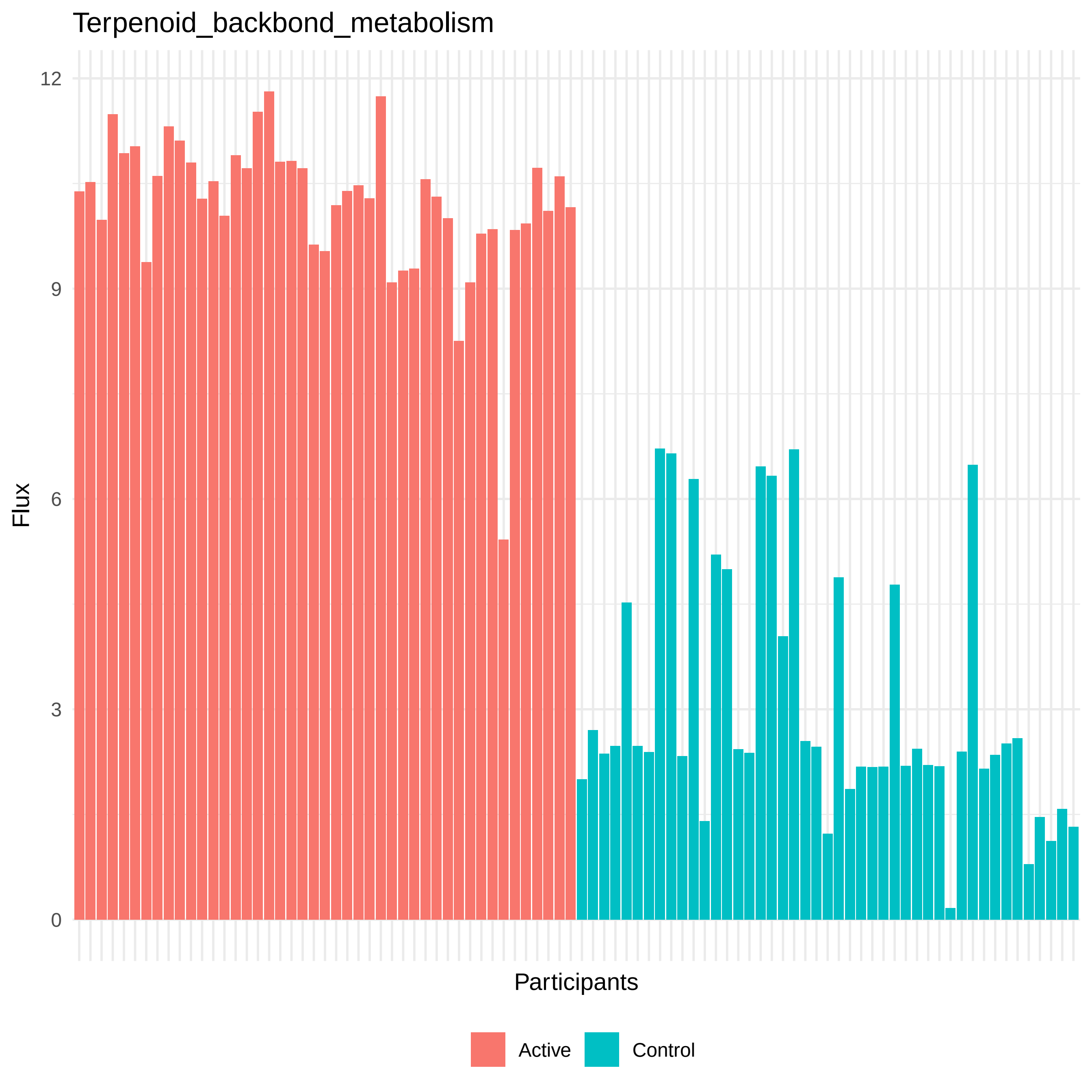

Terpenoid_backbond_metabolism
Flux
12
9
6
3
0
Participants
Active
Control

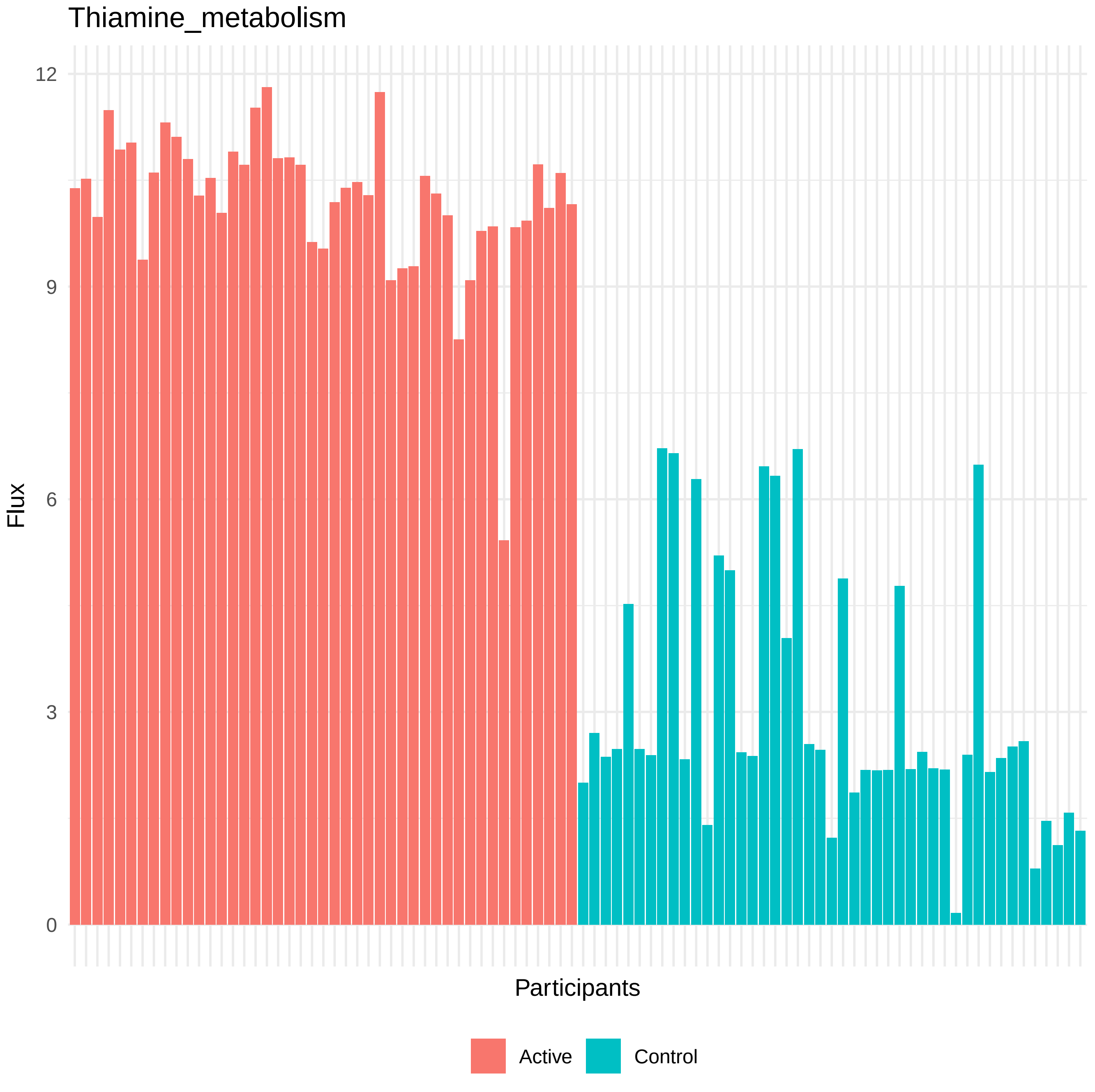
Thiamine_metabolism
Flux
12
9
6
3
0
Participants
Active
Control

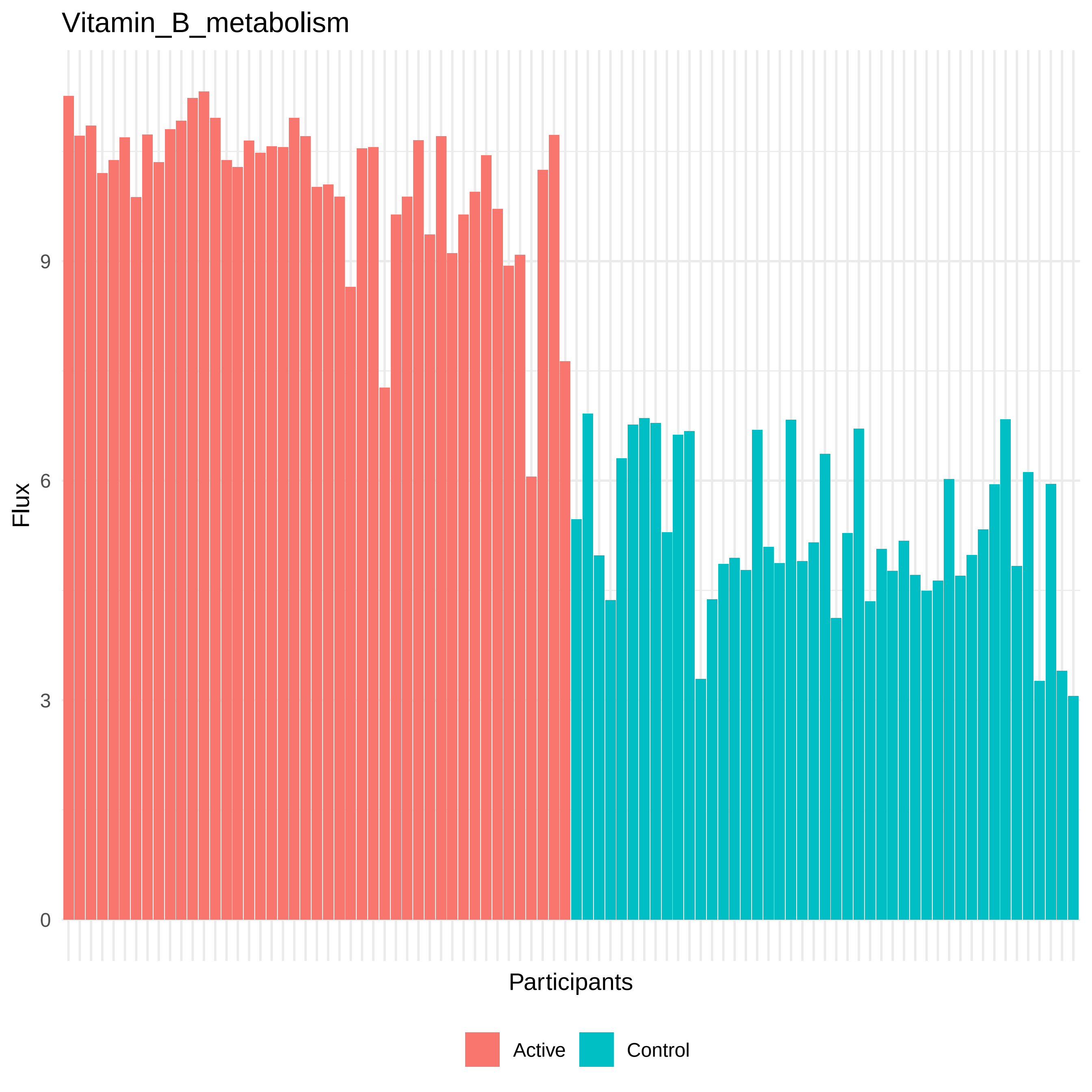

Vitamin_B_metabolism
9
6
3
0
Flux
Participants
Active
Control

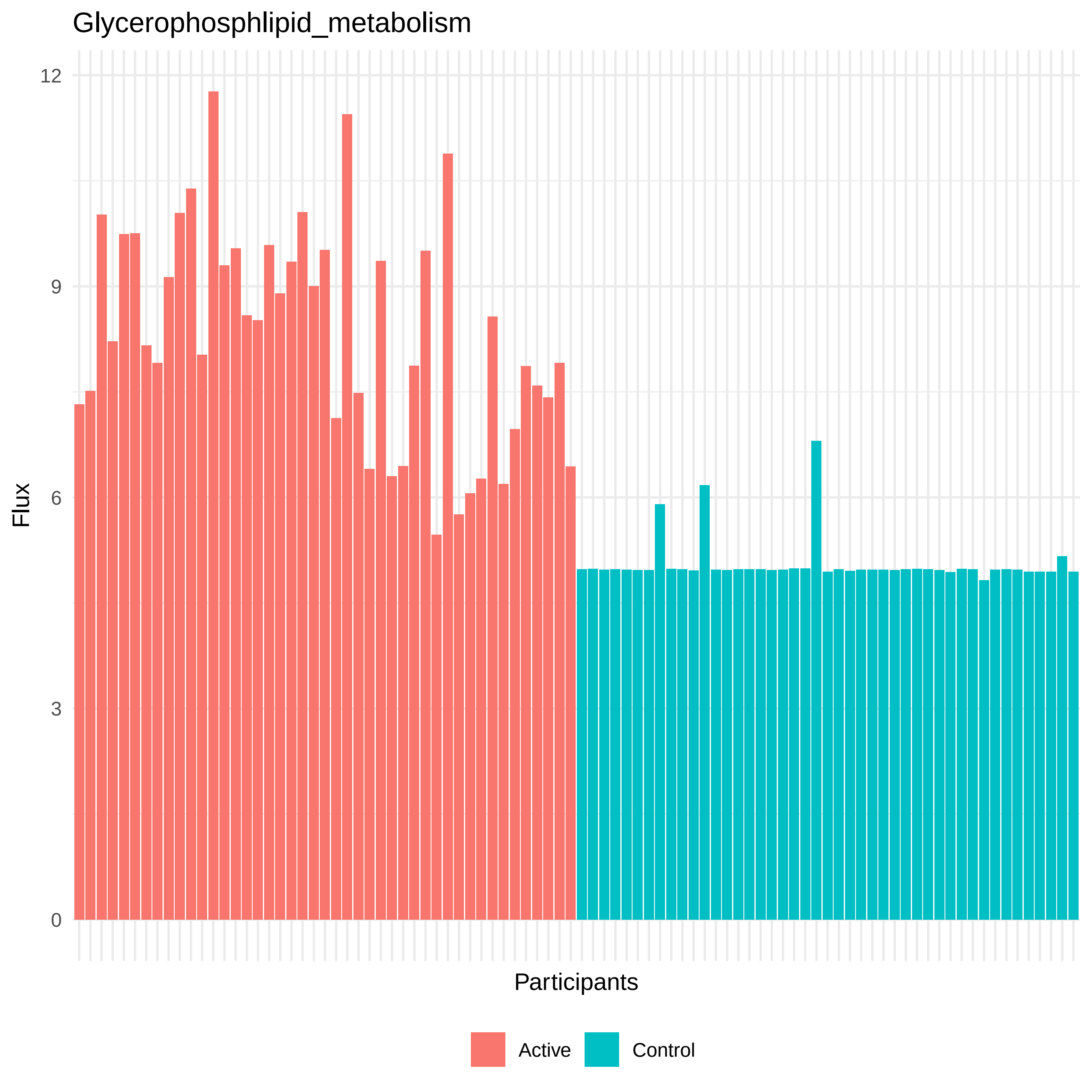

Glycerophosphlipid_metabolism
Flux
12
9
6
3
0
Participants
Active
Control
